# S-acyl transferase ZDHHC13 modulates tumor microenvironment interactions to suppress metastasis in melanoma models

**DOI:** 10.1172/JCI188249

**Published:** 2025-09-30

**Authors:** Hongjin Li, Jianke Lyu, Yu Sun, Chengqian Yin, Yuewen Li, Weiqiang Chen, Suan-Sin Foo, Xianfang Wu, Colin R. Goding, Shuyang Chen

**Affiliations:** 1 Department of Cancer Biology and; 2 Department of Cardiovascular & Metabolic Sciences, Lerner Research Institute, Cleveland Clinic, Cleveland, Ohio, USA.; 3 Department of Pharmacology and Experimental Therapeutics, Boston University School of Medicine, Boston, Massachusetts, USA.; 4 Infection Biology Program, Lerner Research Institute, Cleveland Clinic, Cleveland, Ohio, USA.; 5 Ludwig Institute for Cancer Research, University of Oxford, Headington, Oxford, United Kingdom.

**Keywords:** Immunology, Oncology, Immunotherapy, Molecular biology, Skin cancer

## Abstract

The intratumor microenvironment shapes the metastatic potential of cancer cells and their susceptibility to any immune response. Yet, the nature of the signals within the microenvironment that control anticancer immunity and how they are regulated is poorly understood. Here, using melanoma as a model, we investigate the involvement in metastatic dissemination and the immune-modulatory microenvironment of Protein S-Acyl Transferases as an underexplored class of potential therapeutic targets. We find that ZDHHC13 suppresses metastatic dissemination by palmitoylation of CTNND1, leading to stabilization of E-cadherin. Importantly, ZDHHC13 also reshapes the tumor immune microenvironment by suppressing lysophosphatidylcholine (LPC) synthesis in melanoma cells, leading to inhibition of M2-like tumor-associated macrophages that we show degrade E-cadherin via MMP12 expression. Consequently, ZDHHC13 activity suppresses tumor growth and metastasis in immunocompetent mice. Our study highlights the therapeutic potential of targeting the ZDHHC13–E-cadherin axis and its downstream metabolic and immune-modulatory mechanisms, offering additional strategies to inhibit melanoma progression and metastasis.

## Abstract 2

ZDHHC13 suppresses melanoma metastasis by stabilizing E-cadherin and reshaping the immune microenvironment, offering a potential therapeutic target to inhibit tumor progression.

## Introduction

Metastatic dissemination and therapy resistance are the leading causes of cancer-related mortality. While genetic mutations contribute to resistance, the tumor microenvironment also shapes tumor progression and therapeutic response (1–4). Microenvironmental cues can drive reversible phenotypic transitions that promote immune evasion and metastatic outgrowth (5–7). Accordingly, therapeutic strategies are increasingly aimed at both blocking metastasis and overcoming microenvironment-driven resistance.

Distinct phenotypic states are associated with specific metabolic programs, highlighting metabolism as a therapeutic vulnerability (8, 9). S-palmitoylation, a reversible posttranslational modification in which palmitic acid is added to cysteine residues, regulates protein localization, stability, and function (10–12). This reaction is catalyzed by Protein S-Acyl Transferases (PATs), a family of 24 ZDHHC enzymes. Although palmitoylation is dynamic and potentially druggable, its role in cancer remain poorly defined.

Melanoma provides a strong model to study these processes (13). Although early stage disease is highly treatable, metastatic melanoma has poor survival, and around 45% of patients fail to respond to immunotherapy (14). These observations have sparked interest in whether small molecules can not only limit metastatic outgrowth but also reshape the intratumor immune landscape. Achieving this, however, requires identifying the pathways that govern the bidirectional interactions between cancer cells and the immune system.

Here, we investigate whether PATs regulate melanoma progression and immunity. We identify ZDHHC13-mediated palmitoylation of CTNND1 as a suppressor of metastasis. We further show that ZDHHC13 reshapes the immune microenvironment by altering melanoma lipid metabolism, reducing M2-like tumor-associated macrophages and slowing tumor growth in immunocompetent mice.

## Results

### CTNND1 is a potential palmitoylated protein responsible for melanoma metastasis.

**Figure 1:**
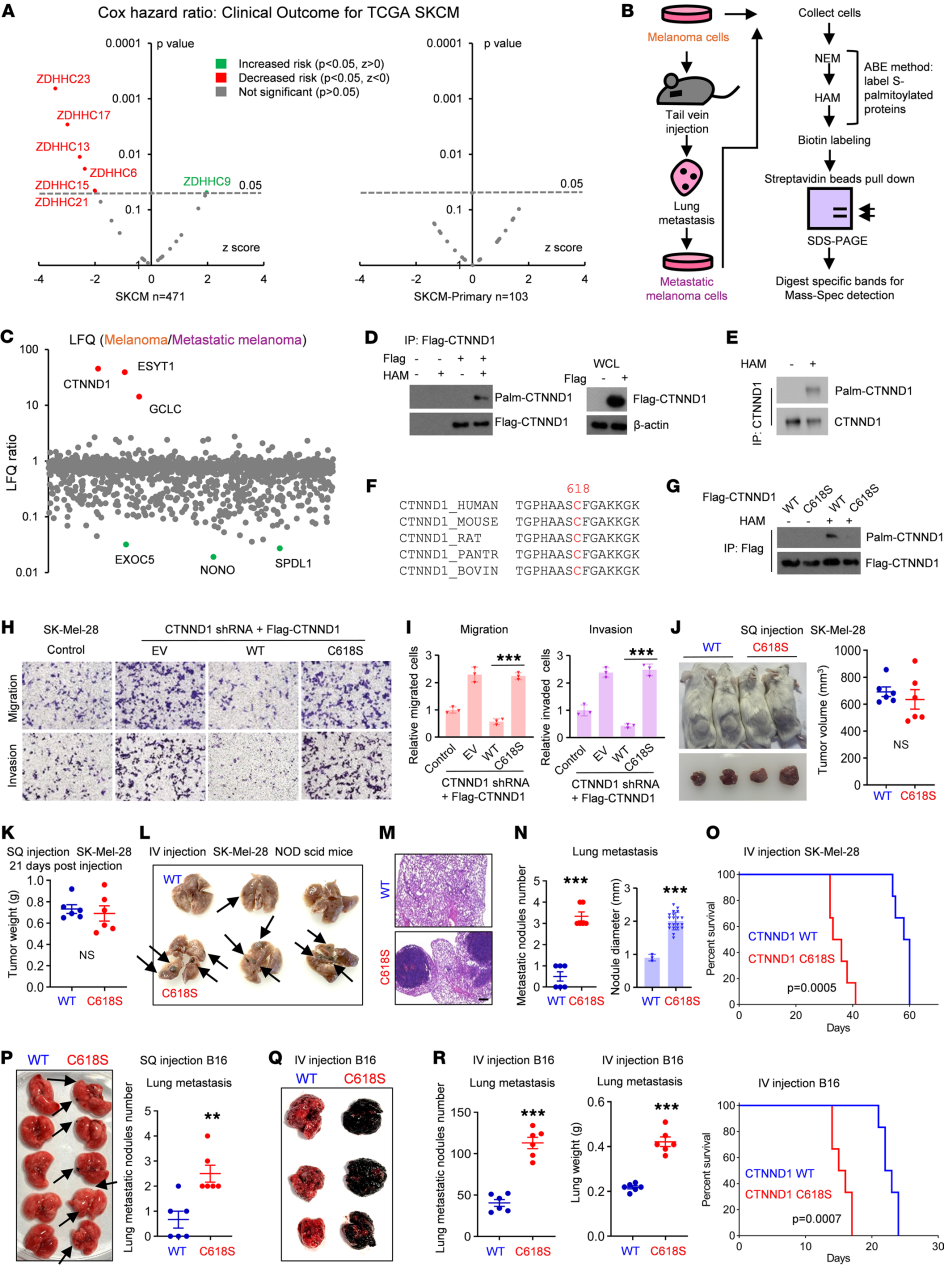
CTNND1 palmitoylation delays melanoma metastasis to the lung. (**A**) Cox proportional hazards model of PATs, calculated by TIMER2.0 with adjustment for age, gender, and race. (**B**) The scheme of in vivo screening for palmitoylating proteins essential for metastasis. (**C**) Mass spectrometry LFQ showing CTNND1 enrichment in SK-Mel-28 versus lung metastases. (**D** and **E**) Palmitoylation of exogenous (**D**) and endogenous (**E**) CTNND1 detected by ABE assay. (**F**) Sequence alignment of CTNND1 showing conserved palmitoylation motif. (**G**) CTNND1 palmitoylation at C618 confirmed by ABE. (**H** and **I**) Migration (**H**) and invasion (**I**) assays in SK-Mel-28 cells stably expressing WT or C618S CTNND1. (**J** and **K**) 1 × 10^6^ SK-Mel-28 cells stably expressing WT or C618S CTNND1 were subcutaneously injected into the flank of NOD scid mice. Tumor volume (**J**) and weight (**K**) were measured 21 days after tumor cell injection (*n* = 6). (**L**–**O**) Lung metastasis after tail vein injection of 5 × 10^5^ SK-Mel-28 cells expressing WT or C618S CTNND1 (*n* = 6): lungs (**L**), H&E staining (**M**), quantification (**N**), and survival (**O**). Scale bars: 185 μm (**M**). Original magnification, ×200 (**H**). (**P**) 1 × 10^6^ B16 stably expressing WT or C618S CTNND1 in 100 μl PBS were subcutaneously injected into the flank of NOD scid mice. Pulmonary metastases were measured 12 days after tumor cell injection (*n* = 5). (**Q**–**T**) 2 × 10^5^ B16 stably expressing WT or C618S CTNND1 in 100 μl PBS were injected into NOD scid mice via the tail vein. Pulmonary metastases (**Q**–**S**) were assessed 14 days after tumor cell injection and mice survival were recorded (**T**). All data in this Figure are mean ± SD. *P < 0.05, **P < 0.01, ***P < 0.001, unpaired student’s *t* test.

Protein S-palmitoylation’s role in human diseases remains underexplored. In this study, we employed a Cox proportional hazard model to analyze the prognostic significance of 24 ZDHHC protein acyltransferases in melanoma samples from The Cancer Genome Atlas (TCGA). Our findings revealed that approximately 25% of these ZDHHCs are associated with improved survival outcomes in melanoma patients (Figure 1A), suggesting a potential protective effect. Notably, these effects were absent in primary melanoma cases, highlighting a distinct role for palmitoylation in the progression of metastatic melanoma (Figure 1A).

To further delineate the specific proteins undergoing palmitoylation that may contribute to melanoma metastasis, we developed an enhanced metastatic melanoma cell line, SK-Mel-28M, through the intravenous injection of SK-Mel-28 cells into mice and subsequent collection of lung metastases (Figure 1B and Supplemental Figure 1A; supplemental material available online with this article; https://doi.org/10.1172/JCI188249DS1). Utilizing the acyl-biotin exchange (ABE) technique, we systematically identified palmitoylated proteins in both SK-Mel-28 and SK-Mel-28M cell lysates. Mass spectrometry analysis highlighted a differential enrichment of palmitoylated proteins in the original cell line compared with its metastatic counterpart, with Catenin Delta 1 (CTNND1) emerging as a protein of interest (Figure 1C and Supplemental Figure 1B).

CTNND1, an armadillo (ARM) repeat-containing protein, is integral to the formation of adherens junctions through its interactions with Cadherin proteins. Immunofluorescence revealed CTNND1’s presence both on the cell membrane and in the cytosol of melanoma cell lines (Supplemental Figure 1C). Subsequent validation of CTNND1 palmitoylation using the ABE method (Figure 1, D–E, and Supplemental Figure 1D) and analysis against the SwissPalm database suggested C394 and C618 as candidate palmitoylation sites (Figure 1F and Supplemental Figure 1E). Mutation analysis demonstrated a significant reduction in palmitoylation of Flag-tagged CTNND1 upon mutation of C618, but not C394 (Figure 1G and Supplemental Figure 1, F–H), establishing it as the primary palmitoylation site. Our data underscores the palmitoylation of CTNND1 as a critical posttranslational modification in melanoma, with implications for understanding the molecular mechanisms underlying melanoma metastasis.

### CTNND1 palmitoylation delays melanoma metastasis to the lung.

To investigate the impact of CTNND1 palmitoylation on melanoma metastasis, we generated melanoma cell lines expressing either WT CTNND1 or the palmitoylation-deficient C618S mutant (Supplemental Figure 2, A and B). In vitro proliferation assays showed that CTNND1 palmitoylation had no significant effect on melanoma cell growth (Supplemental Figure 2C). However, transwell migration and invasion assays revealed that the C618S mutation significantly enhanced melanoma cell migration and invasion compared with WT cells (Figure 1, H and I, and Supplemental Figure 2D).

To extend these findings in vivo, we used 2 murine models. In the first model, SK-Mel-28 cells were injected subcutaneously into the flanks of immunodeficient mice. No significant differences were observed in tumor growth between WT or C618S mutant CTNND1 (Figure 1, J and K, and Supplemental Figure 2E). However, this model did not result in observable metastasis, likely due to the rapid primary tumor growth outpacing early metastatic events, such as local invasion and intravasation. To better assess the metastasis, we used the tail vein injection method, and the result showed that C618S mutation significantly promoted lung colonization and reduced overall survival (Figure 1, L–O).

We replicated these experiments using B16 mouse melanoma cells. Similar to the SK-Mel-28 model, mice with subcutaneous inoculated B16 cells showed no significant differences in tumor growth between WT and C618S mutant CTNND1 groups (Supplemental Figure 2, F and G). Although this model displayed minimal lung metastasis, the C618S mutation markedly increased the number of lung metastatic nodules (Figure 1P). Tail vein injection of B16 cells further confirmed that the C618S mutation significantly enhanced lung colonization and reduced survival (Figure 1, Q–T). These results highlight the critical role of CTNND1 palmitoylation in modulating melanoma metastasis to the lung.

### ZDHHC13 is the major PAT of CTNND1.

**Figure 2:**
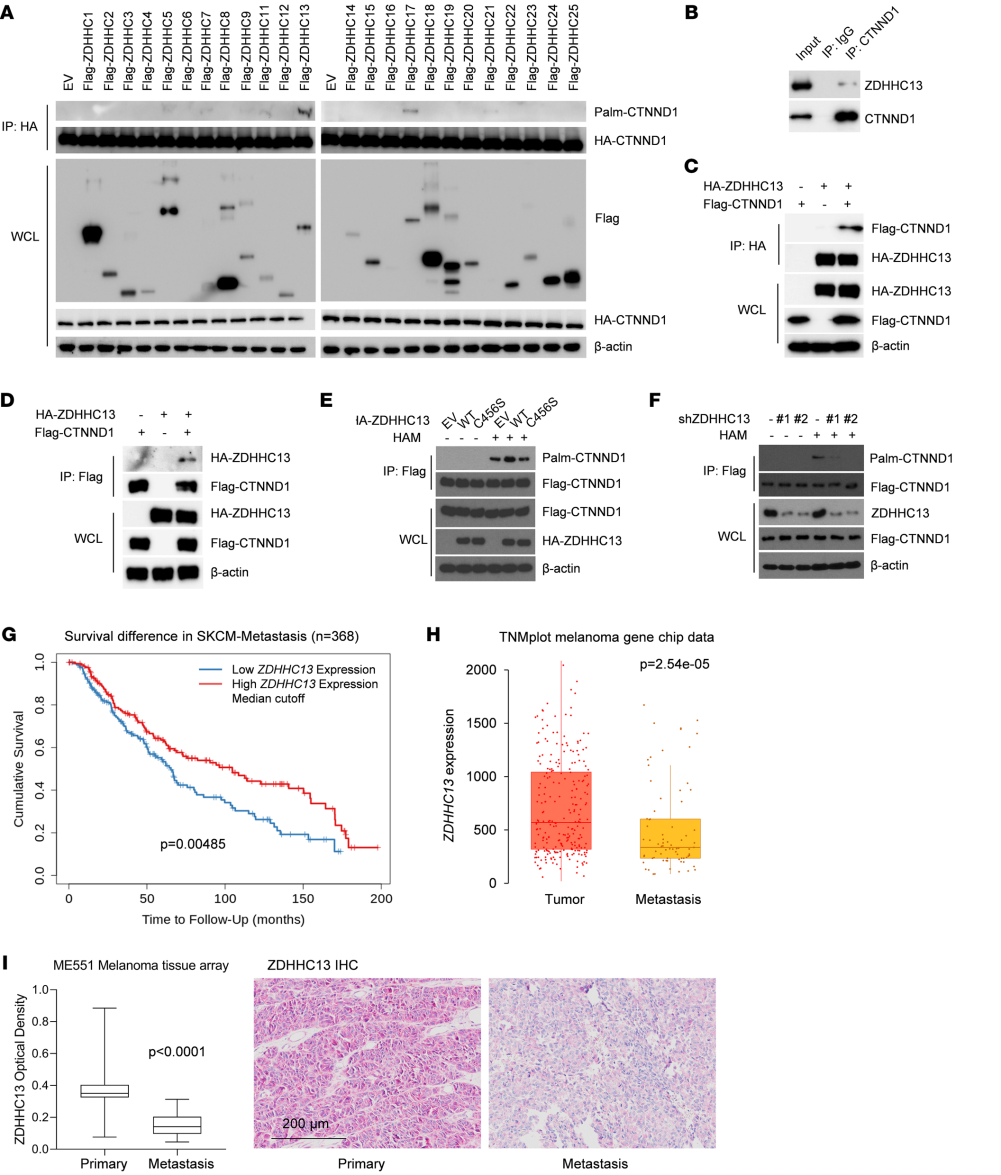
ZDHHC13 acts as the primary PAT for CTNND1 and plays a tumor-suppressive role in human metastatic melanoma samples. (**A**) HEK293 cells were cotransfected with HA-CTNND1 and Flag-Myc-Zdhhcs in 6-well plate. Cell lysates were harvested for IP, ABE, and Immunoblot (IB) analysis. (**B**) IB analysis of whole-cell lysate (WCL) and anti-CTNND1 immunoprecipitates from SK-Mel-28 cells. (**C** and **D**) IB analysis of WCL, anti-Flag or anti-HA immunoprecipitates from HEK293T cells transfected with HA-ZDHHC13 and Flag-CTNND1 constructs. (**E** and **F**) SK-Mel-28 cells expressing Flag-CTNND1 and HA-ZDHHC13 (**E**) or Flag-CTNND1 with shZDHHC13 (**F**), analyzed by IP, ABE, and IB. (**G**) Prognosis analysis for TCGA SKCM-metastasis patients (timer.cistrome.org). Samples were divided into ZDHHC13-high and -low expression groups by the median (50%). (**H**) ZDHHC13 expression in melanoma versus metastatic melanoma (TNMplot dataset). (**I**) IHC of ZDHHC13 in melanoma tissue array (27 primary, 22 metastatic). Staining quantified in ImageJ. Scale bar: 200 μm. Differences assessed with unpaired 2-tailed Student’s *t* test.

To identify the PATs implicated in the palmitoylation of CTNND1, we coexpressed HA-tagged CTNND1 with 24 Myc-Flag-tagged mouse ZDHHC PATs. Palmitoylation of CTNND1 was notably observed with the coexpression of ZDHHC13 and 17 (Figure 2A), with ZDHHC13 showing the most pronounced effect on enhancing CTNND1 palmitoylation (Figure 2A). Further validation in melanoma cells confirmed that ZDHHC13 is the primary PAT responsible for CTNND1 palmitoylation (Supplemental Figure 3A). The interaction between ZDHHC13 and CTNND1 was further validated through coimmunoprecipitation experiments in SK-Mel-28 melanoma cells and HEK-293T cells (Figure 2, B–D). Investigating ZDHHC13’s functionality, we introduced a C456S mutation within the enzyme’s DQHC motif, crucial for its catalytic activity. This mutation significantly compromised CTNND1 palmitoylation (Figure 2E and Supplemental Figure 3B). Silencing ZDHHC13 expression led to a marked decrease in CTNND1 palmitoylation (Figure 2F and Supplemental Figure 3, C and D), while its overexpression enhanced the palmitoylation of CTNND1 (Figure 2E). Additionally, we observed a marked reduction in ZDHHC13 mRNA and protein levels in the metastatic SK-Mel-28M cell line compared with the parental line (Supplemental Figure 3E), suggesting that cells with lower ZDHHC13 expression may have been selectively enriched during metastasis in our model. These results underscore ZDHHC13’s pivotal role in CTNND1 palmitoylation, indicating a direct regulatory mechanism by ZDHHC13 in this posttranslational modification process.

To investigate the impact of ZDHHC13 on human melanoma, we commenced our study by analyzing data from the TCGA metastatic melanoma cohort. Clinical data analysis revealed a notable association between increased *ZDHHC13* mRNA expression and enhanced survival rates in patients with metastatic melanoma (Figure 2G). *ZDHHC13* expression was significantly reduced in human melanoma samples compared with normal tissues across multiple cohorts (Supplemental Figure 3, F–H), and ZDHHC13 expression levels were even lower in metastatic melanoma samples (Figure 2H). However, expression levels of *ZDHHC17* and *ZDHHC21* were not significantly associated with patient survival (Supplemental Figure 4A). In contrast, our results showed that ZDHHC13 overexpression significantly inhibited melanoma cell migration and invasion, while *ZDHHC13* knockdown markedly enhanced these behaviors (Supplemental Figure 4, B and C). Silencing *ZDHHC17* or *ZDHHC21*, however, had no significant impact on CTNND1 palmitoylation or melanoma cell invasion (Supplemental Figure 4, B and C). Furthermore, we conducted additional analysis using a human melanoma tissue array (ME551), comprising 27 primary and 22 metastatic melanoma samples. IHC staining revealed that ZDHHC13 protein levels were significantly lower in metastatic melanoma (Figure 2I). These data suggest a potential tumor-suppressive role for ZDHHC13 in melanoma.

### Palmitoylation is essential for the interaction between CTNND1 and E-cadherin.

**Figure 3:**
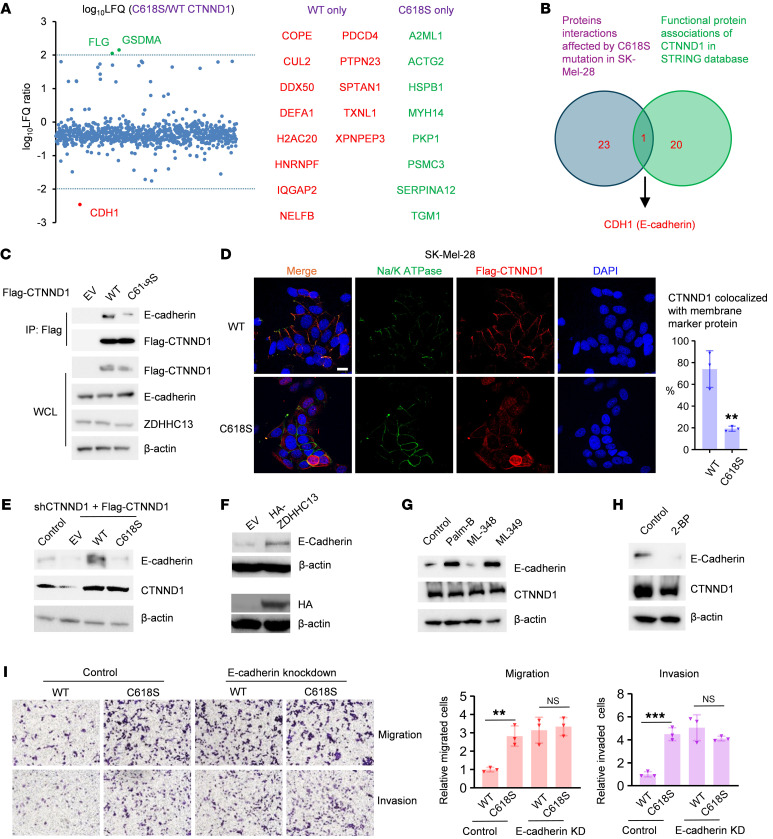
Palmitoylation is essential for the interaction between CTNND1 and E-cadherin. (**A**) Mass spectrometry of Flag-CTNND1 complexes from SK-Mel-28 cells expressing WT or C618S CTNND1. (**B**) E-cadherin identified as a major palmitoylated CTNND1 binding partner. (**C**) Coimmunoprecipitation of E-cadherin with WT or C618S CTNND1 in SK-Mel-28 cells after proteasome and lysosome inhibition (5 μM MG132 and 50 μM Chloroquine for 6 hours). (**D**) Confocal imaging of CTNND1 and Na/K ATPase in SK-Mel-28 cells expressing WT or C618S CTNND1. Scale bar: 25 μm. (**E**) C618S CTNND1 failed to stabilize E-cadherin following *CTNND1* knockdown and rescue in SK-Mel-28 cells. (**F**–**H**) IB analysis of E-cadherin stability in SK-Mel-28 cells after ZDHHC13 overexpression (**F**) or treatment with: (**G**) 1 μM Palm-B, ML348, ML349 or DMSO vehicle control for 24 hours; (**H**) 10 μM 2-BP or DMSO vehicle control for 24 hours. (**I**) Migration and invasion of SK-Mel-28 cells expressing WT or C618S CTNND1, with or without E-cadherin knockdown. Original magnification, ×200. Data are mean ± SD (*n* = 3). *P < 0.05, **P < 0.01, ***P < 0.001, unpaired Student’s *t* test.

To explore the mechanism of CTNND1 palmitoylation on melanoma metastasis, we conducted mass spectrometry screening for proteins interacting with WT and C618S CTNND1 in melanoma cells. The screening identified 24 partner proteins notably influenced by the C618 mutation (Figure 3A). Intersection with the STRING database’s functional protein associations for CTNND1 (Supplemental Figure 5A) highlighted E-cadherin as a potential impacted functional partner by CTNND1 palmitoylation (Figure 3B). Immunoprecipitation (IP) assays verified that the C618S mutation substantially diminishes the CTNND1–E-cadherin interaction (Figure 3C). CTNND1 palmitoylation did not appear to influence its interaction with RhoA (Supplemental Figure 5B), another known binding partner of CTNND1.

Given the role of S-palmitoylation in directing proteins to cellular membranes, our research sought to ascertain whether S-palmitoylation affects the membrane association of CTNND1. Immunofluorescence indicated that WT CTNND1 localized to both the plasma membrane and the cytoplasm, while the CTNND1 (C618S) mutant was predominantly cytoplasmic (Figure 3D). Additionally, membrane colocalization of CTNND1 with E-cadherin was observed only with the WT CTNND1, but substantially reduced with the C618S mutant (Supplemental Figure 5C), suggesting that palmitoylation is essential for CTNND1’s membrane association with E-cadherin. Our results demonstrate that, in the parental SK-Mel-28 cells, CTNND1 is distributed between the membrane and cytosol. In contrast, in the metastatic SK-Mel-28 variant, CTNND1 is predominantly localized in the cytosolic fraction, indicating a loss of membrane-associated CTNND1 (Supplemental Figure 6A). Notably, our data revealed that membrane-localized CTNND1 is highly enriched for palmitoylation — approximately 10-fold higher than in the cytosolic fraction (Supplemental Figure 6B). Moreover, comparison between the parental and metastatic cell lines showed a marked reduction in both total and palmitoylated CTNND1 at the membrane in the metastatic cells, suggesting a potential association between reduced membrane-associated, palmitoylated CTNND1 and enhanced metastatic potential.

Furthermore, we observed a significant reduction in E-cadherin protein levels associated with the C618S mutation (Figure 3E), while overexpression of ZDHHC13 led to an increase in E-cadherin levels (Figure 3F). Treatment with APT1/2 depalmitoylating enzyme inhibitor Palm-B and the APT2 inhibitor ML349 markedly enhanced CTNND1 palmitoylation (Supplemental Figure 6C) and increased E-cadherin protein levels (Figure 3G), unlike ML348 (APT1 inhibitor). Conversely, inhibition of palmitoylation through 2-BP treatment resulted in decreased E-cadherin levels (Figure 3I). Moreover, ML349 treatment suppressed melanoma cell migration and invasion in vitro (Supplemental Figure 6D). However, the role of E-cadherin in cancer progression remains controversial (15–23). For instance, E-cadherin has been shown to promote metastasis in certain breast cancer models (15). Our clinical analyses revealed tumor-specific expression patterns: E-cadherin is upregulated in breast cancer samples but significantly downregulated in melanoma samples, suggesting context-dependent roles (Supplemental Figure 7A). IHC analysis of a human melanoma tissue array (ME551) further confirmed reduced E-cadherin expression in metastatic melanoma (Supplemental Figure 7B). Moreover, overexpression of E-cadherin significantly suppressed lung metastasis in the mouse model (Supplemental Figure 7C), further underscoring its critical antimetastatic role in melanoma. E-cadherin knockdown in cells expressing WT CTNND1 also abrogated CTNND1’s ability to inhibit melanoma migration and invasion (Figure 3H), indicating that CTNND1 palmitoylation mainly suppresses melanoma metastasis through E-cadherin in vitro. Our findings demonstrate that S-palmitoylation is critical for CTNND1’s recruitment to the membrane and its interaction with E-cadherin, suggesting that CTNND1 palmitoylation regulates melanoma metastasis by modulating E-cadherin.

### ZDHHC13 suppresses melanoma growth and metastasis in immunocompetent mice.

Next, we aimed to assess whether ZDHHC13 could be a viable target for suppressing melanoma metastasis. We explored whether ZDHHC13 suppresses metastasis primarily through CTNND1 palmitoylation. To test this, we overexpressed ZDHHC13 in melanoma cells expressing either WT or C618S CTNND1 and conducted in vitro migration and invasion assays (Supplemental Figure 8, A and B). The results revealed that ZDHHC13 effectively suppressed metastasis in cells with WT CTNND1, but not in those with the C618S mutation (Supplemental Figure 8, A and B), suggesting that ZDHHC13 suppresses melanoma metastasis primarily through CTNND1 palmitoylation in vitro.

**Figure 4:**
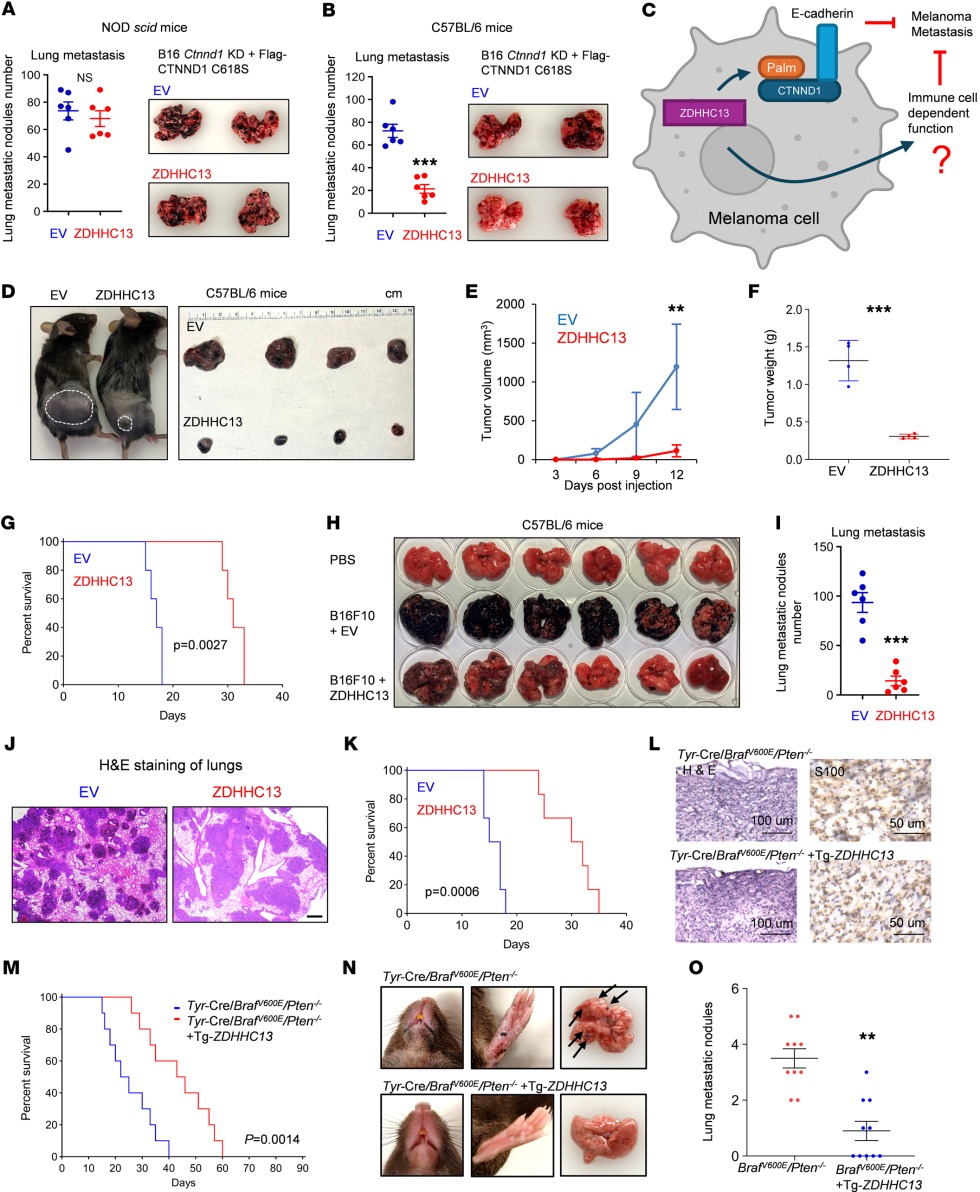
ZDHHC13 suppress melanoma growth and metastasis in immunocompetent mouse. (**A** and **B**) 2 × 10^5^ Ctnnd1-depleted B16 stably expressing C618S CTNND1 and ZDHHC13 in 100 μl PBS were injected into NOD scid mice (**A**) or C57BL/6 mice (**B**) via the tail vein. Pulmonary metastases were assessed 14 days after tumor cell injection (*n* = 6). (**C**) Schematic model: ZDHHC13-mediated palmitoylation of CTNND1 suppresses melanoma progression by stabilizing E-cadherin and modulating immune cell function. (**D**–**G**) 1 × 10^6^ B16 or B16 stably expressing ZDHHC13 in 100 μl PBS were subcutaneously injected into the shaved flank of C57BL/6 mice. Tumor growth, weight (*n* = 4) and mouse survival (*n* = 5) were assessed. (**H**–**K**) 2 × 10^5^ B16 or B16 stably expressing ZDHHC13 in 100 μl PBS were injected into C57BL/6 mice via the tail vein. Pulmonary metastases (**H** and **I**) and lung H&E staining (**J**) were assessed 14 days after tumor cell injection. Mouse survival was recorded (**K**) (*n* = 6). Scale bar: 185 μm. (**L**–**M**) Representative locally induced melanoma sections and survival curve and of indicated mice. Scale bar: 100 μm. (**N**) Tumor metastasis from a *Tyr*-re/*Braf^V600E^/Pten^–/–^* mouse (day 40 after Tam injection) and a *Tyr*-Cre/*Braf^V600E^/Pten^–/–^* +Tg*-ZDHHC13* mouse (day 55 after Tam injection). (**O**) Comparison of number of lung metastases visible on the surface of the lungs in indicated mice. All data in this Figure are mean ± SD. *P < 0.05, **P < 0.01, ***P < 0.001, unpaired student’s *t* test.

Significantly, ZDHHC13 failed to suppress lung colonization in immunodeficient mice with melanomas expressing C618S CTNND1 (Figure 4A), but was able to do so in immunocompetent C57BL/6J mice with the same C618S CTNND1–expressing tumors (Figure 4B). These findings suggest that ZDHHC13 may have additional immune-dependent, but CTNND1-independent, roles in suppressing melanoma pulmonary metastatic outgrowth (Figure 4C). Given that the tumor immune microenvironment influences many aspects of cancer behavior, we cannot exclude the possibility that ZDHHC13 affects multiple stages of the metastatic cascade, including tumor cell delamination, invasion, survival, and subsequent proliferation at the metastatic site.

To confirm ZDHHC13’s potential tumor-suppressive role in melanoma within an immunocompetent setting, we engineered B16 cells to overexpress ZDHHC13 via lentiviral transduction (Supplemental Figure 8C). When these cells were inoculated into immune-deficient NOD SCID mice, ZDHHC13 overexpression had no effect on tumor growth or mass (Supplemental Figure 8, D–F), although it continued to suppress lung metastasis (Supplemental Figure 8, G and H). In contrast, in immunocompetent C57BL/6J mice, ZDHHC13 overexpression significantly suppressed both subcutaneous tumor growth and pulmonary metastatic colonization following tail vein injection (Figure 4, D–K, and Supplemental Figure 8I).

Additionally, we explored melanoma development using a genetically engineered mouse model (Supplemental Figure 8J). We crossed our previously generated Tg-*Tyr-ZDHHC13* mice (11) with a melanoma mouse model *Tyr*-Cre-*Braf^V600E^/Pten^–/–^*. Upon tamoxifen-induced activation of *Tyr*-Cre-*Braf^V600E^/Pten^–/–^*, all mice developed melanomas with tumors displaying consistent morphological and histological characteristics (Figure 4L). Notably, mice with transgenic expression of ZDHHC13 exhibited significantly improved survival rates (Figure 4M) and delayed tumor growth (Supplemental Figure 8, K and L). Our data also indicated that transgenic ZDHHC13 expression inhibited melanoma metastasis to the lungs (Figure 4, N and O). These results further support the tumor-suppressive role of ZDHHC13 in melanoma, suggesting that this effect partially relies on an intact immune response.

### ZDHHC13 regulates lipid metabolism in melanomas.

**Figure 5:**
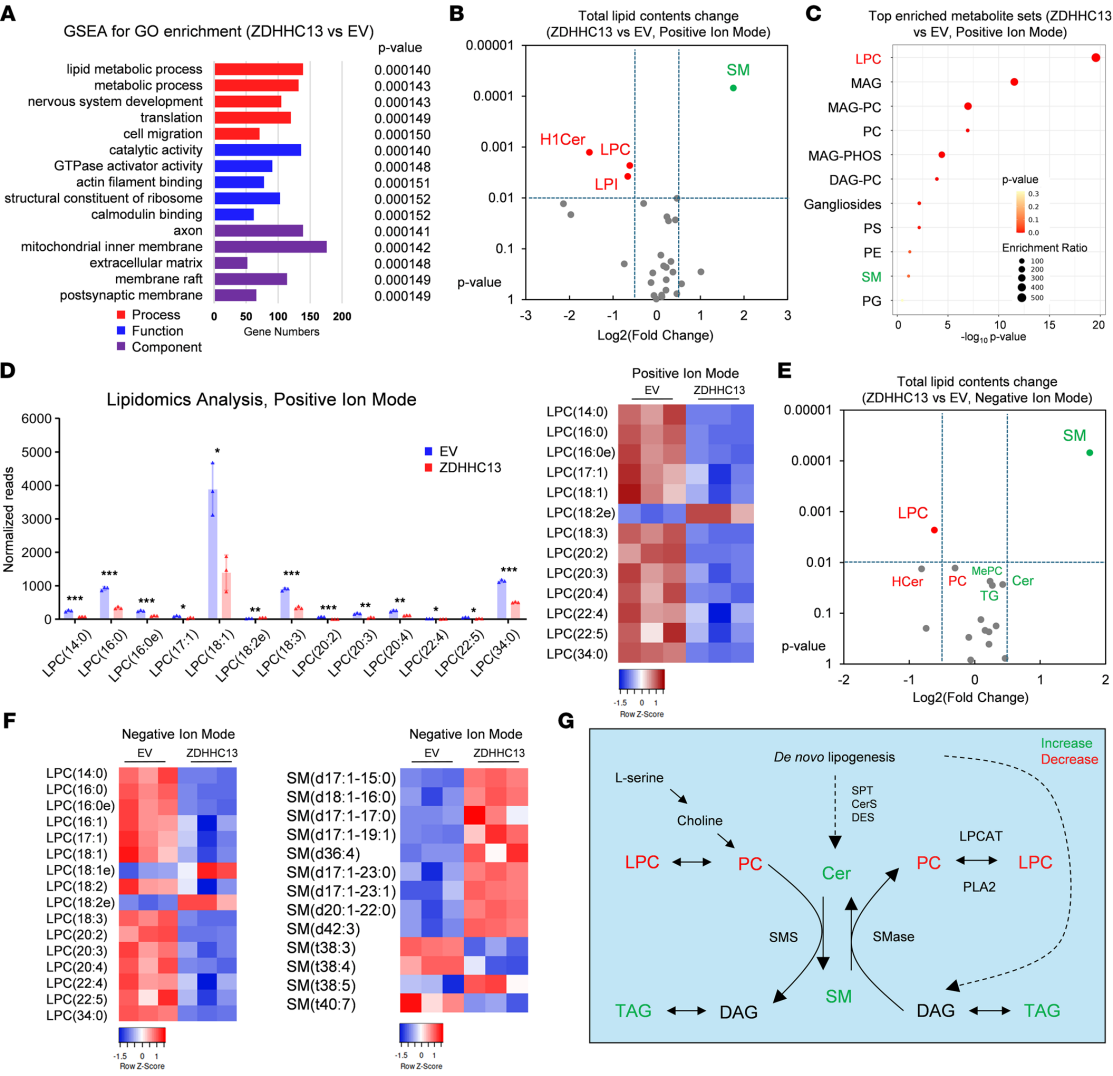
ZDHHC13 regulates lipid metabolism in melanomas. (**A**) RNA-seq analysis of B16 cells with or without ZDHHC13 expression, including differential gene expression and GO enrichment by GSEA (tool: RaNA-seq). (**B**) The change of lipid species in B16 stably expressing ZDHHC13 vs B16 expressing control empty vectors (Positive Ion Mode). (**C**) Pathway enrichment analysis of lipids based on KEGG database and MetaboAnalyst. Lipid metabolites with a variable importance in the projection (VIP) score > 1, Fold Change > 2.0 and *P* < 0.05 were considered significant changed lipids (Positive Ion Mode). (**D**) Quantification of LPC species. (**E** and **F**) Lipidomic profiling and heatmap of LPC and SM species in negative ion mode. (**G**) LPC and SM metabolic pathway.

To elucidate how ZDHHC13 enhances the immune response within the TME, we conducted RNA-seq analysis (Figure 5A and Supplemental Figure 9, A and B). By analyzing gene expression changes associated with ZDHHC13 overexpression, we identified lipid metabolism as the most significantly affected molecular pathway. Given the pivotal role of lipid metabolism in shaping immune responses, this discovery prompted us to conduct an untargeted lipidomics analysis on B16 cells overexpressing ZDHHC13 (Supplemental Figure 9, C–E) to delineate the specific lipid profiles altered by ZDHHC13 and provide insights into how these changes could influence immune cell functionality and interactions within the TME.

Lipidomics utilizes mass spectrometry in both positive and negative ion modes to comprehensively analyze lipids. Positive ion mode detects lipids that form stable cations, such as phosphatidylcholines (PC), sphingomyelins (SM), and other neutral lipids, while negative ion mode is better suited for lipids that readily form anions, like phosphatidylinositols (PI), phosphatidylserines (PS), phosphatidylethanolamines (PE), phosphatidylglycerols (PG), phosphatidic acids (PA), and other anionic lipids. Using positive ion mode, we observed significant reductions in monohexosylceramides (Hex1Cer), lysophosphatidylcholine (LPC), and lysophosphatidylinositols (LPI), with SM showing the most notable increase (Figure 5B). Metabolite set enrichment analysis identified the LPC metabolism pathway as the most significantly affected (Figure 5C). Notably, LPC 18:1 emerged as the most abundant LPC species in B16 melanoma cells (Figure 5D). In negative ion mode, LPC also showed the most significant reduction, while SM again displayed the most significant increase (Figure 5, E and F). The LPC metabolism pathway was also significantly changed in the negative ion mode dataset (Supplemental Figure 9F). An increase of triacylglycerols (TG) and reduction of PC was also observed (Supplemental Figure 9G). These findings imply that LPC might function as a “protumor” lipid within the melanoma tumor TME.

### ZDHHC13 reshapes the tumor immune microenvironment.

**Figure 6:**
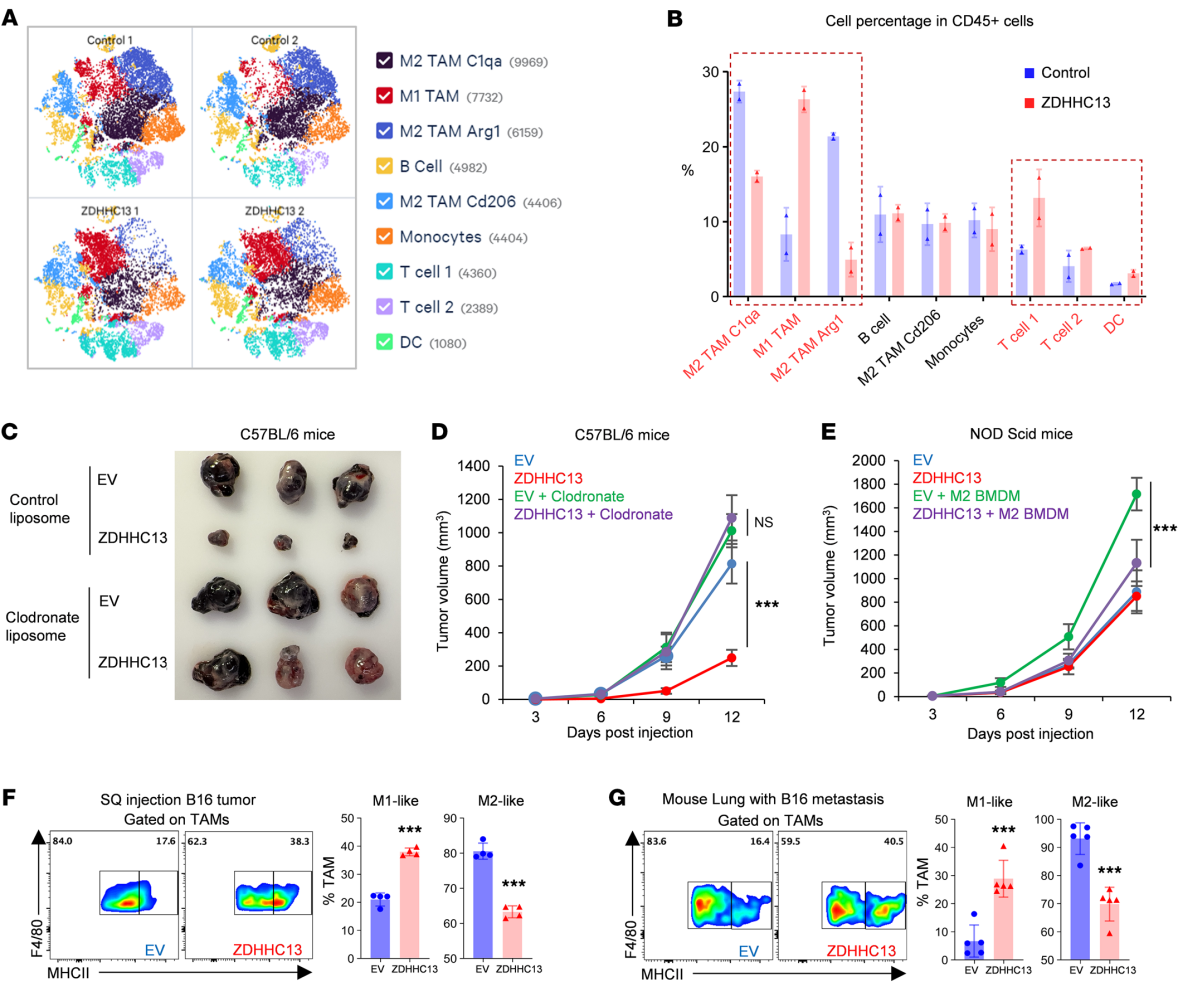
ZDHHC13 reshapes the tumor immune microenvironment. (**A**) 1 × 10^6^ B16 or B16 stably expressing ZDHHC13 in 100 μl PBS were subcutaneously injected into the shaved flank of C57/BL6 mice. The t-SNE plot visualization of 10 × single cell transcriptome of 45,481 CD45^+^ cells sorted from tumors on day 12 was generated by Loupe. Color coded clusters were generated by K-means clustering. Cluster identity was assigned based on the expressions of lineage specific markers. (**B**) Distribution of cells from each genotype across clusters. (**C** and **D**) Macrophages were depleted in C57BL/6 mice through IV injection of clodronate liposomes, given 2 days before tumor inoculation. Three injections will be administered with 3-day intervals between each dose to maintain macrophage depletion. Mice were injected with 1 × 10^6^ B16 melanoma cells, tumor growth were monitored. (**E**) NOD scid mice were subcutaneously inoculated with 5 × 10^5^ B16F10 in 100 μl PBS mixed with 2 × 10^5^ Bone marrow-derived macrophages (BMDMs). BMDMs were pretreated with IL-4, TGF-β and IL-10 mix (10 ng/ml) for 24 hours to induced M2-like polarization. Tumor growth was recorded. (**F**) Flow cytometry of tumor-infiltrating macrophages showing M1- and M2-like subsets from tumors in Figure 4D (*n* = 4). (**G**) Flow cytometry of lung-infiltrating macrophages showing M1- and M2-like subsets from metastases in Figure 4H (*n* = 5). All data in this Figure are mean ± SD. *P < 0.05, **P < 0.01, ***P < 0.001, unpaired student’s *t* test.

Given the known role of LPC in modulating immune responses via the G protein–coupled receptor G2A (*GPR132*) and that G2A is highly expressed in macrophage and lymphocytes (24), we next examined immune cell populations in the tumor microenvironment using scRNA-seq. The analysis identified the most prominent and significantly altered cell clusters as Tumor-associated macrophages (TAMs) (Clusters 1, 2, and 3), characterized using macrophage markers such as *Adgre1* (*F4/80*), *Apoe*, *Lyz2*, *Ccr2*, and *Ccl2*. Notably, TAM Cluster 2 exhibited enrichment for genes associated with antigen presentation (e.g., *Cd74*, *H2-Ab1*, *H2-Aa*, *H2-Eb1*), indicative of a potent classically activated M1-like phenotype. Conversely, Clusters 1 and 3 were classified as M2-like TAM subpopulations, evidenced by their expression of phagocytosis-associated complement family members (e.g., *C1qa*, *C1qb*, *C1qc*) and genes linked to anti-inflammatory and oncogenic processes (e.g., *Arg1*, *Spp1*, *Vegfa*), respectively (Figure 6, A and B, and Supplemental Figure 10A). Pathway analysis in TAMs across Clusters 1, 2, and 3 revealed that ZDHHC13 overexpression in tumors led to upregulation of several pathways associated with MHC II function, which represent a strong M1-like phenotype (Supplemental Figure 10B). Additionally, increases in T cell and dendritic cell (DC) populations were also observed (Figure 6B). Using single gene expression feature, further exploration revealed that ZDHHC13 overexpression decreased the proportion of cells enriched for the M2 TAM markers *Arg1* and *Spp1*, and also increased the proportion of cells enriched in *Cd4*, *Cd8a*, *Gzmb*, and *Ncr1* (NK cell marker) expression (Supplemental Figure 10, C and D).

Because G2A is highly expressed in macrophages and lymphocytes (24), and our scRNA-seq data indicate that both macrophages and T lymphocytes are the most substantially altered immune populations in response to ZDHHC13 overexpression (Figure 6B), we investigated which cell type serves as the primary target of the ZDHHC13–LPC–G2A axis. To address this, we first depleted macrophages in C57BL/6J mice using clodronate liposomes (Supplemental Figure 11A). Macrophage depletion abolished the tumor-suppressive effect of ZDHHC13, indicating that macrophages are essential mediators of this response (Figure 6, C and D). We next assessed whether ZDHHC13 directly affects macrophage function. Although NOD scid mice retain a limited population of functionally impaired macrophages, comparison of tumor-infiltrating macrophages in B16 tumors revealed that NOD scid mice harbored approximately 6-fold fewer macrophages than immunocompetent C57BL/6J mice (Supplemental Figure 11, B and C). Moreover, the macrophages present in NOD scid mice failed to exhibit polarization toward a protumorigenic phenotype (Supplemental Figure 11, D and E). These findings suggest that NOD scid mice may not be an appropriate model for studying the function of protumor macrophages.

To directly assess whether ZDHHC13 affects macrophage function, we coinjected B16 cells with bone marrow–derived, M2-polarized macrophages. In this model, ZDHHC13 overexpression significantly suppressed tumor growth (Figure 6E), supporting the hypothesis that ZDHHC13’s antitumor activity is mediated through modulation of protumor macrophages.

Of particular interest, in the M2 TAM Arg1 cell cluster — which showed the most substantial decrease due to ZDHHC13 overexpression — a significant enrichment of protumor M2-like TAM markers, including *Arg1*, *Spp1*, *Vegfa*, and *Cd274* were noted (Supplemental Figure 11F). Analysis also indicated ZDHHC13 induces a reduction in Arg1 and Spp1 in TAMs (Supplemental Figure 11G). Utilizing the xCell quantification algorithm, we observed a significant relationship between elevated *ZDHHC13* mRNA levels, reduced M2 macrophage infiltration, and increased presence of Memory CD4^+^ T cells in TCGA human metastatic melanoma samples (Supplemental Figure 12, A and B). These results demonstrated that ZDHHC13 may enhance the antitumor immune response by inhibiting the function of “protumor” M2-like TAMs.

To confirm ZDHHC13’s influence on immune cell infiltration, we analyzed B16 tumor-infiltrating immune cells using flow cytometry. We employed macrophage lineage markers (CD11b^+^Ly6G^–^Ly6C^–^Siglec-F^–^F4/80^+^) and MHC class II to distinguish between M1-like (MHC class II^High^) and M2-like (MHC class II^low^) TAMs. Our results indicated that ZDHHC13 in B16 cells notably increased the proportion of M1-like TAMs (Figure 6, F and G), CD8^+^ (Supplemental Figure 12C), CD4^+^ (Supplemental Figure 12D), and NK cells (Supplemental Figure 12E), while diminishing the population of M2-like TAMs in subcutaneously inoculated tumors as well as in tail vain injection–induced melanoma lung metastasis (Figure 6, F and G). We also applied an alternative method (F4/80, Arg1) to confirm the reduction of M2-like TAMs in the TME (Supplemental Figure 12F). Additionally, in the genetically engineered mouse model *Tyr*-Cre-*Braf^V600E^/Pten^–/–^*, we observed a significant reduction in LPC levels within Tg-*ZDHHC13* melanomas (Supplemental Figure 13A), consistent with our central hypothesis. Flow cytometry analysis of these tumors revealed decreased infiltration of M2-like TAMs and MMP12^+^ macrophages, alongside increased infiltration of M1-like TAMs and CD8^+^ T cells (Supplemental Figure 13, B–D). These results support our conclusion that ZDHHC13 enhances the antitumor immune response majorly by reducing M2-like TAMs in the TME.

E-cadherin is the only known ligand of CD103 (Integrin αE, *Itgae*) (25, 26), a receptor expressed on immune cells. Given that ZDHHC13 increases E-cadherin protein in melanoma cell culture (Figure 3F) and in vivo (Supplemental Figure 8, I and L), we investigated whether ZDHHC13 could also increase the infiltration of CD103^+^ immune cells into the melanoma microenvironment. Our analyses, including single-cell data (Supplemental Figure 14A) and flow cytometry (Supplemental Figure 14, B and C), revealed an increase in CD103^+^ immune cells. We also found that CD103 was primarily expressed on M1-like TAMs and T cells, suggesting that the E-cadherin–CD103 interaction may facilitate infiltration of these immune populations (Supplemental Figure 14D). However, scRNA-seq data revealed that only approximately 2% (927 out of total 45,627 cells) of tumor-infiltrating cells expressed CD103, and its expression level was generally low across all immune clusters (Supplemental Figure 14E). Given that macrophages comprise over 70% of immune cells in the melanoma TME, the CD103–E-cadherin interactions may play a minor role in ZDHHC13-mediated immune modulation in this context.

### SMPD2 is downstream of ZDHHC13 to induce M2-like TAM polarization.

SM synthesis involves converting ceramide (Cer) and PC into SM and diacylglycerol (DAG). An increase in SM synthesis could therefore result in reduced PC and LPC levels, aligning with our lipidomics findings (Figure 5G). Based on these observations, we hypothesize that ZDHHC13 may impact SM and LPC metabolism pathways to influence tumor immune responses.

**Figure 7:**
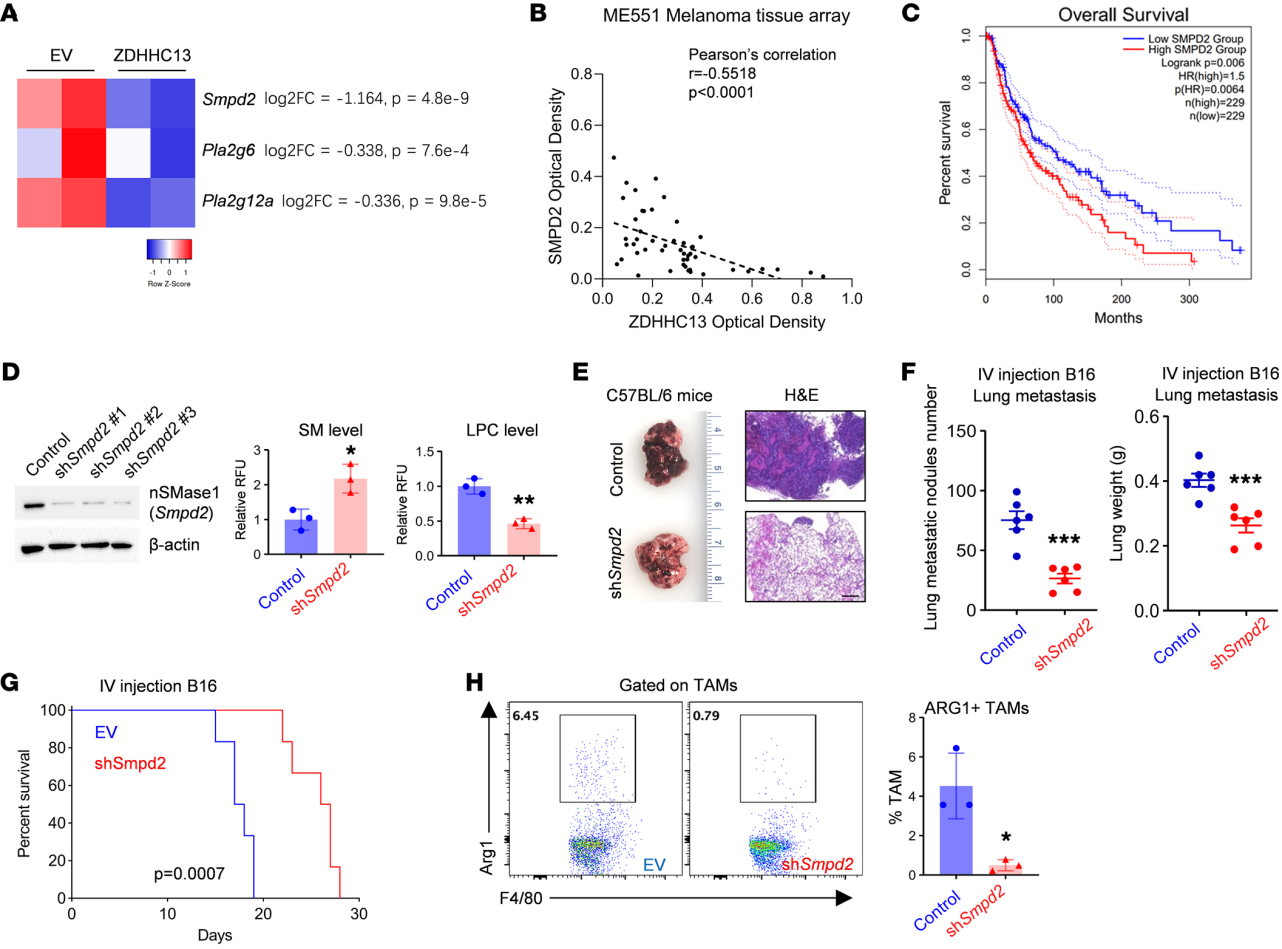
SMPD2 is a downstream of ZDHHC13 to induce M2-like TAM polarization. (**A**) RNA-seq analysis of B16 cells with or without ZDHHC13 expression showing genes involved in LPC and SM metabolism. (**B**) IHC of SMPD2 in melanoma tissue array; staining quantified in ImageJ and correlated by Pearson’s analysis. (**C**) Analysis of survival of patients with Melanoma based on SMPD2 expression (cutoff = 50%) calculated by GEPIA2. All patients in the TCGA melanoma study were divided according to the expression level of SMPD2 (higher or lower level than median expression value of all patients). (**D**) Validation of *Smpd2* knockdown in B16 cells, and quantification of SM and LPC levels by using Abcam SM and LPC assay kit (Abcam ab138877 and ab273332), respectively. (**E**–**G**) 2 × 10^5^ cells generated in **D** in 100 μl PBS were injected into C57BL/6 mice via the tail vein. Pulmonary metastases (**E** and **F**) and lung H&E staining (**E**) were assessed 14 days after tumor cell injection. Mouse survival was recorded (**G**) (*n* = 6). Scale bar: 185 μm. (**H**) Flow cytometry analysis of ARG1^+^ M2-like macrophages in lungs from mice in **E** (*n* = 3). All data in this Figure are mean ± SD. *P < 0.05, **P < 0.01, ***P < 0.001, unpaired Student’s *t* test.

We further investigated genes linked to LPC and SM metabolism in the melanoma RNA-seq analysis dataset (Figure 5A, Figure 7A, and Supplemental Figure 15A). Genes associated with phospholipase A2 (PLA2) (*Pla2g12a* and *Pla2g6*), the key enzyme for LPC synthesis, were downregulated, as were genes related to Sphingomyelin phosphodiesterase (SMPD) (*Smpd2*), responsible for SM breakdown. Consistent findings were observed in human datasets (Supplemental Figure 15, B–D), with *SMPD2* being the sole gene implicated in both LPC and SM metabolism across the mouse and human datasets. We conducted an additional analysis using a human melanoma tissue array (ME551) and observed a strong inverse correlation between ZDHHC13 and SMPD2 protein expression (Figure 7B). Moreover, elevated *SMPD2* expression was positively correlated with increased M2 macrophage infiltration (Supplemental Figure 15E), higher *MMP12* expression (Supplemental Figure 15F), and poorer survival outcomes in melanoma patients (Figure 7C), further validating and reinforcing the clinical relevance of our findings.

Notably, *Smpd2* knockdown in B16 cells significantly increased SM levels and decreased LPC in a cell culture experiment (Figure 7D). To investigate whether SMPD2 promotes melanoma development by inducing M2-like TAM polarization, we conducted experiments using murine models. Subcutaneous injection of B16 melanoma cells into immunodeficient mice showed no significant differences in tumor growth or survival between control and *Smpd2* knockdown groups (Supplemental Figure 16, A–C). Similarly, tail vein injections revealed no significant effects on melanoma lung colonization or mouse survival (Supplemental Figure 16, D–F). However, in immunocompetent C57BL/6J mice, *Smpd2* knockdown in B16 melanoma cells significantly delayed both melanoma growth (Supplemental Figure 16, G–J) and lung metastasis (Figure 7, E–G). Flow cytometry analysis confirmed a significant reduction in ARG1^+^ M2-like TAMs in the *Smpd2* knockdown melanoma metastases (Figure 7H). Additionally, the expression of M2 signature genes *Arg1*, *Vegfa*, *Spp1*, and *Mmp12* was significantly lower in these metastases (Supplemental Figure 16K). These findings underscore the critical role of SMPD2 in facilitating melanoma progression and immune evasion in mouse models.

To explore how SMPD2 is regulated by ZDHHC13, we performed IP followed by mass spectrometry to identify potential substrates of ZDHHC13 in melanoma cells. Our analysis did not reveal SMPD2 as a direct palmitoylation target of ZDHHC13 (Supplemental Figure 17A). However, we observed that many ZDHHC13-interacting proteins showed significant correlation with SMPD2 and PLA2G15 expression levels in human melanoma datasets (Supplemental Figure 17, B and C). This suggests that ZDHHC13 may indirectly regulate SMPD2 through modulation of multiple downstream effectors.

Among the candidate substrates identified, succinate dehydrogenase subunit A (SDHA) emerged as a particularly compelling hit. SDHA is a mitochondrial enzyme that plays a central role in the TCA cycle and the electron transport chain. Mitochondria are increasingly recognized not only as metabolic hubs but also as important regulators of lipid metabolism and immune responses. Notably, mitochondrial function has been linked to LPC (27–31), and by modulating lipid metabolism, mitochondria can influence signaling pathways under conditions of stress or immune activation. In addition, SDHA has been reported to regulate gene expression at the transcriptional level (32, 33), prompting us to hypothesize that its palmitoylation may impact SMPD2 expression and LPC synthesis.

To test this, we validated that ZDHHC13 overexpression enhances SDHA palmitoylation (Supplemental Figure 17D). Through site-directed mutagenesis, we identified Cys654 (C654) as the major palmitoylation site on SDHA (Supplemental Figure 17E). To assess the functional relevance of this modification, we performed *SDHA* knockdown experiments and observed a marked reduction in *SMPD2* expression. Reintroduction of WT SDHA restored *SMPD2* levels, whereas the palmitoylation-deficient mutant (C654S) caused an even greater increase in SMPD2 expression (Supplemental Figure 17, F and G). These findings suggest that palmitoylation of SDHA by ZDHHC13 negatively regulates SMPD2 expression, offering a potential mechanistic link between mitochondrial signaling, lipid metabolism, and immune modulation in melanoma.

Collectively, these findings suggest that palmitoylation of SDHA by ZDHHC13 suppresses SMPD2 expression. Importantly, we emphasize that SDHA is likely one of multiple ZDHHC13 substrates that contribute to the regulation of SMPD2 and LPC metabolism. Other ZDHHC13-interacting proteins identified in our screen may also play supportive or parallel roles in this regulatory network.

### LPC stimulates M2-like TAM signature gene expression.

**Figure 8:**
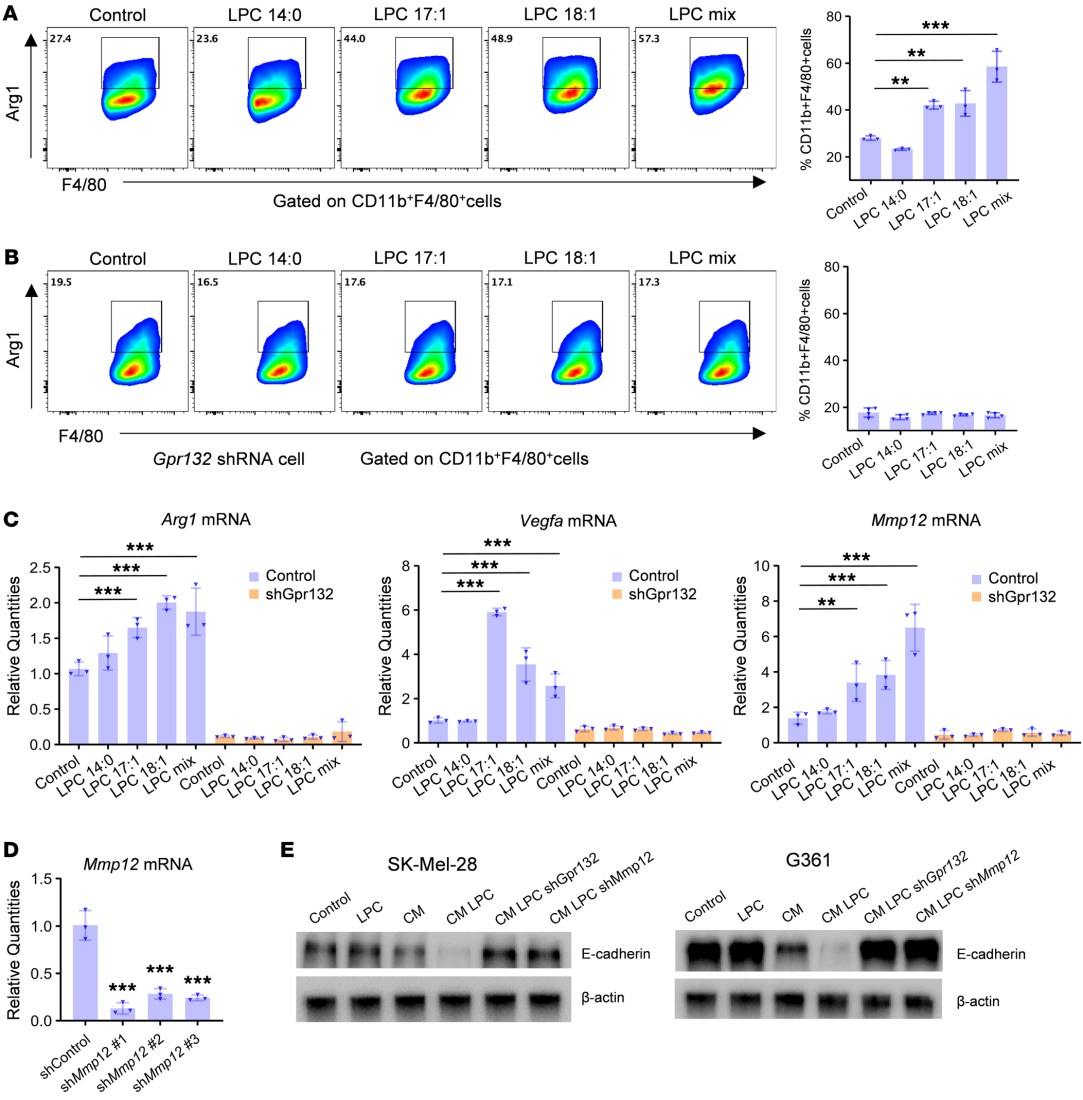
LPC stimulates M2-like TAM signature gene expression. (**A** and **B**) LPC treatment significantly increased M2-like TAM marker *Arg1* expression in raw 264.7 macrophages. Cells were pretreated with IL-4, TGF-β, and IL-10 mix (10 ng/ml) for 24 hours to induced M2-like polarization, then incubated with indicated LPC (1 μM) for 24 hours. Flow cytometry was used to detect ARG1^+^/F4/80^+^ M2 macrophages (*n* = 3). (**C**) Raw 264.7 macrophages were treated the same as in **A** and **B**, then total RNA samples were collected for qRT-PCR analysis. (**D**) Confirmation of *Mmp12* knockdown in raw 264.7 macrophages by using qRT-PCR (*n* = 3). (**E**) Melanoma cells were treated with LPC (1 μM) or conditional medium (CM) from LPC mix treated raw 264.7 macrophages as described in **A** and **B** for 24 hours, then protein samples were collected for Western blot analysis. All data in this Figure are mean ± SD. *P < 0.05, **P < 0.01, ***P < 0.001, multiple comparisons by Dunnett’s test.

LPC is known to influence cell functions through the G protein–coupled receptor G2A (*GPR132*) in immune cells (34). To investigate whether LPC directly impacts macrophage polarization, we assessed the effects of 3 LPC species significantly altered in our lipidomics study — 14:0, 17:1, and 18:1 — as well as a mixed LPC species on Raw 264.7 mouse macrophages (Figure 8A). All LPC species tested, except LPC 14:0, significantly increased M2 polarization, indicated by the upregulation of the M2 marker *Arg1*, though to varying extents. Moreover, *G2a* knockdown in mouse macrophages completely abolished the effects of LPC on M2 polarization (Figure 8B and Supplemental Figure 18, A and B), suggesting that LPC induces M2 polarization through G2A. Quantitative RT-PCR further confirmed that, apart from LPC 14:0, all tested LPC species markedly increased the expression of M2 signature genes such as *Arg1*, *Vegfa*, *Spp1*, and *Mmp12* in macrophages (Figure 8C and Supplemental Figure 18C). The abrogation of these effects following G2A knockdown corroborates the role of LPC in promoting melanoma immune evasion by activating M2-like TAMs.

Among the M2 signature genes identified in our single-cell analysis, MMP12 emerged as a marker for ARG1^+^ M2-like TAMs. As a secreted enzyme, MMP12 degrades ECM components and cell adhesion molecules, including E-cadherin, crucial for tissue remodeling (35–37). MMP12 expression is significantly higher in human metastatic melanoma samples (Supplemental Figure 18D). It is the most enriched gene in the ARG1^+^ M2-like TAM cluster (Supplemental Figure 11F) and was significantly reduced in TAMs following ZDHHC13 overexpression in melanomas (Supplemental Figure 11G, and Supplemental Figure 18E). While other MMP family proteins such as MMP13 and MMP14 were also identified, MMP13 was expressed at lower levels than MMP12, and MMP14 was not exclusive to the ARG1^+^ M2-like TAMs (Supplemental Figure 18F). Given MMP12’s role in degrading E-cadherin, we explored whether ZDHHC13 could upregulate E-cadherin indirectly by suppressing MMP12 in macrophages. Our experiments revealed that, while direct LPC treatment of melanoma cells did not alter E-cadherin levels, conditioned medium (CM) from macrophages and LPC-treated macrophages reduced E-cadherin protein levels on melanoma cells. Importantly, this effect was negated by *G2a* or *Mmp12* knockdown in macrophages (Figure 8, D and E). These findings suggest that, in addition to promoting CTNND1 palmitoylation in melanoma cells, ZDHHC13 may also stabilize E-cadherin in melanoma cells by reducing LPC levels in the TME and suppressing MMP12 expression in M2-like TAMs.

### Dual functions of ZDHHC13 in melanoma metastasis suppression.

To unify our mechanistic insights, we developed an integrated model that encompasses both the tumor-intrinsic and immune-regulatory functions of ZDHHC13. This model connects the cis-acting pathway — mediated by CTNND1 palmitoylation and subsequent stabilization of E-cadherin — with the trans-acting pathway, wherein LPC modulates macrophage behavior through the G2A receptor.

**Figure 9:**
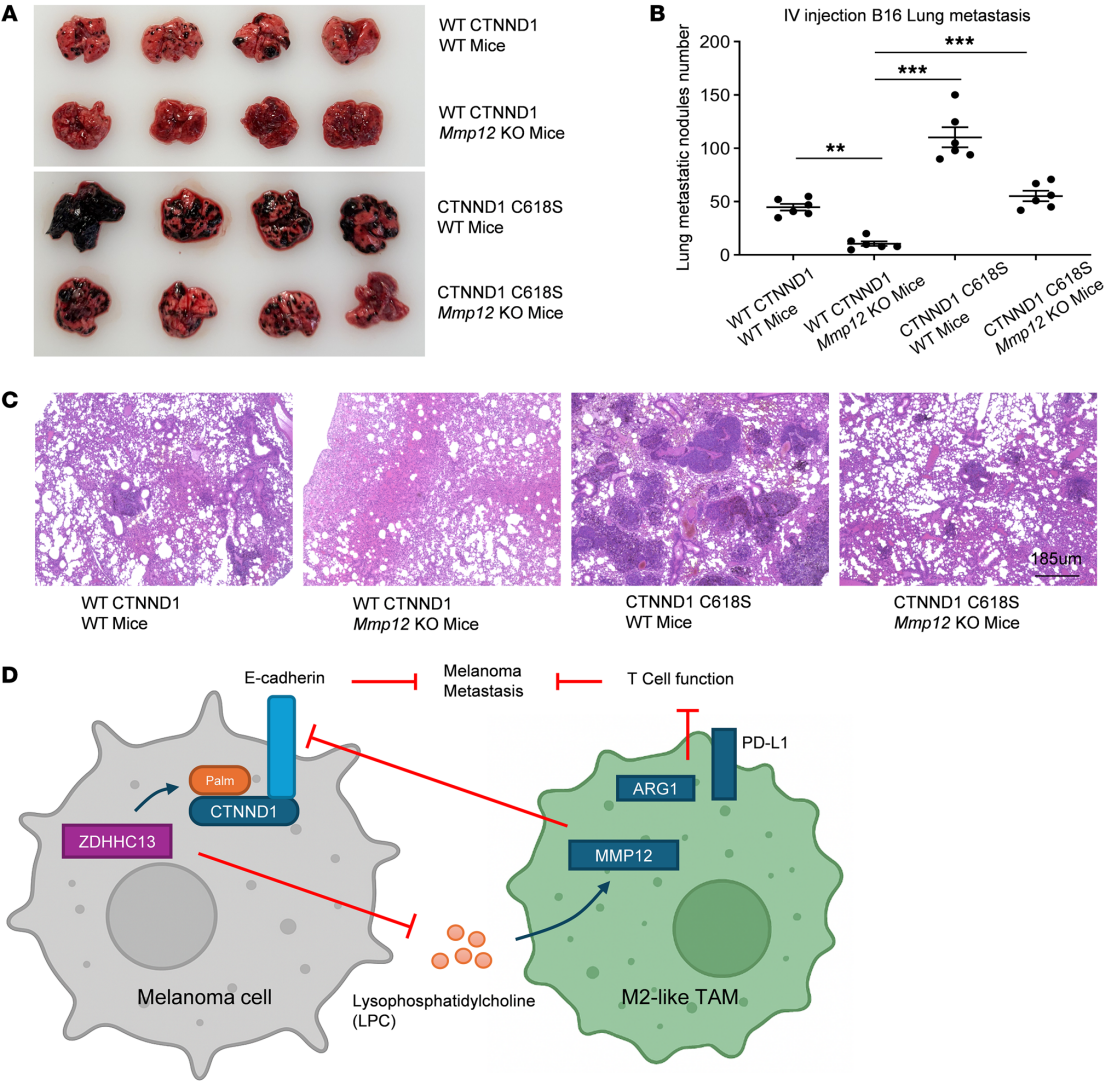
Dual mechanisms of ZDHHC13-mediated melanoma metastasis suppression. (**A**) 2 × 10^5^ B16 stably expressing WT or C618S CTNND1 in 100 μl PBS were injected into C57BL/6 mice or C57BL/6 Mmp12 knockout (KO) mice via the tail vein. Pulmonary metastases were assessed 14 days after tumor cell injection. Data are mean ±SD (*n* = 6). *P < 0.05, **P < 0.01, ***P < 0.001, group comparisons were performed using Dunnett’s test. (**B**) H&E staining of lungs displayed in **A**. Scale bars: 185 μm. (**C**) ZDHHC13 regulates melanoma metastasis via 2 parallel and partially overlapping pathways: (a) by enhancing CTNND1 palmitoylation and stabilizing E-cadherin (Figures 1–3), and (b) by modulating the immune microenvironment through macrophage-mediated mechanisms (Figures 5–7).

To specifically examine the immune component, we utilized *Mmp12* knockout mice. Because *Mmp12* is exclusively expressed in macrophages, mice homozygous for the targeted mutation are viable, fertile, and phenotypically normal, providing a clean system to study macrophage-specific effects. Importantly, our data show that concurrent activation of both downstream arms of ZDHHC13 signaling — enhanced CTNND1 palmitoylation and suppression of macrophage-derived MMP12 — produces the most robust inhibition of melanoma metastasis (Figure 9, A and B, and Supplemental Figure 19, A and B).

Collectively, these findings support a dual-pathway model in which ZDHHC13 suppresses metastasis through 2 parallel and partially overlapping mechanisms (Figure 9C): (a) a tumor-intrinsic pathway involving CTNND1 palmitoylation and E-cadherin stabilization (Figures 1–3), and (b) an immune-regulatory pathway involving modulation of the tumor microenvironment via macrophage polarization (Figures 5–8).

## Discussion

Dysregulated lipid metabolism contributes to tumor initiation and progression and has a profound impact on antitumor immunity. Metabolic reprogramming in cancer cells drives lipid accumulation in the TME, where fatty acids influence the function of infiltrating immune cells. Nonetheless, the impact of lipid metabolism on immune responses within the TME presents a paradox. On the one hand, elevated fatty acid levels in the TME have been linked to the enhancement of CD8^+^ T cell effector functions, as indicated by previous reports (38, 39). Conversely, contrasting studies have revealed that the accumulation of very long–chain fatty acids in the breast cancer TME can inhibit CD8^+^ T cell–mediated tumor suppression (40, 41). Moreover, lipids have been observed to bolster immune responses by enhancing Trm cells, NKT cells, and M1-like macrophages, while they have also been found to suppress tumor immunity by promoting the function of Treg cells, DCs, and M2-like macrophages (38, 42–48). Thus, lipids act as a double-edged sword, with effects that vary depending on tumor type and lipid composition, complicating efforts to target lipid metabolism therapeutically. Our findings identify LPC as a key regulator of immune responses in the TME, where it promotes an M2-like macrophage phenotype that supports tumor growth and immune suppression. ZDHHC13 reduces LPC levels, thereby reprogramming macrophages toward an M1-like state that enhances antitumor immunity. These results highlight the importance of considering specific lipid species, rather than global lipid metabolism, in shaping immune cell behavior. Broad targeting of lipid pathways may blunt protective responses or exacerbate protumor mechanisms, whereas focusing on lipid-specific interactions could inform more effective therapeutic strategies.

Palmitoylation, a lipidation process that regulates protein stability, localization, and function, also feeds back to control lipid metabolism. Our findings suggest a regulatory loop in which lipid-modified proteins influence metabolic pathways and cellular lipid pools. We recently showed that AMPK phosphorylates ZDHHC13 at S208, modulating its function (49). As ZDHHC13 regulates lipid metabolism through palmitoylation, AMPK may fine tune this process under metabolic stress to optimize lipid utilization. In addition, ZDHHC4 and ZDHHC5 stimulate fatty acid uptake by palmitoylating CD36 (50), ZDHHC7 palmitoylates GLUT4 to promote glucose uptake (51, 52), and palmitoylation of AKT at C344 is required for preadipocyte differentiation (53). Together, these examples underscore palmitoylation as a metabolic hub that integrates protein regulation with lipid metabolism, with broad implications for cancer and other metabolic diseases.

In addition to the upregulation of CTNND1 palmitoylation, our mass spectrometry analysis identified 3 downregulated proteins—EXOC5, NONO, and SPDL1. EXOC5, a component of the exocyst complex, is involved in vesicle trafficking and maintaining cell polarity (54). NONO is a nuclear RNA–binding protein implicated in transcriptional regulation and DNA repair, with emerging but context-dependent roles in cancer progression (55). SPDL1 (Spindly), known for its role in mitotic checkpoint control, may contribute to tumor evolution through effects on genomic stability (56). The functional relevance of these proteins in melanoma metastasis remains unclear and warrants further investigation.

TAMs, particularly those exhibiting an M2-like phenotype, are implicated in promoting tumor metastasis through a variety of molecular pathways (57, 58). Consistent with previous findings, we found key mediators regulated by M2-like TAMs, including VEGF, PD-L1, ARG1, and SPP1 (59). Each of these factors plays a distinct role in facilitating metastatic progression. In addition, we also identified a marker, MMP12, in ARG1^+^ TAMs. MMPs are a family of zinc-dependent endopeptidases that play a crucial role in the degradation of the extracellular matrix (35) and E-cadherin (36, 37). MMP12 is one of the MMPs predominantly expressed by macrophages (60, 61), and elevated MMP12 expression is found in various cancers (62-64).

E-cadherin is a cell adhesion molecule that maintains epithelial integrity, and its loss is a hallmark of epithelial-to-mesenchymal transition (EMT), which promotes invasion and metastasis (65). Paradoxically, some studies suggest that E-cadherin can promote metastasis in breast cancer models (15), underscoring context-dependent roles. In melanoma, early reports described moderate to strong staining (16, 17), but more consistent analyses show reduced E-cadherin expression in metastatic tumors compared with primary lesions (18, 19). In our in vivo model, E-cadherin overexpression suppressed lung metastasis, supporting its function as a melanoma metastasis suppressor. Other groups have similarly demonstrated its suppressive role across melanoma models (20–23).

Beyond E-cadherin, we observed enrichment of proteins that preferentially associate with nonpalmitoylated CTNND1, including filaggrin (FLG), plakophilin-1 (PKP1), and transglutaminase 1 (TGM1), all linked to poor clinical outcomes in metastatic melanoma (66). These interactions suggest that loss of CTNND1 palmitoylation may shifts its role from adhesion toward proinvasive signaling. Given that FLG, PKP1, and TGM1 are ECM related, their association with CTNND1 raises the possibility that altered adhesion dynamics converge with ECM remodeling to promote melanoma progression. This process may remain partially dependent on E-cadherin expression or localization, pointing to a complex interplay between adhesion molecules and cytoskeletal or ECM-regulatory pathways.

Our research highlights the role of ZDHHC13 in stabilizing E-cadherin to suppress melanoma metastasis through both melanoma-autonomous and nonautonomous mechanisms. Specifically, ZDHHC13 palmitoylates CTNND1, enhancing E-cadherin stability on the melanoma cell membrane. Furthermore, ZDHHC13 suppresses the production of LPC in the TME, which, in turn, limits the activation of M2-like TAMs. Reduced M2-like TAM activity leads to decreased secretion of MMP12 to prevent E-cadherin degradation. Our research identified a dual pathway by which ZDHHC13 stabilizes E-cadherin, thereby inhibiting melanoma metastasis. By targeting both intrinsic melanoma cell mechanisms and the broader tumor microenvironment, our findings provide a comprehensive framework for understanding ZDHHC13 signaling and its impact on melanoma metastatic progression.

## Methods

### Sex as a biological variable.

In our experiment, sex was considered as a biological variable by ensuring a sex-balanced design, with equal representation of male and female individuals. This balance was maintained throughout the recruitment process to ensure that both sexes were equally represented, minimizing any potential bias due to sex-based differences. Previous studies have demonstrated that B16 grow at similar rates in male and female C57BL/6J mice (67); both males and females (68, 69) were previously used for B16 allograft experiments.

### Statistics.

Statistical analyses were performed using GraphPad Prism. For comparisons between 2 groups, unpaired 2-sample *t* tests were applied. For comparisons of multiple treatment groups against a common control, Dunnett’s test was used to correct for multiple comparisons. *P* < 0.05 was considered statistically significant.

### Study approval.

All animal experiments were conducted in accordance with the Guide for the Care and Use of Laboratory Animals, 8th Edition (2011), published by the National Research Council of the National Academies. All protocols were approved by the Institutional Animal Care and Use Committee (IACUC) at Boston University School of Medicine and the Cleveland Clinic.

### Data availability.

Sequencing data have been deposited in the NCBI Gene Expression Omnibus (GEO) under accession number GSE302431 and GSE302433. Supporting data values are provided in the Supporting Data Values file, which includes multiple tabs. All other relevant data are available within the article and its Supplemental materials. Raw data are available from the corresponding author upon reasonable request.

## Author contributions

SC was responsible for conceptualization, funding acquisition, resource provision, and supervision of the study. Methodology was developed by HL, JL, YS, CY, and YL. Formal analysis was conducted by HL, JL, YS, CY, YL, and SC. Experimental investigations were carried out by HL, JL, YS, CY, and YL. WC, SSF, and XW provided materials support. All authors, including HL, JL, YS, XL, CY, YL, WC, SF, XW, CRG, and SC contributed to review and editing of the manuscript.

## Funding support

This work is the result of NIH funding, in whole or in part, and is subject to the NIH Public Access Policy. Through acceptance of this federal funding, the NIH has been given a right to make the work publicly available in PubMed Central.

- NIH grant K99/R00 CA234097 (SC).
- VeloSano Pilot Grants (SC).
- The Outrun the Sun Melanoma Research Scholar Program (SC).

## Supplementary Material

Supplemental data

Unedited blot and gel images

Supporting data values

## Version 1 10/02/2025

In-Press Preview

## Version 2 12/01/2025

Electronic publication
