# Supplementary material for: S-acyl transferase ZDHHC13 modulates tumor microenvironment interactions to suppress metastasis in melanoma models
Source: J Clin Invest. 2025 Sep 30;135(23):e188249. doi: 10.1172/JCI188249 (PMC12646662; doi:10.1172/JCI188249)
Supplement: Supplemental data [file jci-135-188249-s140.pdf]

**Supplementary Methods**

**Clinical sample analysis in public database**

Clinical and transcriptomic data were systematically interrogated using four online platforms—cBioPortal, GEPIA2, TNMplot, and TIMER II—to ensure robust and complementary insights. For survival analyses in cBioPortal, GEPIA2, and TIMER II, TCGA (Skin Cutaneous Melanoma) SKCM mRNA expression values were dichotomized at the cohort-specific median expression level: samples with expression values strictly above the median were classified as "high," and those at or below the median as "low," thereby yielding balanced group sizes and reducing outlier effects; overall survival differences between these two groups were then assessed by Kaplan–Meier estimation with log-rank testing. Immune cell infiltration was quantified in TIMER II via the xCell algorithm, and infiltration scores were correlated with gene expression levels using Spearman's rank correlation. Comparative expression analyses across normal tissue, primary tumor, and metastatic samples were performed in GEPIA2 (leveraging TCGA and GTEx data) and TNMplot— the latter aggregating 56,938 samples (15,648 normal, 40,442 tumors, and 848 metastasis) from GEO, GTEx, TCGA, and TARGET. In GEPIA2, two-tailed Wilcoxon rank-sum tests were employed for pairwise group contrasts; in TNMplot, a Kruskal–Wallis test first assessed overall differences among the three groups, followed by post-hoc Dunn's multiple-comparison tests (with adjusted p-values) to identify specific groupwise differences.

**Immunohistochemistry**

Immunohistochemistry for the human melanoma tissue array (ME551, TissueArray.Com LLC) comprising 27 primary melanoma and 22 metastatic samples was performed using the Discovery ULTRA automated stainer (Roche Diagnostics, Indianapolis, IN). Antigen retrieval was carried out using a tris/borate/EDTA buffer (Discovery CC1, pH 8.0–8.5; Cat# 06414575001, Roche) at 95°C for 32 minutes for all antibodies. Tissue sections were then incubated with primary antibodies under the following conditions: SMPD2 (Rabbit Polyclonal, Cat# HPA018125, Sigma-Aldrich) was incubated at room temperature for 32 minutes at a dilution of 1:200; ZDHHC13 (Rabbit Polyclonal, Cat# 24759-1-AP, Proteintech) for 1 hour at room temperature at 1:50 dilution; MMP12 (Rabbit Monoclonal, Clone SR03-23, Cat# MA5-32001, Invitrogen) for 36 minutes at 36°C at a 1:100 dilution; and E-Cadherin (Rabbit Monoclonal, Clone 24E10, Cat# 3195, Cell Signaling) for 1 hour at room temperature at a 1:150 dilution. Antibody binding was visualized using the OmniMap anti-Rabbit HRP detection system (Cat# 05269679001, Roche) and the Discovery Red detection kit (Cat# 09576819001, Roche). Finally, the slides were counterstained with hematoxylin and bluing. Captured digital bright-field images were imported into ImageJ for signal quatification. Chromogenic signal was isolated with the Color Deconvolution plugin using the "FastRed FastBlue" preset; the resulting single-channel image was thresholded automatically and visually inspected to remove artefacts. Regions of interest (ROIs) encompassing the stained areas were delineated, and the mean gray value (I) for each ROI was recorded. Optical density (OD) was calculated as $OD = \log_{10}(255/I)$. Statistical analysis was performed in GraphPad Prism: differences between two groups were assessed with an unpaired, two-tailed Student's t-test, and associations between variables were examined with Pearson's correlation; $p<0.05$ was considered significant.

**Immunoblots and immunoassays**

Western blotting, immunoprecipitation (IP), and acyl-biotin exchange (ABE) assays for palmitoylation were carried out following previously published protocols (1-3). Cells were lysed in ice-cold lysis buffer composed of 50 mM Tris-HCl (pH 7.4), 1% Triton X-100, 0.5 mM EDTA, 0.5 mM EGTA, 150 mM NaCl, and 10% glycerol, supplemented with protease and phosphatase inhibitor cocktail (Pierce #78446) to prevent protein degradation and dephosphorylation. Cells were incubated in lysis buffer for 30 minutes on ice with periodic vortexing to facilitate membrane disruption and protein solubilization. Lysates were clarified by centrifugation at 15,000xg for 15 minutes at 4°C, and the supernatants were collected and quantified using a BCA Protein Assay Kit (Thermo Scientific) to ensure equal protein input. For immunoprecipitation, equal amounts of total protein (typically 500-1000 µg per sample) were transferred to fresh tubes and precleared by incubation with 20 µL of Protein G agarose beads (Thermo Scientific #20397) for 1 hour at 4°C with gentle rotation to reduce non-specific binding. After preclearing, the supernatants were incubated overnight at 4°C with either primary antibodies against the target protein or with anti-Flag or anti-HA agarose-conjugated beads (Sigma #A2220 and #A2095, respectively). For non-conjugated antibodies, 20 µL of fresh Protein G agarose beads were added after the overnight antibody incubation and rotated for an additional 2 hours at 4°C to capture immune complexes. Following incubation, beads were washed four times with ice-cold lysis buffer to remove non-specifically bound proteins. The eluted proteins were separated by SDS-PAGE and transferred onto PVDF membranes. Membranes were blocked in 5% non-fat dry milk or 5% BSA in TBS-T (Tris-buffered saline with 0.1% Tween-20) for 1 hour at room temperature and probed with primary antibodies overnight at 4°C. After washing, membranes were incubated with appropriate HRP-conjugated secondary antibodies for 1 hour at room temperature. Chemiluminescent signals were developed using ECL substrate (Thermo Scientific). The

antibodies used included Streptavidin-HRP (1:2000) (Thermo Scientific #21130), ZDHHC13 (1:1000) (Abcam #ab28759, RRID:AB_1603048), β-Actin-HRP (1:20000) (Sigma #A5441), Flag-HRP (1:2000) (Sigma #A8592), HA-HRP (1:1000) (Sigma #H6533), anti-mouse secondary antibody (1:2000) (Sigma #A4416), anti-rabbit secondary antibody (1:2000) (Sigma #A-4914), E-cadherin (1:1000) (Cell Signaling #3195, #14472), CTNND1 (1:1000) (Origene OTI2E8, CAT#: CF800807), $Na^+/K^+$-ATPase (1:1000) (Cell Signaling #3010), GAPDH (1:5000) (Cell Signaling #5174, RRID:AB_10622025), SDHA (1:1000) (Abcam #ab14715), ZDHHC17 (1:500) (Thermo Scientific PA5-18336), ZDHHC21 (1:500) (Thermo Scientific PA5-25096). All immunoblots shown represent results from three independent experiments. Additional reagents included Flag agarose beads (Sigma #A2220) and HA agarose beads (Sigma #A2095).

### Acyl-biotin exchange (ABE) assays

Protein palmitoylation was assessed using the ABE assay, a sensitive and non-radioactive method to selectively detect thioester-linked S-palmitoylation on cysteine residues. Cells were first lysed on ice in a buffer containing 1% IGEPAL CA-630, 50 mM Tris-HCl (pH 7.5), 150 mM NaCl, 10% glycerol, a protease inhibitor cocktail (Pierce #78446), and 50 mM N-ethylmaleimide (NEM; Sigma-Aldrich), which irreversibly blocks all free cysteine thiols. Lysates were incubated for 2 hours at 4°C with gentle rotation to ensure complete alkylation of free thiols, thereby preventing nonspecific biotin labeling during later steps. After clarification by centrifugation at 15,000xg for 15 minutes at 4°C, target proteins were immunoprecipitated using either protein-specific antibodies bound to Protein G agarose beads or directly with affinity-conjugated anti-Flag or anti-HA agarose beads. Immunoprecipitates were washed extensively in lysis buffer to remove unbound material, and the beads were divided into two equal fractions for parallel processing. One fraction was treated with freshly prepared 1 M hydroxylamine (HAM) in lysis buffer (adjusted to pH 7.4) for 1 hour at room temperature to selectively cleave the thioester bond between palmitate and the cysteine thiol, thus revealing newly exposed thiol groups. The control fraction was incubated under identical conditions with Tris buffer lacking HAM to account for non-thioester-based background signals. Following HAM or mock treatment, both fractions were washed three times with lysis buffer adjusted to pH 6.2 to stabilize thiol groups and prevent reoxidation. Samples were then incubated in the same buffer containing 2 µM biotin-BMCC (Thermo Fisher Scientific) for 1 hour at 4°C to covalently label the newly exposed thiols with biotin. After labeling, beads were washed thoroughly to remove excess biotin reagent and proteins were processed for Western blotting analysis.

### Cell culture

B16-F10 (CRL-6475), SK-Mel-28 (HTB-72), G361 (CRL-1424), and HEK293T (CRL-3216) cell lines were obtained from the American Type Culture Collection (ATCC) and maintained according to ATCC recommendations. All cell lines were authenticated by short tandem repeat (STR) profiling and confirmed to be free of mycoplasma contamination using the LookOut Mycoplasma Detection Kit (Sigma-Aldrich #MP0035), with routine testing performed every 4–6 weeks. B16-F10 and HEK293T cells were cultured in Dulbecco's Modified Eagle Medium (DMEM; Gibco #11965092) supplemented with 10% fetal bovine serum (FBS; Gibco #26140079), 1% penicillin-streptomycin (Gibco #15140122), and 2 mM L-glutamine (Gibco #25030081). SK-Mel-28 and G361 cells were cultured in RPMI 1640 medium (Gibco #11875119) with the same supplements. Cells were maintained in humidified incubators at 37°C with 5% $CO_2$ and passaged when reaching approximately 80–90% confluence. After revival from cryopreservation, cells were cultured for five to six passages before being used in experiments. All experimental procedures were performed with early-passage cells (passage 5-20) to minimize phenotypic drift. Cell morphology was monitored regularly by phase-contrast microscopy, and any deviation from expected growth patterns prompted reauthentication or disposal of the cell line.

### Transwell Migration and Invasion Assays

Cell migration and invasion assays were performed using 24-well transwell inserts with 8 µm pore size polycarbonate membranes (Corning #3422). For the migration assay, $4x10^3$ cells were suspended in 200 µL of serum-free medium and seeded into the upper chamber of each insert. The lower chamber was filled with 600 µL of complete growth medium containing 10% fetal bovine serum (FBS) as a chemoattractant. Cells were incubated for 6 hours at 37°C in a humidified incubator with 5% $CO_2$ to allow migration. For the invasion assay, transwell inserts were pre-coated with 50 µL of Matrigel (Corning #354234) and incubated at 37°C for 1 hour to allow matrix solidification. The same number of cells ($4x10^3$) were seeded in the upper chamber in serum-free medium, with 10% FBS medium in the lower chamber as the chemoattractant. Invasion was allowed to proceed for 16 hours under identical incubation conditions. After incubation, non-migrated or non-invaded cells on the upper membrane surface were removed with a cotton swab. Cells that had migrated or invaded to the underside were fixed with 4% paraformaldehyde for 15 minutes, then stained with 0.1% crystal

violet for 20 minutes at room temperature. Membranes were washed, air-dried, and imaged under a bright-field microscope.

### Immunofluorescence

Immunofluorescence staining was performed on cultured cells seeded in chamber slides. Cells were rinsed briefly with PBS and fixed with freshly prepared 4% formaldehyde diluted in 1xPBS for 15 minutes at room temperature. Following fixation, specimens were incubated for 1 hour at room temperature in blocking buffer consisting of 1xPBS, 5% normal goat serum, and 0.3% Triton X-100 to permeabilize membranes and block non-specific binding. After blocking, the blocking solution was removed, and cells were incubated overnight at 4°C with the diluted primary antibody in a humidified, light-protected chamber. The following day, cells were rinsed three times with 1x PBS for 5 minutes each, then incubated for 2 hours at room temperature in the dark with fluorophore-conjugated secondary antibodies diluted in antibody dilution buffer. After secondary incubation, cells were washed three times in PBS, and coverslips were mounted using Prolong Gold Antifade Reagent with DAPI to preserve fluorescence and counterstain nuclei. Mounted slides were allowed to cure overnight at room temperature in the dark before imaging. Images were acquired using a confocal microscope under identical exposure settings for experimental comparisons. For quantitative analysis, the Coloc 2 plugin in ImageJ (Fiji) was used. Regions of interest (ROIs) were selected to exclude background and non-cellular regions. Coloc 2 analysis was performed using default parameters, and Manders' M1 coefficient was used to quantify colocalization. The M1 value represents the proportion of signal in one channel (e.g., CTNND1) that overlaps with the signal in a second channel (e.g., E-cadherin or $Na^+/K^+$ ATPase), providing a robust and compartment-specific measurement of colocalization. The following primary antibodies were used for immunofluorescence staining: E-cadherin (Cell Signaling, #3195, 1:200), CTNND1 (Origene, #CF800807, 1:200), and Na/K ATPase α1 (Cell Signaling, #23565, 1:200). For fluorescence detection, Alexa Fluor–conjugated secondary antibodies (Cell Signaling #4408, #8890, #4412, #8889) were used at a 1:500 dilution.

### Recombinant DNA Constructs

Plasmids encoding 24 Flag-tagged mouse Zdhhc family proteins, as well as Myc-Flag-tagged human CTNND1 (RC222771) and ZDHHC13 (RC208925), were purchased from Origene. These ORFs were subcloned into pLenti-Myc-Flag (PS100069) and pLenti-HA (PS100104) vectors using the Origene Precision Shuttling system. Mutations were introduced using the QuickChange II Site-Directed Mutagenesis kit (Agilent). Human and mouse shRNAs targeting CTNND1 (TRCN0000122984, TRCN0000122988, TRCN0000344830 for human; TRCN0000311512, TRCN0000109410, TRCN0000109412 for mouse), Smpd2 (TRCN0000105995, TRCN0000105996, TRCN0000105997), Gpr132 (TRCN0000027432, TRCN0000027454, TRCN0000027458), and Mmp12 (TRCN0000031254, TRCN0000031255, TRCN0000031256) were obtained from Sigma. Lentiviral expression plasmids were co-transfected with the packaging plasmid psPAX2 (RRID: Addgene_12260) and the envelope plasmid pMD2.G (RRID: Addgene_12259) at a 4:3:1 molar ratio using Lipofectamine 3000 (Thermo Scientific #L3000008), according to the manufacturer's protocol. Lentiviral supernatants were collected at 48- and 72-hours post-transfection, filtered through a 0.45 µm syringe filter (Millipore), and either used immediately or stored at -80°C. For transduction, target cells were seeded at ~40-50% confluence and exposed to viral supernatant supplemented with 8 µg/mL polybrene (Sigma-Aldrich #H9268) to enhance infection efficiency. Stably transduced cells were selected using 2 µg/mL puromycin for 3-5 days until all control (non-infected) cells were eliminated. Puromycin-resistant populations were either expanded for immediate use or cryopreserved.

### Mouse experiment

The ZDHHC13 transgenic mice used in these experiments were generated previously (1) by the Mouse ES Cell & Transgenic Facility at the Koch Institute for Integrative Cancer Research at MIT. The transgene was inserted downstream of the murine tyr promoter/enhancer region. Genotyping was performed by PCR on tail or ear DNA using primers that target a region spanning the tyr promoter/enhancer and ZDHHC13 (GGGCTATGTACAAACTCCAAGA, CAGCTTCCAAAAGCTTATCAACT). Tyr-Cre-$BRAF^{CA}$ mice (Stock No: 017837), Tyr-Cre-$BRAF^{CA}$-$Pten^{loxp}$ (Stock No: #013590), B6.129X-$Mmp12^{tm1Sds}$/J (Stock No: 004855), C57BL/6J (Stock No: #000664), NOD scid (Strain #:001303) mice were purchased from The Jackson Laboratory. All mice were housed in pathogen-free facilities at the Boston University Medical Campus and Cleveland Clinic, under a 12-hour light/dark cycle with free access to sterile food and water. For subcutaneous tumor growth experiments, B16 cells in 100 µL of sterile phosphate-buffered saline (PBS) were injected into the shaved flank of mice. Tumor dimensions were measured using digital calipers, and tumor volume was calculated using the formula: volume = (length x $width^2$)/2. Tumor weights were recorded at the endpoint. For experimental lung metastasis assays, $2x10^5$ B16 cells in 100 µL PBS were injected intravenously via the tail vein. Mice were monitored for 14 days, after which lungs were excised and analyzed

for metastatic burden. For melanoma-free survival experiments, Tamoxifen (1 mg per injection) was prepared by dissolving in corn oil, and a volume of 50 µL was injected intradermally into the shaved flank using a 30-gauge insulin syringe. Injections were repeated once daily for five consecutive days to ensure sufficient recombination and activation of $BRAF^{V600E}$ in melanocytes. Mice were randomly assigned to experimental groups. Humane endpoints included tumor volume larger than $2cm^3$, significant weight loss (>20%), inability to ambulate, eat, or drink, severe respiratory distress, or other signs of distress identified in consultation with a veterinarian. Sample size was determined based on prior experience with similar models, and investigators were blinded to group allocation.

## Flow cytometry

Single-cell suspensions were generated from mouse lungs and tumors following a previously established method (4). In brief, lungs and tumors were harvested from C57/BL6 mice implanted with B16F10. The tissues were finely minced and incubated with Collagenase type IV (Worthington) and DNase I (Roche) in RPMI medium for 45 minutes at 37°C. Following red blood cell lysis, live and dead cells were distinguished using the Live/Dead Fixable Aqua Dead Cell Stain Kit (Life Technologies). To prevent non-specific binding, Fc receptors were blocked by staining with anti-CD16/32. Surface staining was performed for 30 minutes at 4°C in FACS staining buffer (1x PBS, 5% FBS, 0.5% sodium azide) containing the designated antibody cocktails. For intracellular staining, cells were fixed and permeabilized using the BD Cytofix/Cytoperm kit, according to the manufacturer's protocol. Cells were stained with antibodies against MHC Class II (I-A/I-E), CD103 (2E7), NK-1.1 (PK136), Ly-6C (HK1.4), Ly-6G (Gr-1) (RB6-8C5), CD11c (N418), F4/80 (BM8), CD11b (M1/70), CD45 (30-F11), TCR beta (H57-597), CD4 (RM4-5), CD3e (17A2), Arginase 1 (A1exF5), CD8a (53-6.7), CD49b (DX5), and Siglec-F (S17007L). Flow cytometry data were acquired using a BD Fortessa cytometer (BD Biosciences) and analyzed with FlowJo software V10 (BD).

## Lipidomics

Lipidomics analyses were conducted by Creative Proteomics. The samples were thawed on ice and processed by adding 1.5 mL chloroform: methanol (2:1, v/v) and 0.5 mL ultrapure water. The mixture was ground for 180 seconds at 65 Hz, vortexed for 1 minute, and then sonicated for 30 minutes at 4°C. After sonication, the samples were centrifuged at 3,000 rpm for 10 minutes at 4°C, and the lower phase was transferred to a new tube and dried under nitrogen. The dried extract was resuspended in 200 µL of isopropyl alcohol: methanol (1:1, v/v), with 5 µL LPC (12:0) added as an internal standard. The mixture was then centrifuged again at 12,000 rpm for 10 minutes at 4°C, and the supernatant was collected for LC-MS analysis. The separation was conducted using UPLC coupled with Q Exactive MS (Thermo), utilizing an ACQUITY UPLC BEH $C_{18}$ column (100 × 2.1 mm × 1.7 µm). The mobile phase consisted of solvent A (60% acetonitrile + 40% water + 10 mM ammonium formate) and solvent B (10% acetonitrile + 90% isopropyl alcohol + 10 mM ammonium formate), with a gradient elution of 30% B at 0-1.0 minutes, 30%-100% B at 1.0-10.5 minutes, 100% B at 10.5-12.5 minutes, and a re-equilibration of 100%-30% B from 12.5-12.51 minutes, held at 30% B until 16 minutes. The flow rate was set at 0.3 mL/min, the column temperature at 40°C, and the sample manager temperature at 4°C. Mass spectrometry was conducted in both ESI+ and ESI- modes, with the following parameters for ESI+: heater temperature at 300°C, sheath gas flow at 45 arb, auxiliary gas flow at 15 arb, sweep gas flow at 1 arb, spray voltage at 3.0 kV, capillary temperature at 350°C, and S-Lens RF level at 30%. For ESI-, the heater temperature was 300°C, sheath gas flow was 45 arb, auxiliary gas flow was 15 arb, sweep gas flow was 1 arb, spray voltage was 3.2 kV, capillary temperature was 350°C, and S-Lens RF level was 60%. The raw data were aligned using Lipid Search software (Thermo) based on m/z values and retention times of the ion signals. Ions from both ESI+ and ESI- were merged and imported into the SIMCA-P program (version 14.1) for multivariate analysis. Principal component analysis (PCA) was initially employed for data visualization and outlier identification, followed by Partial Least Squares Discriminant Analysis (PLS-DA) or Orthogonal Partial Least Squares Discriminant Analysis (OPLS-DA) for regression modeling to identify potential biomarkers. Biomarkers were filtered based on variable importance in projection (VIP) values (VIP > 1.0), t-test ($p < 0.05$), and fold change (FC > 2).

## Mass Spectrometry

Protein identification by mass spectrometry was conducted at the Proteomics & Metabolomics Core of the Cleveland Clinic Lerner Research Institute. Protein samples were mixed with sample buffer and heated at 100°C for 5 minutes before being loaded onto an SDS-PAGE gel for separation. Following electrophoresis, the gel was immersed in a staining solution containing 0.3% Coomassie Brilliant Blue, 45% methanol, 10% glacial acetic acid, and 45% $dH_2O$, and agitated on a shaker for 30 minutes. The gel was then destained overnight using a solution of 20% methanol, 10% glacial acetic acid, and 70% $dH_2O$. After destaining, the protein bands were excised from the gel and sent to the Proteomics & Metabolomics Core for mass spectrometry analysis.

**Single-Cell RNA sequencing**

Single-cell transcriptome profiling of tumor-infiltrating CD45+ cells was carried out using the 10x Genomics Chromium platform (V1 chemistry) by Genomics Core of the Cleveland Clinic Lerner Research Institute. CD45+ cells were freshly isolated from B16F10 tumors or B16F10 tumors stably expressing ZDHHC13, implanted in C57/BL6 mice, 12 days post-inoculation. The sorted cells were re-suspended in 1x PBS with 0.04% BSA. Single-cell RNA sequencing libraries were prepared following the Chromium Single Cell 3' protocol (10X Genomics, document #CG000204), with a target of 10,000 single cells per sample using the 10x Genomics Chromium Controller for cDNA synthesis and cell barcoding. The cDNA quality and quantity were assessed using a Bioanalyzer High Sensitivity DNA assay, and this cDNA was used for subsequent steps, including fragmentation, end-repair, adapter ligation, and sample indexing. Library construction was confirmed using the same Bioanalyzer assay, and the resulting libraries were pooled and quantified using a Quantabio Q cycler. Sequencing was performed on an Illumina Novaseq 6000 high-throughput platform, using 28 cycles for the forward read and 91 cycles for the reverse read.

**References:**


1. Chen S, Han C, Miao X, Li X, Yin C, Zou J, et al. Targeting MC1R depalmitoylation to prevent melanomagenesis in redheads. *Nat Commun.* 2019;10(1):877.
2. Chen S, Zhu B, Yin C, Liu W, Han C, Chen B, et al. Palmitoylation-dependent activation of MC1R prevents melanomagenesis. *Nature.* 2017;549(7672):399-403.
3. Sun Y, Li X, Yin C, Zhang J, Liang E, Wu X, et al. AMPK phosphorylates ZDHHC13 to increase MC1R activity and suppress melanomagenesis. *Cancer Res.* 2023.
4. Turnis ME, Sawant DV, Szymczak-Workman AL, Andrews LP, Delgoffe GM, Yano H, et al. Interleukin-35 Limits Anti-Tumor Immunity. *Immunity.* 2016;44(2):316-29.

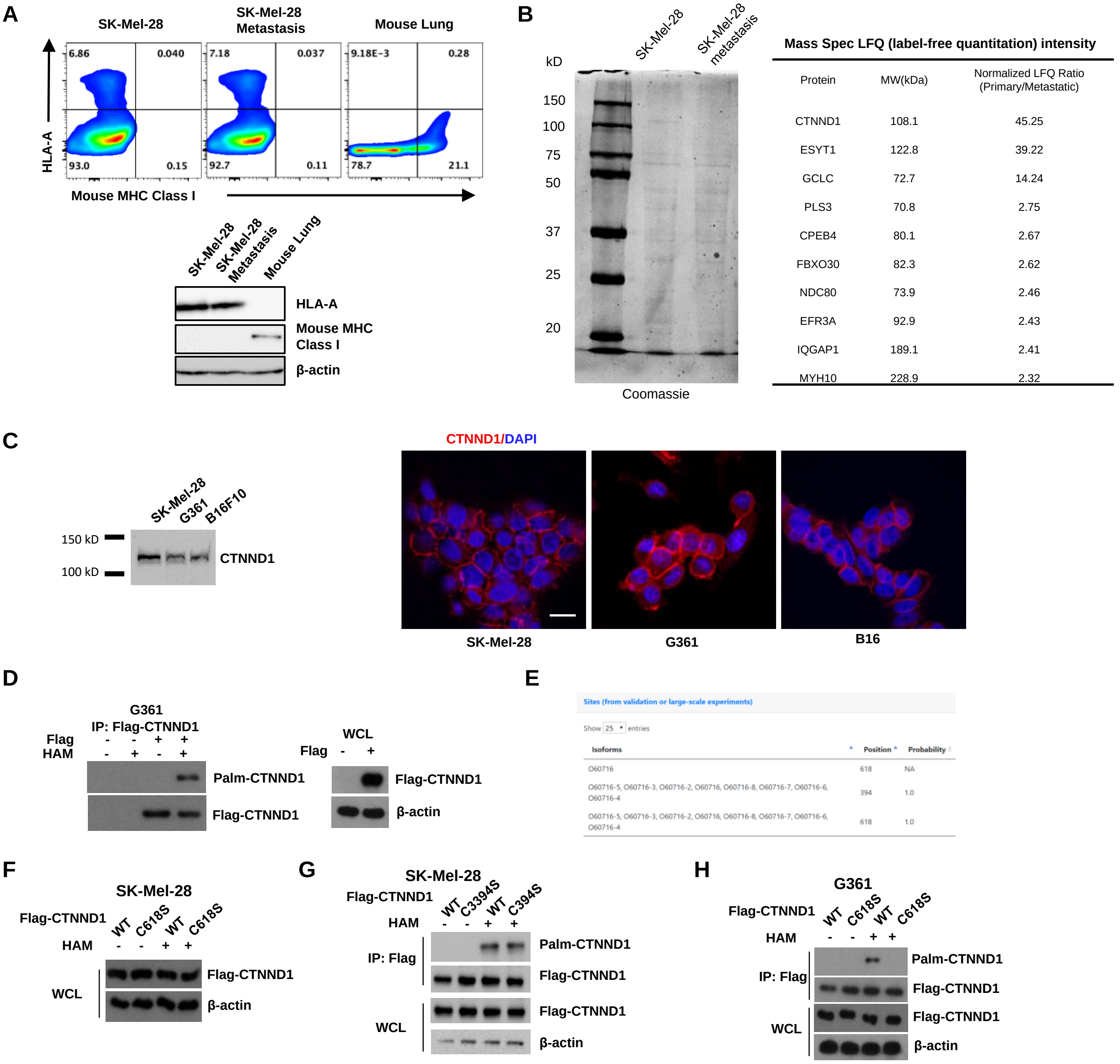


## Figure S1

**(A)** Flow cytometry and Western blot analyses were performed to assess expression of human HLA-A and mouse MHC class I molecules in tumor cells, mouse lung tissue extract was used as a mouse cell control. **(B)** SDS-PAGE analysis of palmitoylation enriched proteins in SK-Mel-28 cells and metastasized SK-Mel-28 cells, and Mass Spec LFQ (label-free quantitation) intensity result showed that CTNND1 is the most enriched palmitoylated protein in SK-Mel-28 compared to lung metastasis. **(C)** Expression and subcellular localization of CTNND1 in melanoma cell lines. **(D)** Exogenous expressed Flag-CTNND1 is palmitoylated in G361 cells, detected by ABE method. **(E)** Prediction of CTNND1 palmitoylation sites by SwissPalm. **(F)** The IB analysis of Flag-CTNND1 and β-actin in the whole cell lysate of experiment described in Figure 1G. **(G)** CTNND1 is not palmitoylated at C394 in SK-Mel-28 cells, detected by ABE method. **(H)** CTNND1 is palmitoylated at C618 in G361 cells, detected by ABE method.

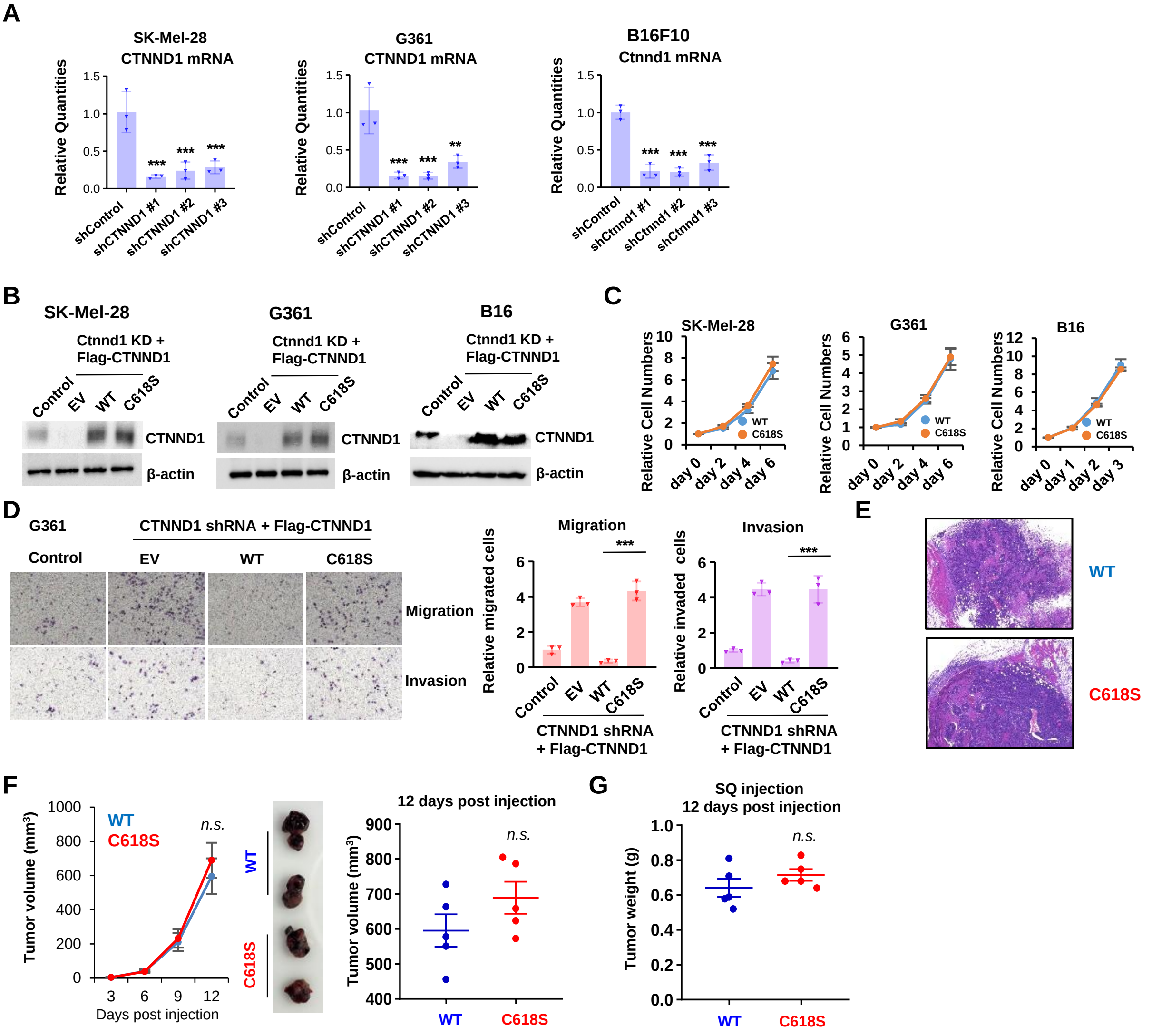


**Figure S2**

**(A)** Confirmation of CTNND1 knockdown in melanoma cell lines by qRT-PCR. Human shCTNND1#1 and mouse shCtnnd1#2 were selected for future experiments. **(B)** Deletion of endogenous expressed CTNND1 and reintroduction of WT or C618S CTNND1 in melanoma cell lines. **(C)** The relative cell growth of cell lines described in Figure S1J, counted by cell numbers. **(D)** $4x10^3$ G361 cells stably expressing WT or C618S CTNND1 with FBS-free medium were plated in the upper chamber of transwell filters (8 μm pore size) for 6 h (migration assay), or Matrigel coated transwells for 16 h (invasion assay). Three independent experiments were measured and calculated as mean ± SD, n=3. **(E)** Representative H&E staining of tumor collected in experiment performed in Fig. 1J. **(F-G)** $5X10^5$ B16 in 100ul PBS were subcutaneously injected into the flank of NOD scid mice. Tumor growth **(F)** and weight **(G)** were measured. Error bars represent ±SD (n=5). *p<0.05, **p<0.01, ***p<0.001, group comparisons in **(A)** and **(D)** were performed using Dunnett's test, others were tested by unpaired student's t-test.

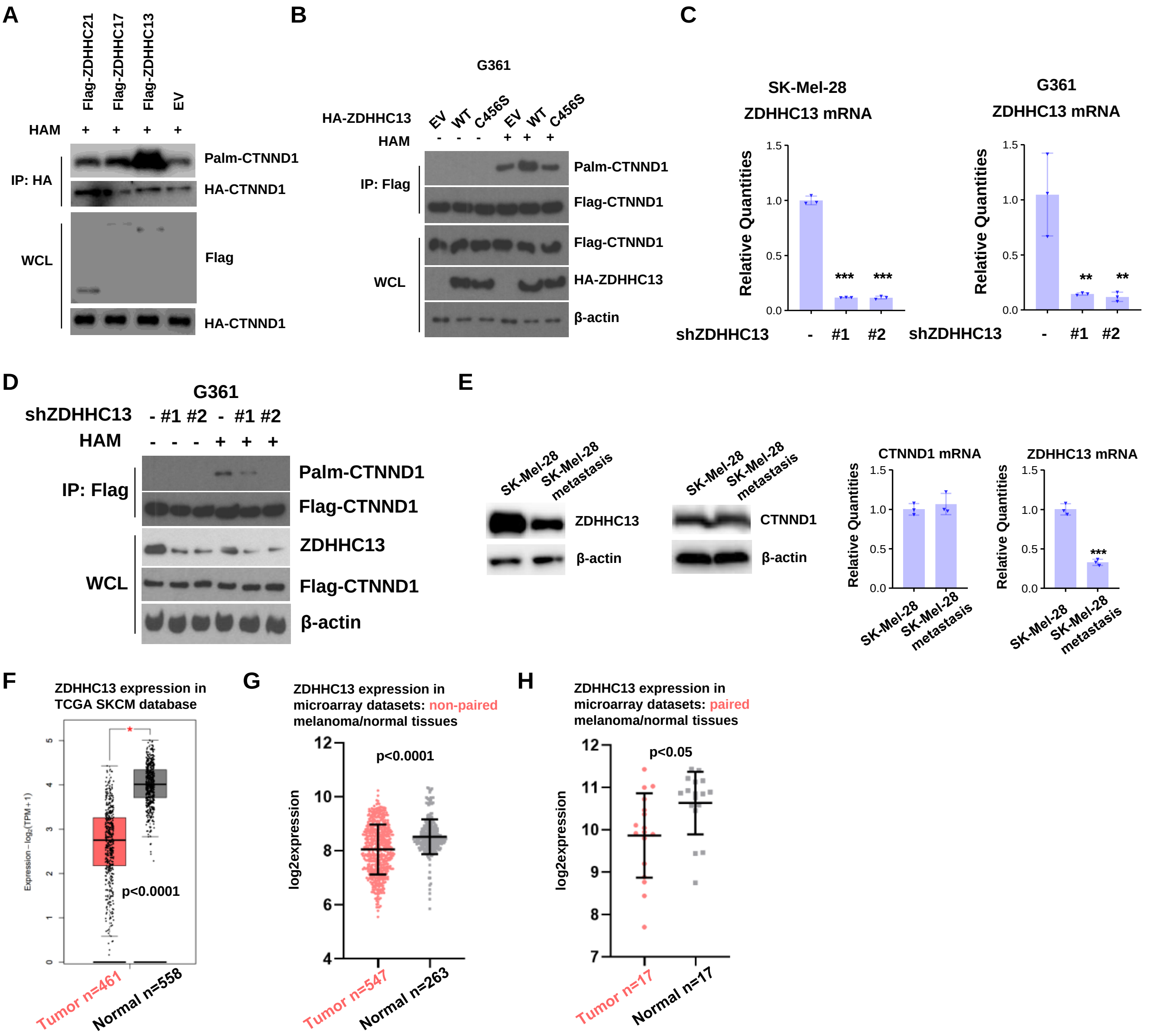


## Figure S3

**(A)** SK-Mel-28 cells were co-infected with constructs encoding HA-CTNND1 and Flag-ZDHHC. Cells lysates were harvested for IP by anti-HA antibody, and then for ABE and Western blot analysis. Western Blot for total CTNND1 and Flag-ZDHHC proteins was shown. **(B)** G361 cells were infected with the indicated Flag-CTNND1-encoding and HA-ZDHHC13 constructs, then the cells were subjected for IP, ABE and IB analysis. **(C)** Confirmation of ZDHHC13 knockdown in melanoma cell lines by qRT-PCR. **(D)** G361 cells were infected with the Flag-CTNND1 vectors or shZDHHC13 constructs, then the cells were subjected for IP, ABE and IB analysis. **(E)** Western blot analysis of ZDHHC13 and CTNND1 protein levels in SK-Mel-28 parental and metastatic cells. β-actin was used as a loading control. Quantitative RT-PCR analysis of ZDHHC13 and CTNND1 mRNA levels in SK-Mel-28 parental and metastatic cells. Data are representative of 3 independent experiments. Values are mean ± SD. **(F)** The mRNA expression of ZDHHC13 in tumor (TCGA SKCM) and non-tumor tissues (match TCGA normal and GTEx data). Data were calculated by GEPIA2 (Gene Expression Profiling Interactive Analysis). **(G)** ZDHHC13 expression comparison in melanoma and normal tissues in GENT2 database (GPL570 platform (HG-U133_Plus_2, including 23 microarray datasets GSE4587, GSE4845, etc.). **(H)** ZDHHC13 expression comparison in melanoma and paired adjacent normal tissues in TNMplot database. *$p<0.05$, **$p<0.01$, ***$p<0.001$, group comparisons in **(C)** was performed using Dunnett's test, others were tested by unpaired student's t-test.

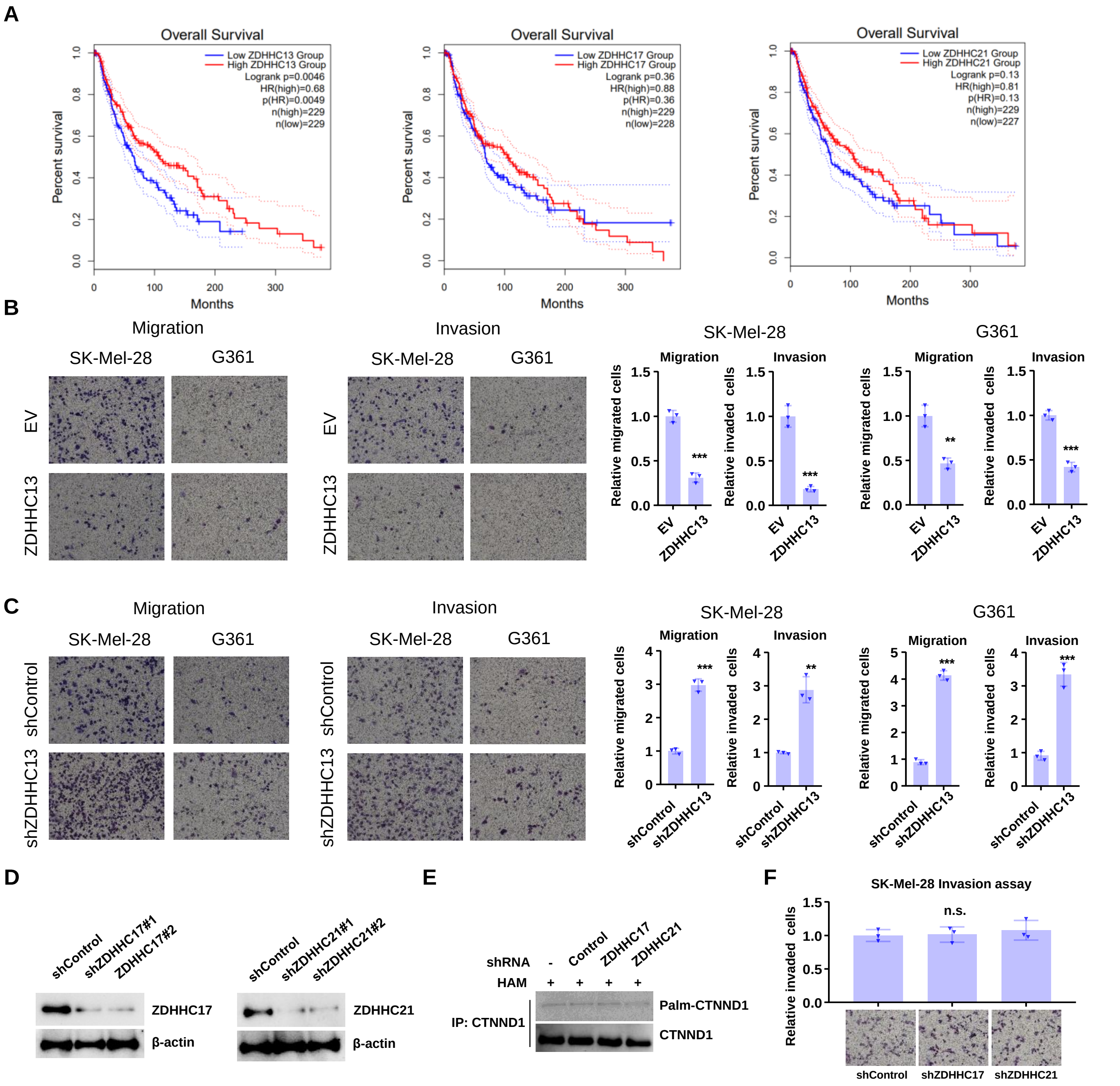


**Figure S4**

**(A)** Melanoma patient survival analysis based on ZDHHC expression (cutoff=50%) calculated by GEPIA2. All patients in the TCGA melanoma study were divided according to the expression level of ZDHHC13, 17 or 21. (higher or lower level than median expression value of all patients). **(B-C)** $4x10^3$ SK-Mel-28 or G361 cells stably expressing ZDHHC13 (B), or ZDHHC13 knockdown (C) with FBS-free medium were plated in the upper chamber of transwell filters (8 µm pore size) for 6 h (migration assay), or Matrigel coated transwells for 16 h (invasion assay). Three independent experiments were measured and calculated as mean ± SD, n=3. **(D)** Confirmation of ZDHHC17 and ZDHHC21 knockdown in SK-Mel-28 by Western Blot. Human shZDHHC17#1 and shZDHHC21#1 were selected for future experiments. **(E)** CTNND1 palmitoylation in SK-Mel-28 cells following ZDHHC17 or ZDHHC21 knockdown.SK-Mel-28 cells with stable knockdown of ZDHHC17 or ZDHHC21 were subjected to IP and ABE assays. CTNND1 palmitoylation was assessed by ABE. **(F)** $4x10^3$ SK-Mel-28 with stable knockdown of ZDHHC17 or ZDHHC21 with FBS-free medium were plated in the upper chamber of Matrigel coated transwells (8 µm pore size) for 16 h (invasion assay). Three independent experiments were measured and calculated as mean ± SD, n=3.

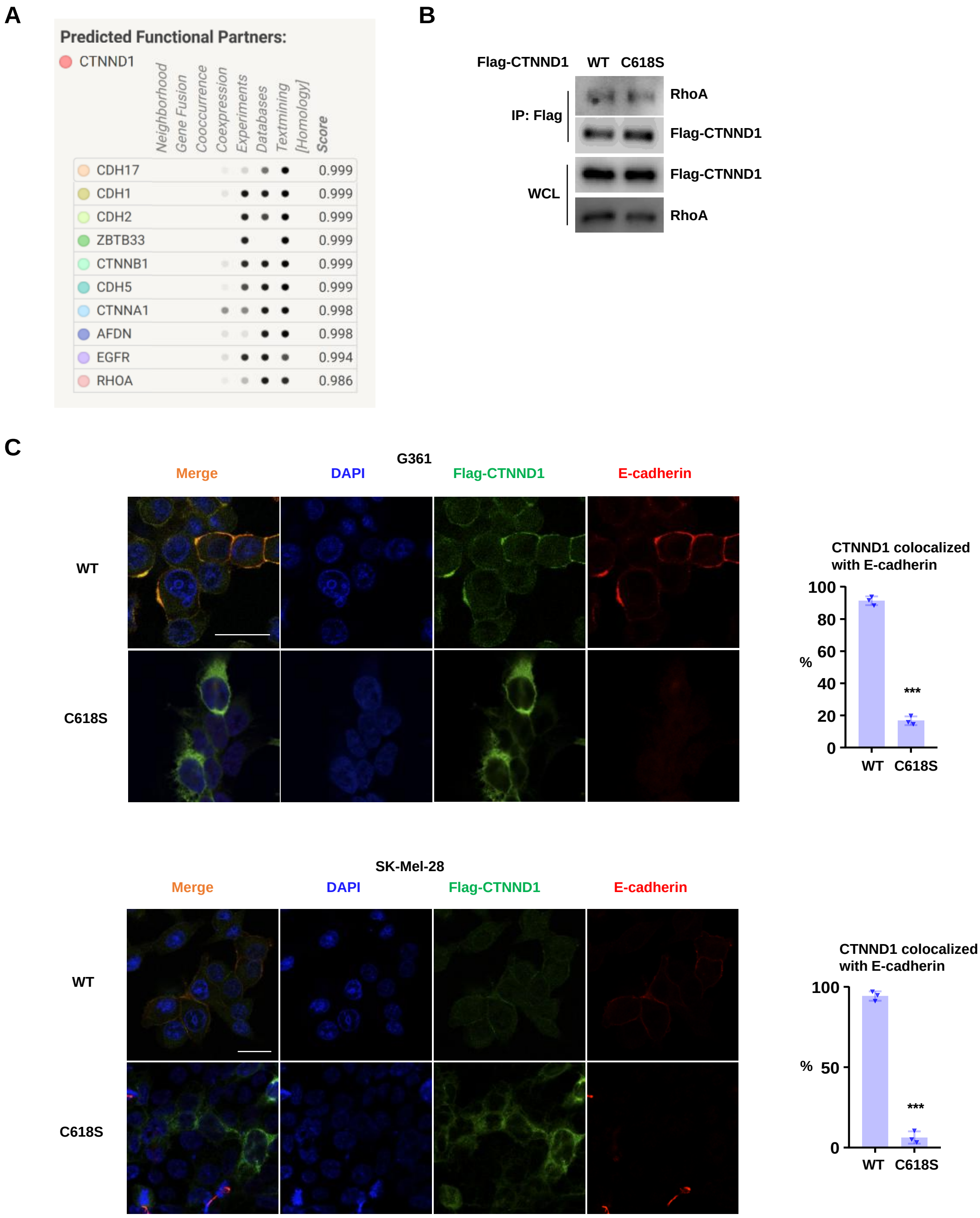


## Figure S5

**(A)** E-cadherin (CDH-1) interacting proteins in STRING database. **(B)** Interaction of WT and C619S CTNND1 with RhoA in SK-Mel-28 cells.SK-Mel-28 melanoma cells were transfected with WT or C618S Flag-tagged CTNND1. Cell lysates were subjected to immunoprecipitation using anti-Flag antibody, and co-immunoprecipitated RhoA was detected by Western blot. Expression of Flag-CTNND1 and RhoA in whole cell lysates (WCL) is also shown. **(C)** Confocal immunofluorescence imaging was performed on SK-Mel-28 cells expressing WT or C618S CTNND1. Colocalization analysis was performed using the Coloc 2 plugin in ImageJ (Fiji), providing quantitative assessment of CTNND1-Ecadherin colocalization. Scale bar: 25 µm. *p<0.05, **p<0.01, ***p<0.001, unpaired student's t-test.

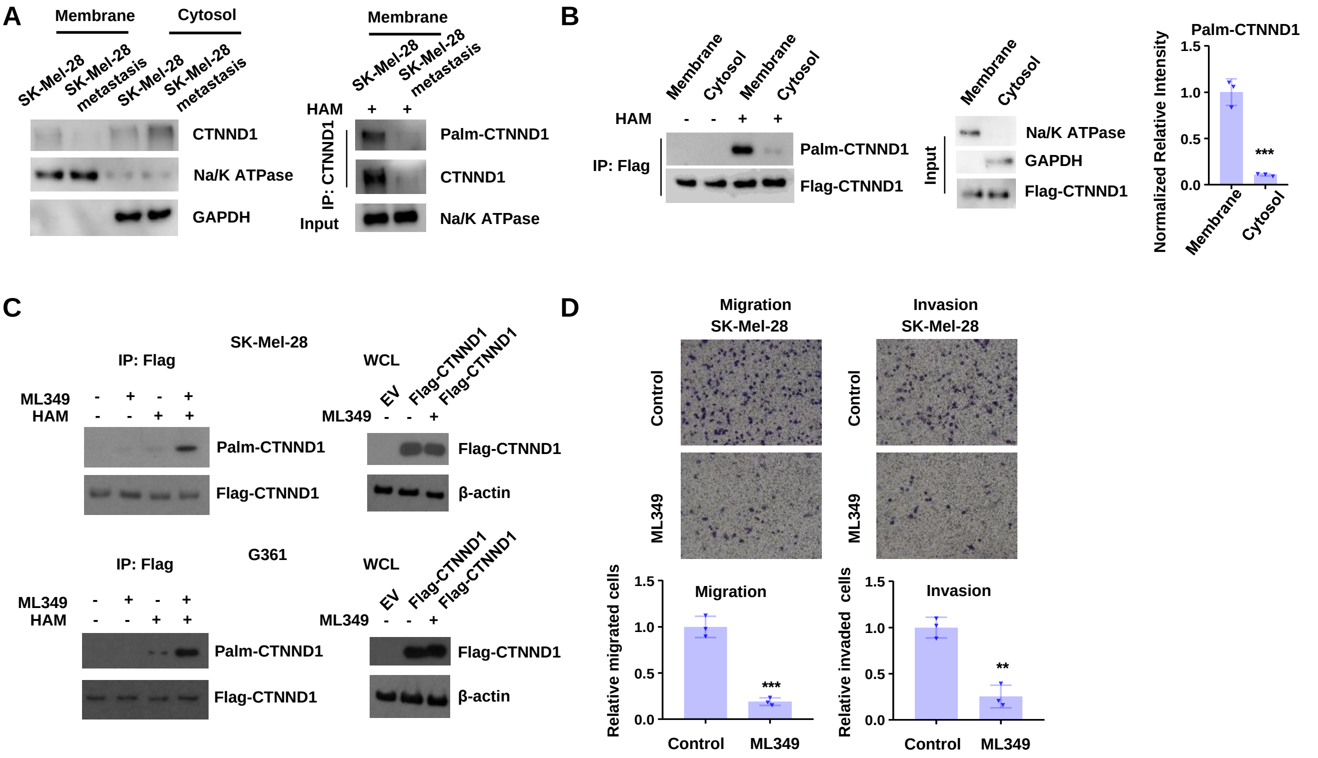


**Figure S6**

**(A)** Membrane and cytosolic protein fractions were isolated from SK-Mel-28 parental and metastatic cells using the Mem-PER Plus Membrane Protein Extraction Kit. Na/K ATPase and GAPDH served as membrane and cytosolic markers, respectively. Western blot and ABE assay were performed to detect CTNND1 and palmitoylated CTNND1 levels. **(B)** Membrane and cytosolic protein fractions were isolated from SK-Mel-28 cells using the Mem-PER Plus Membrane Protein Extraction Kit. Na/K ATPase and GAPDH were used as membrane and cytosolic markers, respectively. Western blotting and ABE assays were performed to assess CTNND1 expression and palmitoylation. Western blot band intensity were calculated by Image J software. Values are mean ± SD. **(C)** Exogenous Flag-CTNND1 is overexpressed in SK-Mel-28 cells or G361 cells by lentiviral infection, then cells were treated with 1μM ML349 for 24 h, then the cell lysates were collected for IP, IB and ABE analysis. **(D)** 4x$10^3$ SK-Mel-28 cells with FBS-free medium and 1μM ML349 were plated in the upper chamber of transwell filters (8 μm pore size) for 6 h (migration assay), or Matrigel coated transwells for 16 h (invasion assay). *p<0.05, **p<0.01, ***p<0.001, unpaired student's t-test.

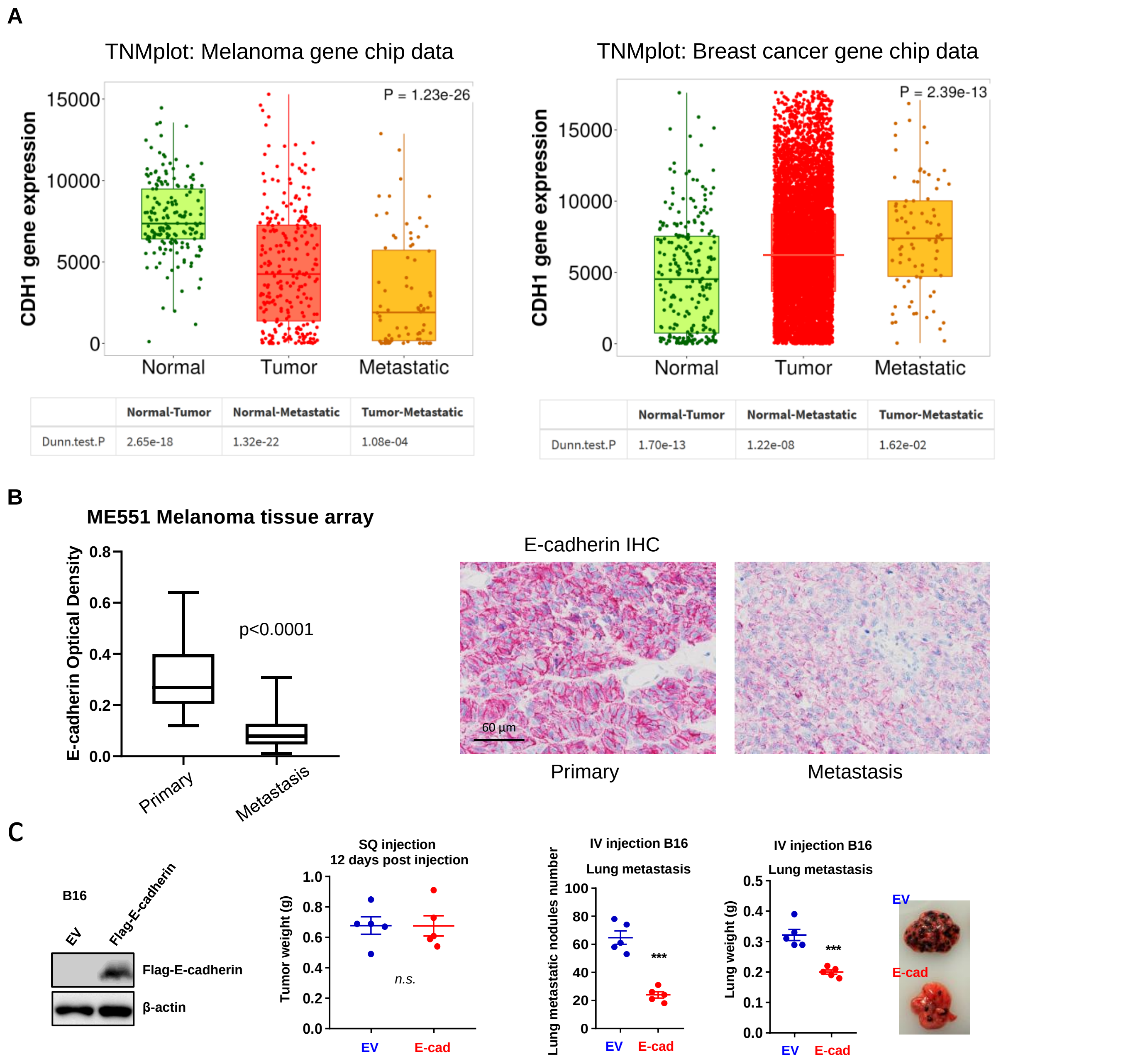


**Figure S7**

**(A)** E-cadherin gene expression comparison in human normal tissue, melanoma and metastatic melanoma samples from TNMplot melanoma gene chip dataset. **(B)** Immunohistochemistry was performed on a human melanoma tissue array (ME551) containing 27 primary and 22 metastatic samples using the Discovery ULTRA automated stainer (Roche). Sections were stained under optimized conditions and visualized using the OmniMap anti-Rabbit HRP system with Discovery Red. Slides were counterstained with hematoxylin. Optical density was quantified from bright-field images using ImageJ by color deconvolution (FastRed FastBlue) and thresholding. Statistical comparisons were made using unpaired two-tailed Student's t-test in GraphPad Prism. **(C)** $1X10^6$ B16 expressing EV or Flag-E-cadherin in 100ul PBS were subcutaneously injected into the flank of NOD scid mice. Tumor weight were measured 12 days after tumor cell injection. Error bars represent ±SD (n=5). $2\times10^5$ B16 expressing EV or Flag-E-cadherin in 100ul PBS were injected into NOD scid mice via the tail vein. Pulmonary metastases were assessed 14 days after tumor cell injection. Error bars represent ±SD (n=6). *p<0.05, **p<0.01, ***p<0.001, unpaired student's t-test.

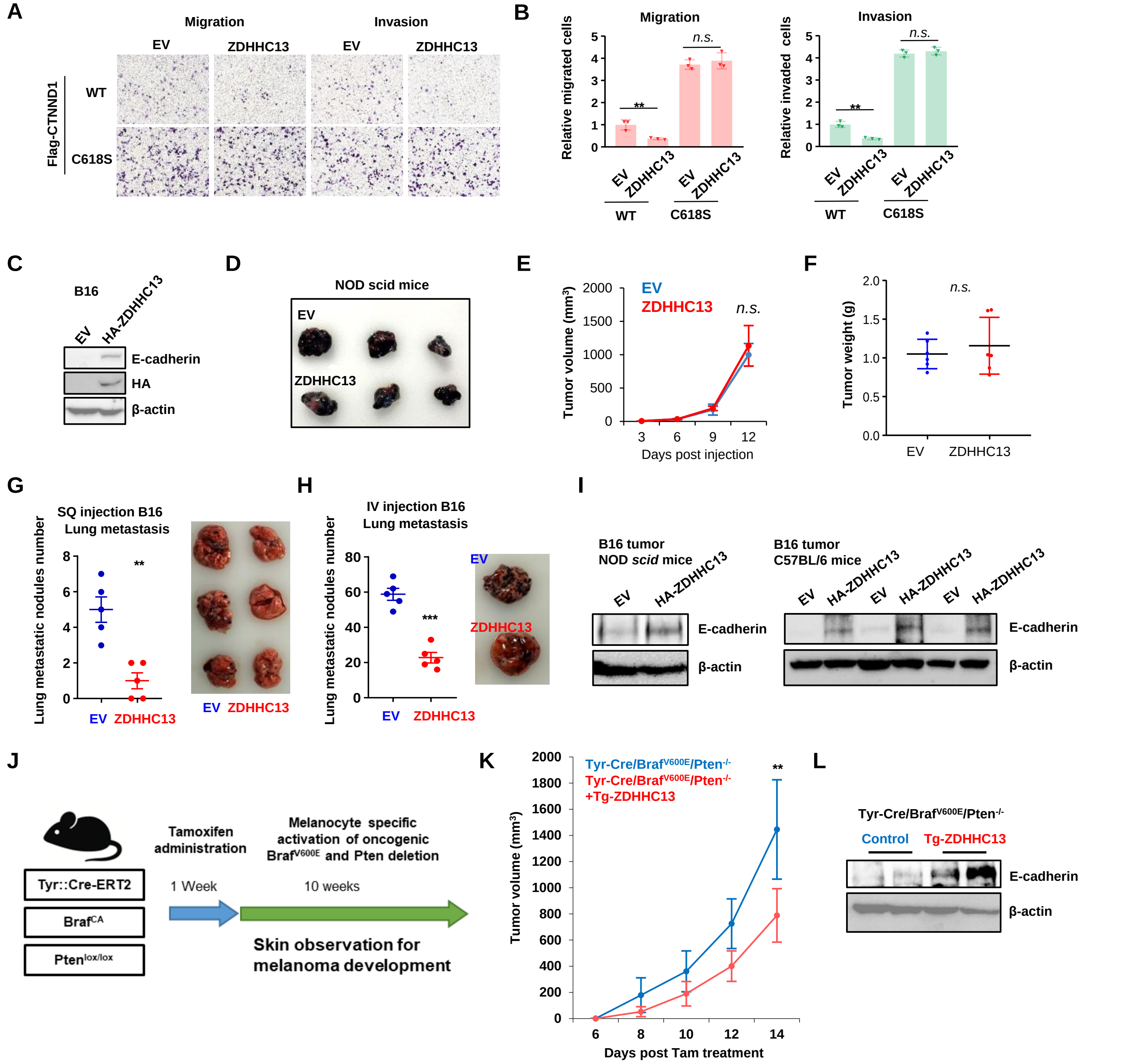


## Figure S8

**(A-B)** 4x10³ CTNND1-depleted SK-Mel-28 cells expressing WT, C618S CTNND1 or ZDHHC13 with FBS-free medium were plated in the upper chamber of transwell filters (8 μm pore size) for 6 h (migration assay), or Matrigel coated transwells for 16 h (invasion assay). Three independent experiments were measured and calculated as mean ± SD, n=3. **(C)** B16 overexpressing control empty vector (EV) and HA-ZDHHC13 were achieved by lentiviral infection. **(D-F)** 5X10⁵ B16 in 100ul PBS were subcutaneously injected into the shaved flank of NOD scid mice. Tumor growth **(E)** and weight **(F)** were measured. Error bars represent ±SD (n=6). **(G)** 1X10⁶ B16 expressing EV or HA-ZDHHC13 in 100ul PBS were subcutaneously injected into the flank of NOD scid mice. Pulmonary metastases were measured 12 days after tumor cell injection. Error bars represent ±SD (n=5). **(H)** 2×10⁵ B16 expressing EV or HA-ZDHHC13 in 100ul PBS were injected into NOD scid mice via the tail vein. Pulmonary metastases were assessed 14 days after tumor cell injection. Error bars represent ±SD (n=5). **(I)** Tumor samples collected in Fig. S8D and Fig. 4D were subjected to western blot analysis to quantify the protein level of E-cadherin. **(J)** Schematic for melanoma development procedure in mice in Fig. 4L-O. **(K)** Tumor growth in Tyr-Cre/BrafV600E/Pten-/- and Tyr-Cre/BrafV600E/Pten-/- +Tg-ZDHHC13 mice after Tam injection. Error bars represent ±SD, n=5. **(L)** Tumors from Tyr-Cre/BrafV600E/Pten-/- and Tyr-Cre/BrafV600E/Pten-/- +Tg-ZDHHC13 mouse were collected for Western Blot analysis to detect E-cadherin expression. *p<0.05, **p<0.01, ***p<0.001, unpaired student's t-test.

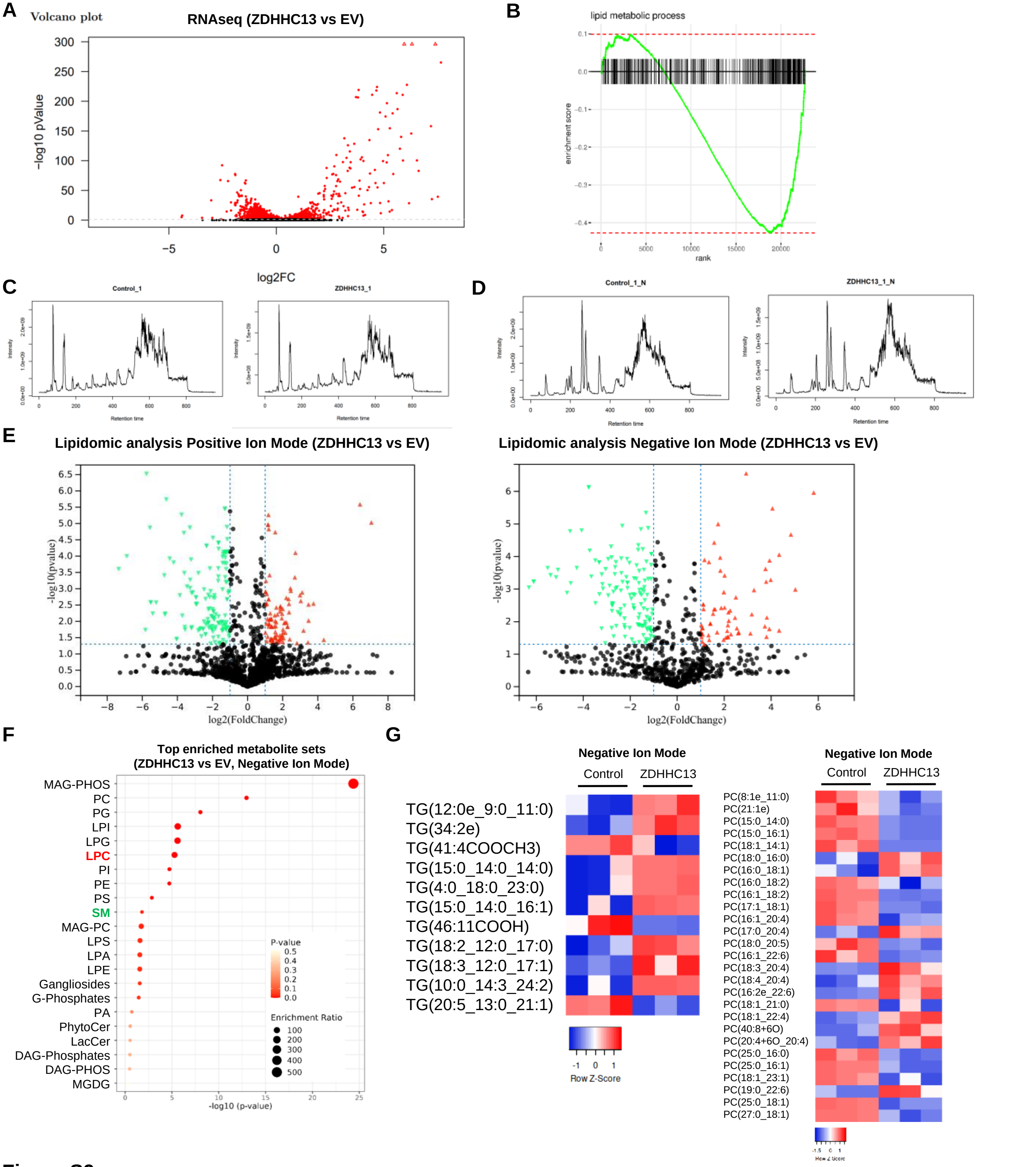


**Figure S9**

**(A-B)** RNAseq analysis of B16 cells and B16 cells stably expressing ZDHHC13. The differentiated gene expression and GSEA for GO enrichment were performed by using RaNA-seq (rana-seq.eu). **(C)** Total ions chromatogragh (TIC) chromatograms of the sample ESI+ mode (n=3). **(D)** Total ions chromatogragh (TIC) chromatograms of the sample ESI- mode (n=3). **(E)** Univariate analysis including fold change analysis and t-test were performed on volcano plot. Lipid metabolites with a variable importance in the projection (VIP) score>1, Fold Change>2.0 and p<0.05 were considered significant changed lipids. **(F)** Pathway enrichment analysis of lipids based on KEGG database and MetaboAnalyst. Lipid metabolites with a variable importance in the projection (VIP) score>1, Fold Change>2.0 and p<0.05 were considered significant changed lipids (Negative Ion Mode). **(G)** Heat map of each species in TG and PC based on their intensity in B16 cells (Negative Ion Mode).

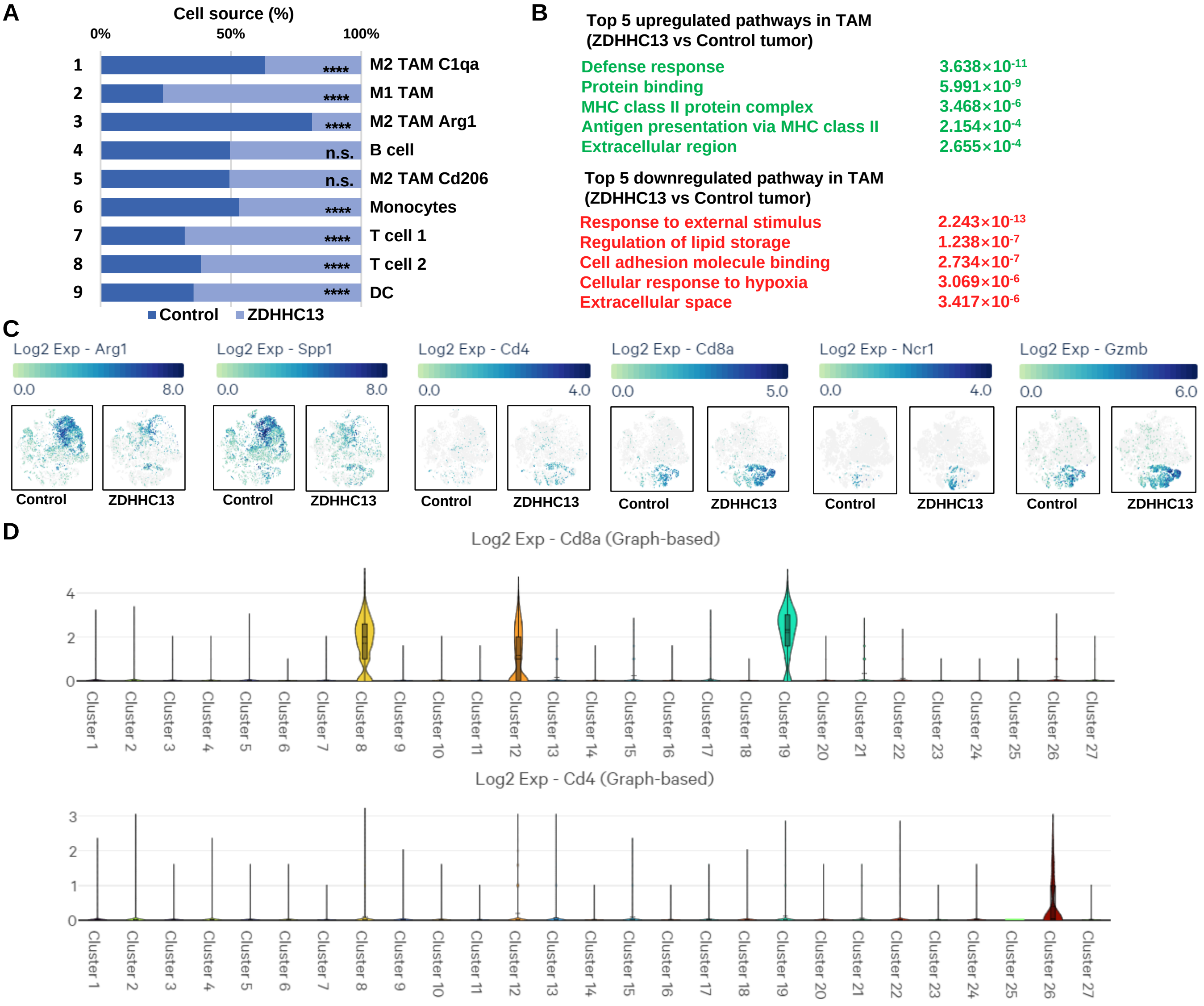


**Figure S10**

**(A)** Percentage of cells from each genotype within different clusters. Clusters exhibiting a notable difference are indicated. ****p<0.0001 (hypergeometric test). **(B)** Analysis of differentiated expressed genes in TAMs (cluster 1, 2 and 3). The differentiated expressed genes were identified by Loupe and the pathway analysis was performed by using g:Profiler. **(C)** Gene counts of M2 TAM markers-Arg1, Spp1, T cell markers-Cd4, Cd8a, NK cell marker-Ncr1, and CTL and NK cell marker Gzmb in 10x single cell data set. **(D)** A graph-based clustering approach was applied, constructing a k-nearest neighbor graph and identifying cell communities via the Louvain algorithm. The analysis resolved 27 distinct clusters, clearly separating CD4$^{+}$ and CD8a$^{+}$ T cell populations, consistent with established immunological identities.

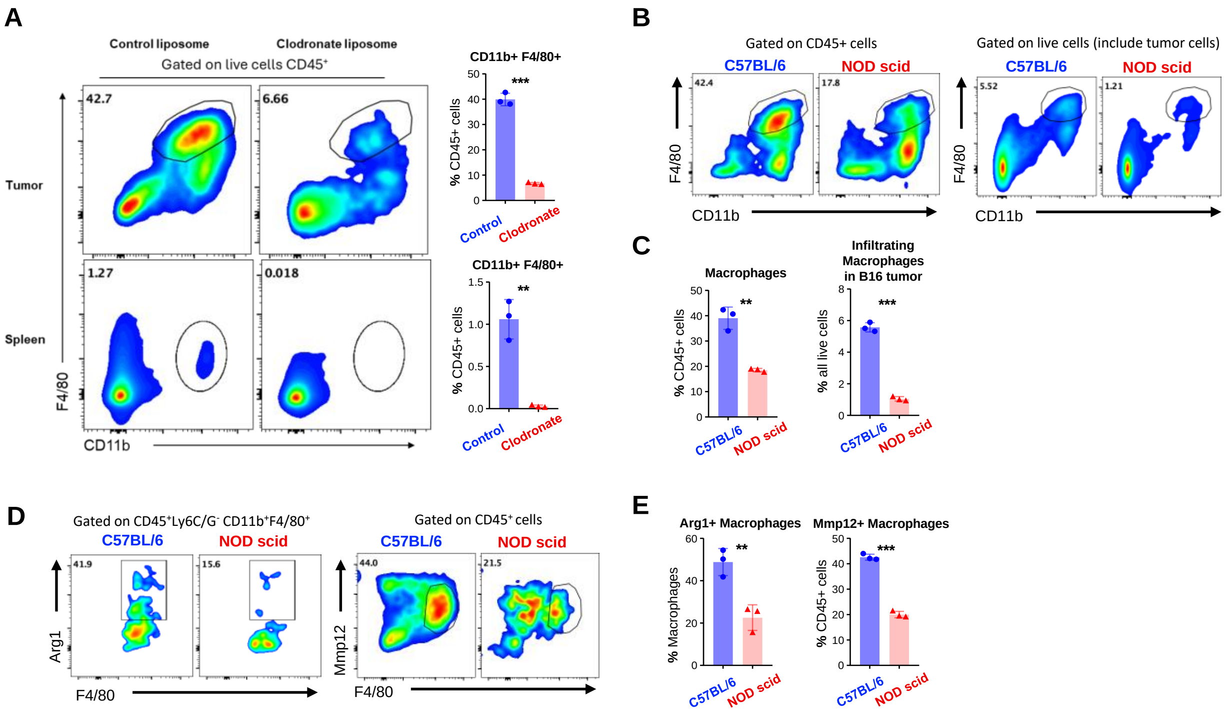


**F** Examples of Signature enrichment genes in Cluster 3 (M2 TAM Arg1)

| Gene | Log2FC | p-Value |
|---|---|---|
| Mmp12 | 4.19 | 3.12e-73 |
| Mmp13 | 3.16 | 7.85e-39 |
| Arg1 | 2.97 | 1.90e-34 |
| Spp1 | 2.82 | 3.45e-30 |
| Vegfa | 2.31 | 7.09e-20 |
| Pla2g7 | 1.27 | 1.09e-5 |
| Cd274 | 1.04 | 5.42e-4 |
| Mmp14 | 0.88 | 5.84e-3 |

**G** Examples of Differential expressed genes in TAMs (ZDHHC13 vs Control tumor)

| Gene | Log2FC | p-Value |
|---|---|---|
| Mmp13 | -3.44 | 6.42e-35 |
| Mmp12 | -3.07 | 1.78e-29 |
| Spp1 | -1.94 | 1.65e-12 |
| Arg1 | -0.99 | 6.45e-4 |
| Pla2g7 | -0.81 | 8.46e-3 |

## Figure S11

**(A)** Macrophages were depleted in C57BL/6 mice through IV injection of clodronate liposomes, given two days before tumor inoculation. Three injections will be administered with three-day intervals between each dose to maintain macrophage depletion. Mice were injected with $1\times10^6$ B16 melanoma cells. The depletion of macrophages in both tumor and spleen were confirmed by flow cytometry. **(B-E)** $5\times10^5$ B16F10 in 100ul PBS were subcutaneously injected into the shaved flank of C57BL/6 mice and NOD scid mice. When tumor size reached ~$2cm^3$, the tumor were subjected for flow cytometry analysis using macrophage lineage markers **(B-C)** and M2-like TAM markers Arg1 and Mmp12 **(D-E)**. **(F)** Examples of signature gene enrichment in cluster 3 (M2 TAM Arg1). The Log2FC is the ratio of the normalized mean gene UMI counts in the cluster relative to all other clusters. **(G)** Examples of differential expressed genes in all TAMs (Cluster 1, 2 and 3) from 10x single cell data set (Control vs ZDHHC13).

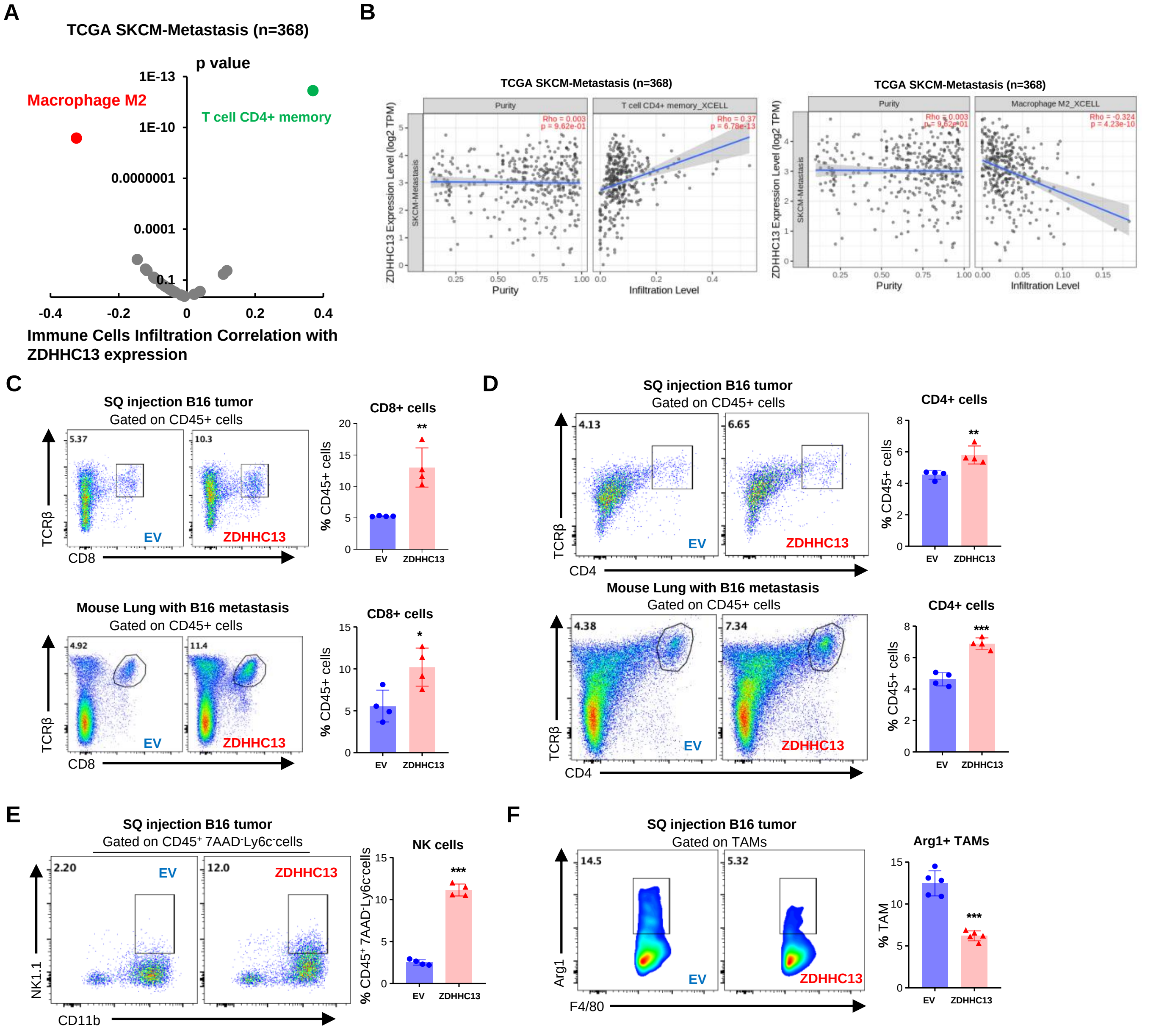


**Figure S12**

**(A-B)** Quantification of the tumor Immune contexture from TCGA SKCM metastatic melanoma patients by xCell tool (timer.cistrome.org). These include T cell, B cell, NK cell, macrophage, DC, Neutrophil and their subsets. **(C-F)** Tumor cells or mouse lungs with melanoma metastasis were isolated for flow cytometry analysis. Error bars represent ±SD. **(C)** CD8+ T cells (n=4); **(D)** CD4+ T cells (n=4); **(E)** NK cells (n=4); **(F)** Arg1+ macrophages (n=5). *p<0.05, **p<0.01, ***p<0.001, unpaired student's t-test.

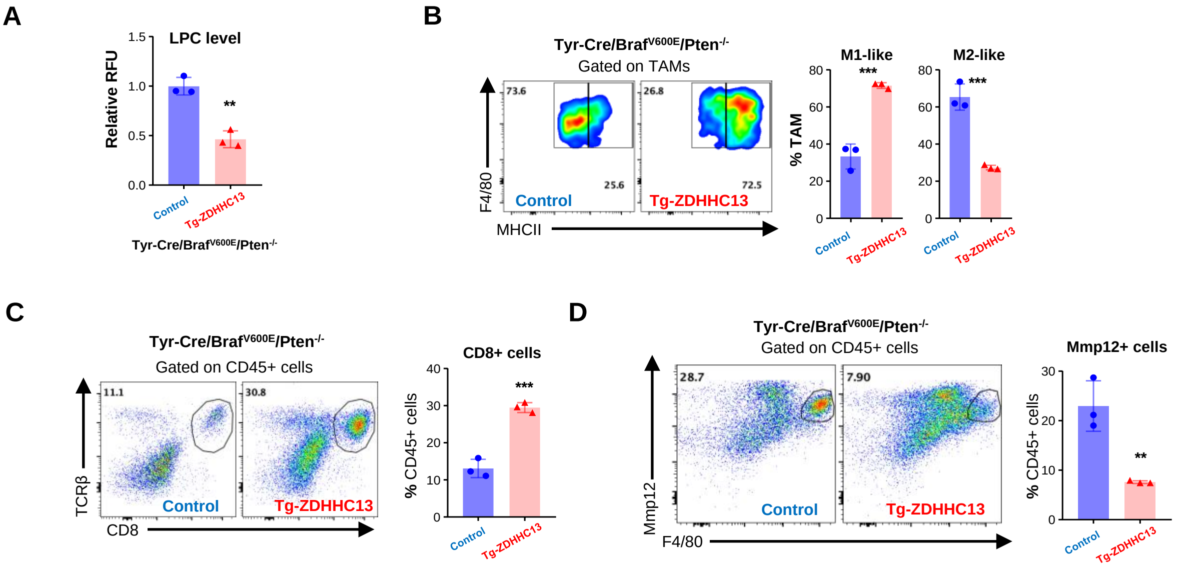


## Figure S13

**(A)** Tumors from Tyr-Cre/BrafV600E/Pten-/- and Tyr-Cre/BrafV600E/Pten-/- +Tg-ZDHHC13 mouse were collected for LPC quantification by using Abcam SM and LPC assay kit (Abcam ab138877 and ab273332), respectively. **(B-D)** Cells in tumors from Tyr-Cre/BrafV600E/Pten-/- and Tyr-Cre/BrafV600E/Pten-/- +Tg-ZDHHC13 mouse were isolated for flow cytometry analysis. Error bars represent ±SD. Flow cytometry analysis of tumor-infiltrating immune cells gating on TAMs to show M1-like and M2-like macrophages (n=3); CD8+ T cells (n=3); and Mmp12+ macrophages (n=3).

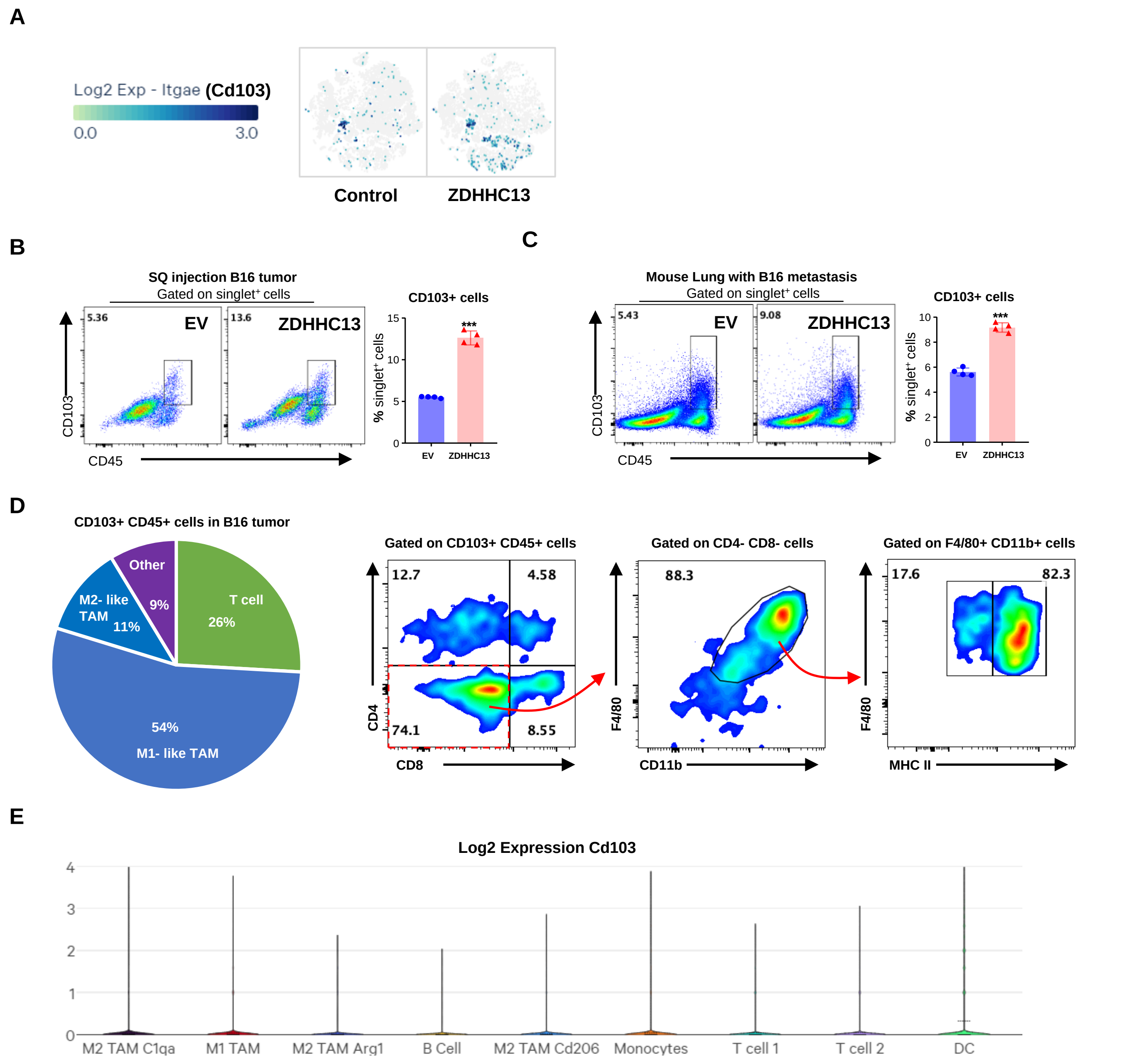


**Figure S14**

**(A)** Gene counts of CD103 in 10x single cell data set. **(B)** Flow cytometry analysis of infiltrating CD103+ immune cells in B16 tumors **(B)** or mouse lungs with B16 metastasis **(C)**. **(D)** CD103$^{+}$CD45$^{+}$ cells were isolated from B16 tumors and analyzed by flow cytometry. Cells were sequentially stained for CD4 and CD8, macrophage markers CD11b and F4/80, and the M1-like marker MHC class II (MHCII). **(E)** Expression of Cd103 across all cell clusters.

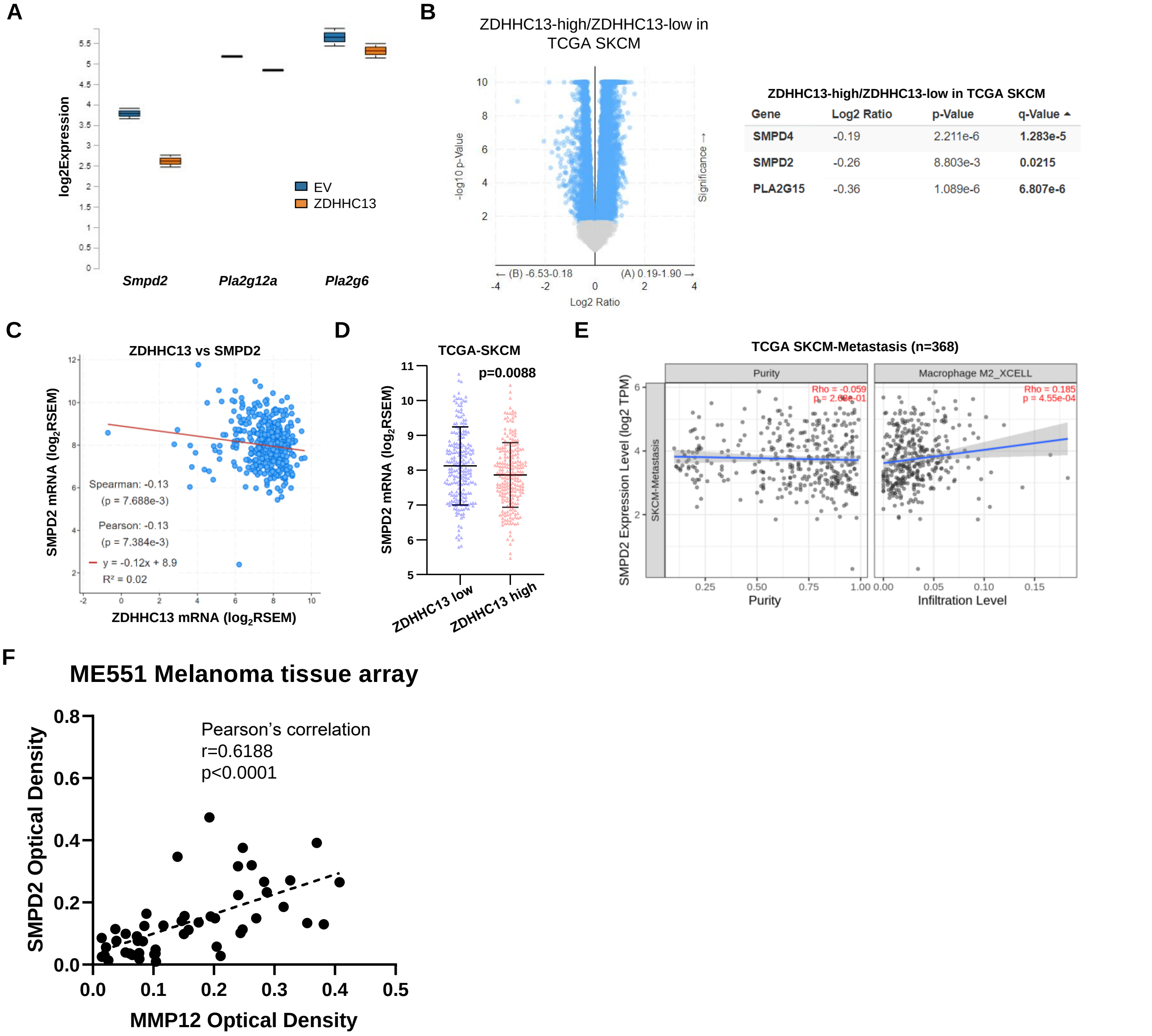


**Figure S15**

**(A)** RNAseq analysis of B16 cells and B16 cells stably expressing ZDHHC13. The differentiated gene expression was performed by using RaNA-seq (rana-seq.eu). The genes related to LPC and SM metabolism were listed. **(B)** Differential gene expression analysis in ZDHHC13-low expression group and ZDHHC13-high expression group (separated by the median ZDHHC13 expression) in SKCM (TCGA, PanCancer Atlas) data set. Samples with values above the median were classified into the "high" group, and those below into the "low" group. **(C)** The correlation between ZDHHC13 and SMPD2 mRNA expression in TCGA SKCM, calculated by CBioPortal. **(D)** Comparison of SMPD2 mRNA expression in ZDHHC13-low expression group and ZDHHC13-high expression group (separated by the median ZDHHC13 expression) in SKCM (TCGA, PanCancer Atlas) data set. **(E)** Correlation of the tumor-infiltrating M2-like TAMs and SMPD2 expression from TCGA SKCM metastatic melanoma patients by xCell tool (timer.cistrome.org). **(F)** Immunohistochemistry was performed on a human melanoma tissue array (ME551) containing 27 primary and 22 metastatic samples using the Discovery ULTRA automated stainer (Roche). Sections were stained under optimized conditions and visualized using the OmniMap anti-Rabbit HRP system with Discovery Red. Slides were counterstained with hematoxylin. Optical density was quantified from bright-field images using ImageJ by color deconvolution (FastRed FastBlue) and thresholding. Statistical comparisons were made using Pearson's correlation in GraphPad Prism.

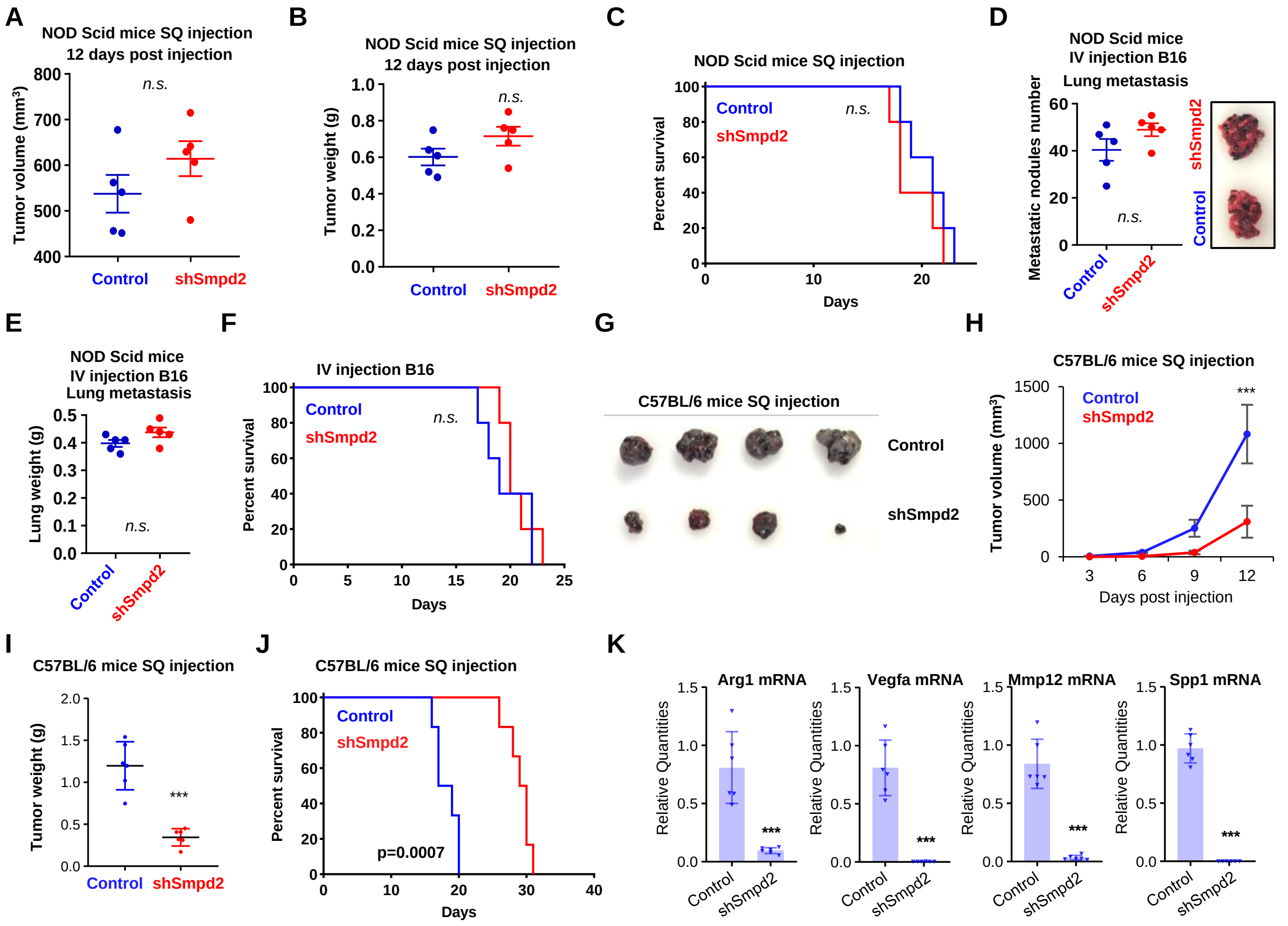


## Figure S16

**(AC)** 5X10$^5$ B16 and Smpd2-depleted B16 in 100ul PBS were subcutaneously injected into the flank of NOD scid mice. Tumor volume **(A)**, weight **(B)** and survival **(C)** were recorded. Error bars represent ±SD (n=5). **(D-F)** 2X10$^5$ B16 and Smpd2-depleted B16 in 100ul PBS were injected into NOD scid mice via the tail vein. Pulmonary metastases **(D-E)** were assessed 14 days after tumor cell injection and mice survival was recorded **(F)**. Error bars represent ±SD (n=5). **(G-J)** 1X10$^6$ B16 or Smpd2-depleted B16 in 100ul PBS were subcutaneously injected into the shaved flank of C57BL/6 mice. Tumor growth **(G-H)**, weight **(I)** and mouse survival **(J)** were assessed. Error bars represent ±SD (n=6). **(K)** Mouse lungs collected in experiment described in Fig. 7E were subjected for qRT-PCR analysis. *p<0.05, **p<0.01, ***p<0.001, unpaired student's t-test.

**A**

ZDHHC13 binding peptide spectral count in SK-Mel-28

| Protein | Unique Pep | LFQ intensity |
|---|---|---|
| PPP1R12A | 15 | 4604.884 |
| SMC3 | 15 | 2794.4832 |
| RBM25 | 14 | 4250.163 |
| HCFC1 | 13 | 3617.112 |
| GNL2 | 11 | 7859.0366 |
| RPS27A | 11 | 85628.95 |
| CTNND1 | 10 | 3681.023 |
| NOM1 | 10 | 2703.0493 |
| L3MBTL3 | 9 | 3537.9414 |
| LRPPRC | 9 | 2404.08 |
| DDX21 | 9 | 3375.7568 |
| OSBPL6 | 9 | 3564.9688 |
| PSAT1 | 9 | 2465.7097 |
| PLS3 | 9 | 6411.3755 |
| DDX56 | 9 | 1740.5222 |
| SDHA | 9 | 2739.1333 |

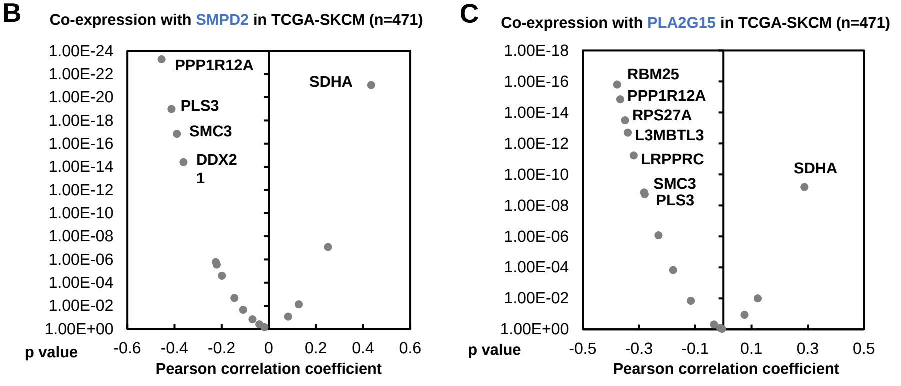


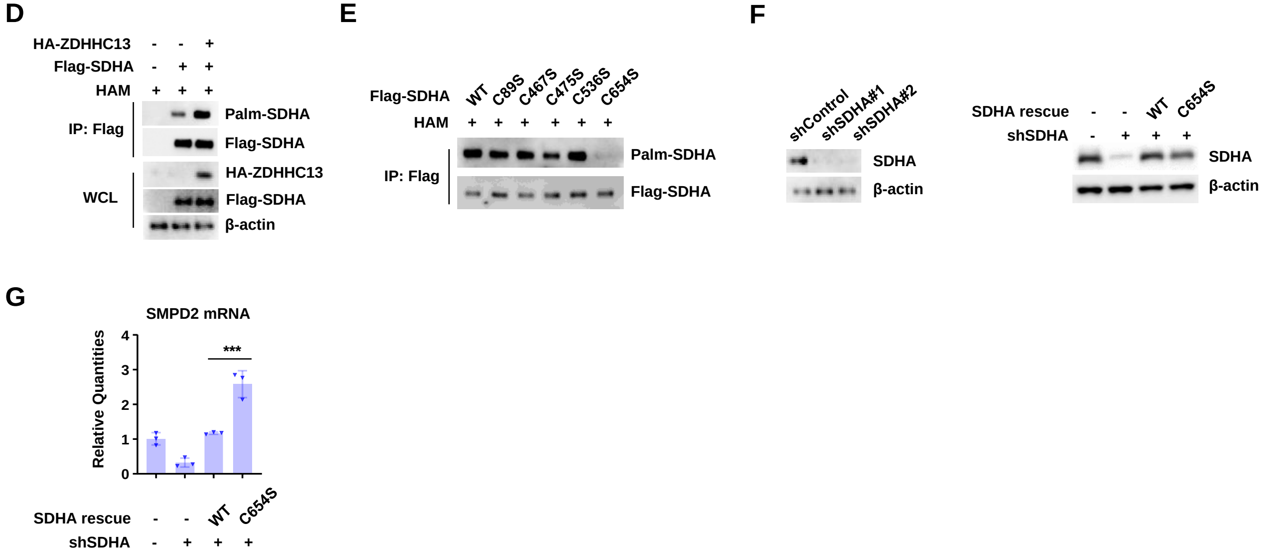


## Figure S17

**(A)** List of most abundant ZDHHC13-associated proteins identified by mass spectrometry in melanoma cells. **(B)** The correlation between SMPD2 and ZDHHC13 potential substrates mRNA expression in TCGA SKCM, calculated by CBioPortal. **(C)** The correlation between PLA2G15 and ZDHHC13 potential substrates mRNA expression in TCGA SKCM, calculated by CBioPortal. **(D)** Flag-tagged SDHA is palmitoylated in SK-Mel-28 cells, and this palmitoylation is enhanced by ZDHHC13, as detected by ABE assay. **(E)** ABE assay reveals that SDHA is predominantly palmitoylated at cysteine 654 (C654). **(F)** Knockdown of SDHA in SK-Mel-28 cells was confirmed by Western blot. Cells were subsequently rescued with WT or C654S mutant SDHA. **(G)** qRT-PCR analysis of SMPD2 mRNA expression in SDHA knockdown cells rescued with WT or C654S SDHA. $*p<0.05$, $**p<0.01$, $***p<0.001$, group comparison was performed using Dunnett's test.

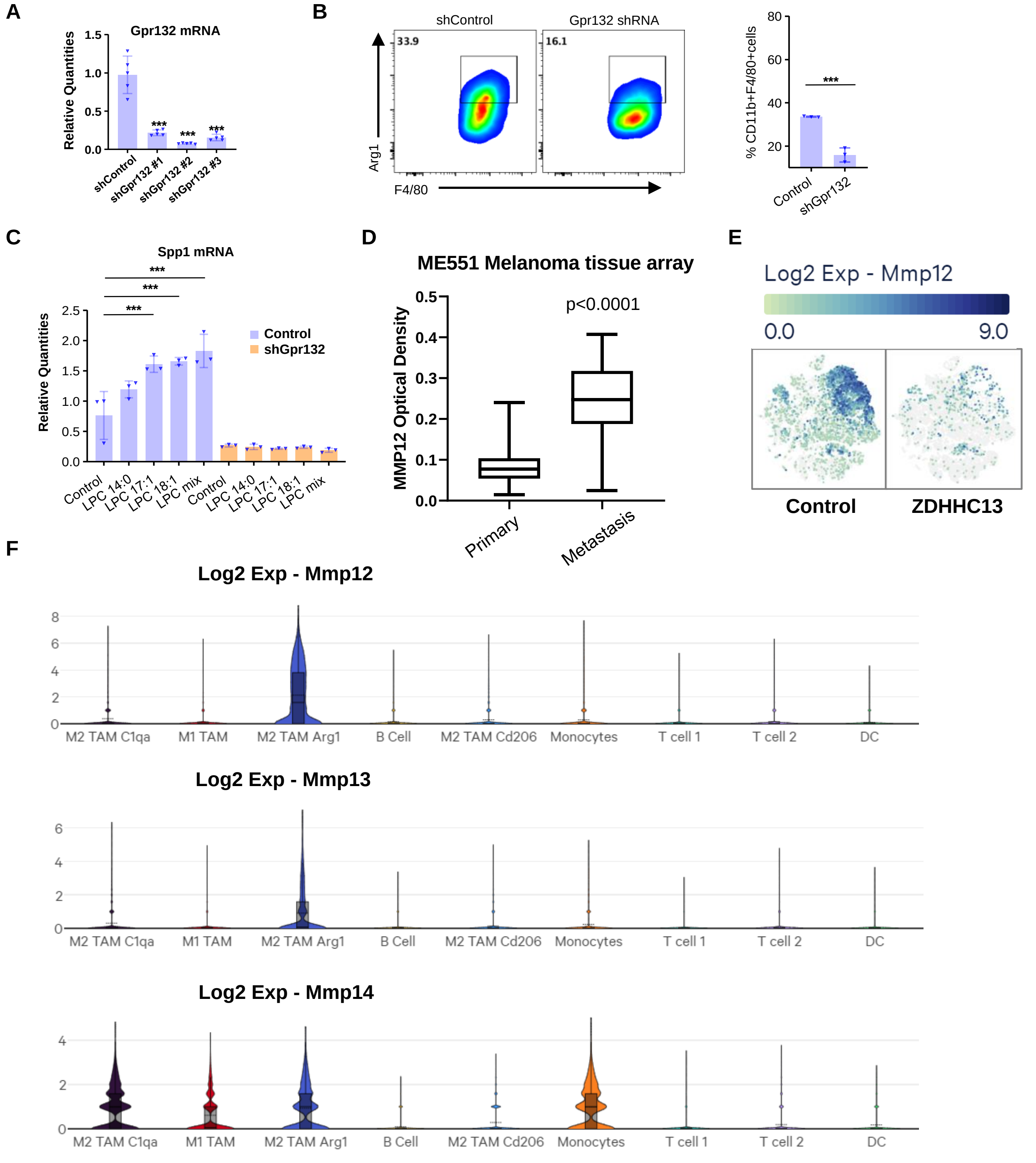


**Figure S18**

**(A)** Confirmation of Gpr132 knockdown in raw 264.7 macrophages by using qRT-PCR (n=3). **(B)** Raw 264.7 macrophages were pre-treated with IL-4, TGFβ and IL-10 mix (10ng/ml) for 24 h to induced M2-like polarization. Flow cytometry was used to detect Arg1+/F4/80+ M2 macrophages (n=3)**. (C)** Raw 264.7 macrophages were treated the same as in Fig. 8A-B, then total RNA samples were collected for qRT-PCR analysis (n=3). **(D)** Immunohistochemistry was performed on a human melanoma tissue array (ME551) containing 27 primary and 22 metastatic samples using the Discovery ULTRA automated stainer (Roche). Statistical comparisons were made using unpaired two-tailed Student's t-test in GraphPad Prism. **(E)** Gene counts of Mmp12 in 10x single cell data set. **(F)** Expression of distributions of Mmp12, Mmp13 and Mmp14 for different clusters in 10x single cell data set. *p<0.05, **p<0.01, ***p<0.001, group comparisons in **(A)** and **(C)** were performed using Dunnett's test, others were tested by unpaired student's t-test.

A

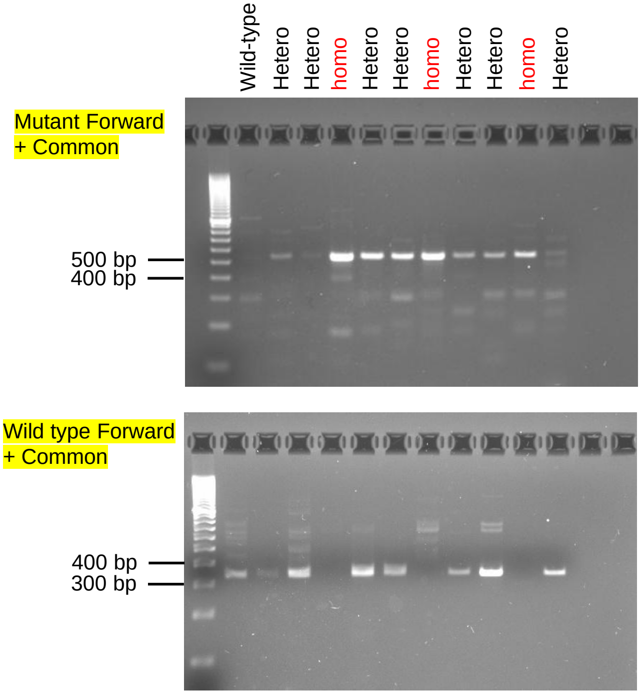


| Primer Type | SEQUENCE 5' → 3' |
|---|---|
| Mutant Forward | GCTACTTCCATTTGTCACGTCC |
| Wild type Forward | ACTGGGCAACTGGACAACTC |
| Common | CTCCCATGTGCTGGGATT AC |

B

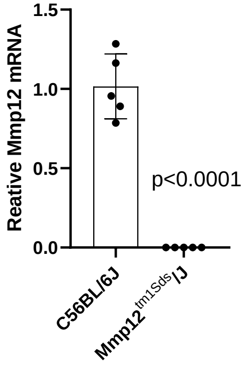


**Figure S19**

**(A)** B6.129X-Mmp12$^{tm1Sds}$/J (Stock No: 004855) mice were purchased from The Jackson Laboratory. Genotyping was performed according to the protocol 26467 provided by The Jackson Laboratory. Two sets of primers were used: one for the wild-type allele (Wild type Forward + Common) and one for the mutant allele (Mutant Forward + Common). The wild-type allele yields a band of approximately 460 bp, and the mutant allele produces a band around 350 bp. Wild-type mice display only the 460 bp band; homozygous mutant (Mut) mice show only the 350 bp band; heterozygous (Het) mice exhibit both bands. **(B)** Bone marrow derived macrophages (BMDMs) from C56BL/6J and Mmp12$^{tm1Sds}$/J mice were collected for qRT-PCR analysis of MMP12 mRNA expression.
