# Supplementary material for: S-acyl transferase ZDHHC13 modulates tumor microenvironment interactions to suppress metastasis in melanoma models
Source: J Clin Invest. 2025 Sep 30;135(23):e188249. doi: 10.1172/JCI188249 (PMC12646662; doi:10.1172/JCI188249)

Figure 1

D

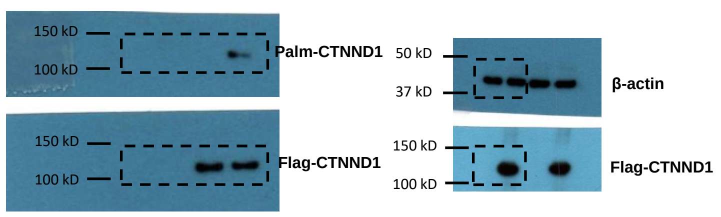

E

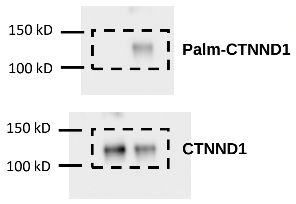

G

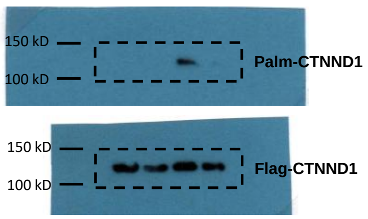

Figure 2

A

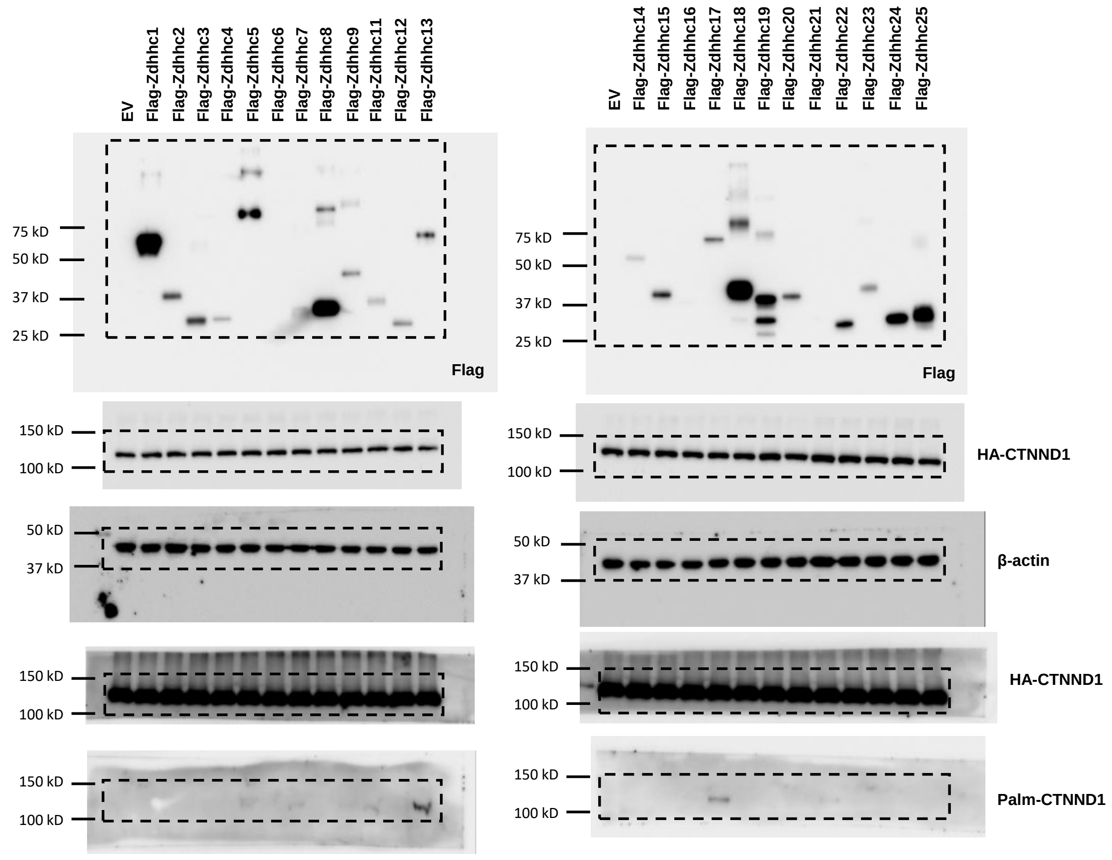

B

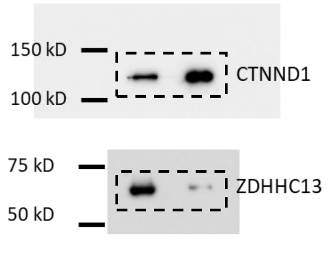

C

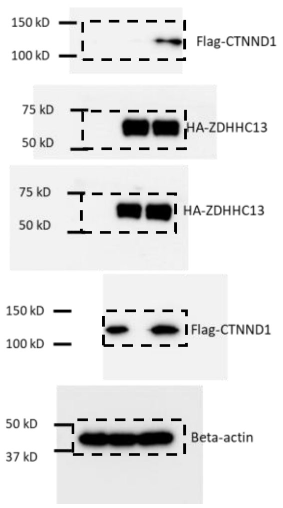

D

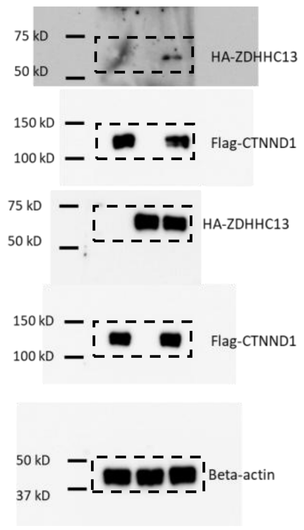

Figure 2

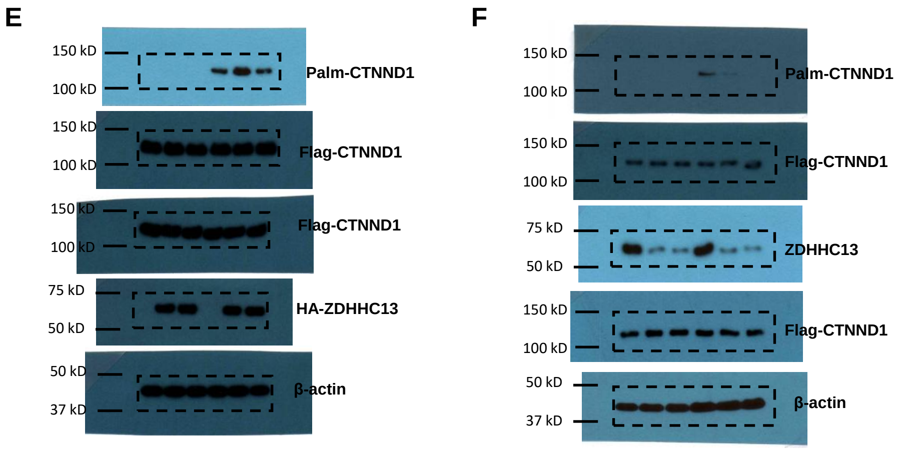

Figure 3

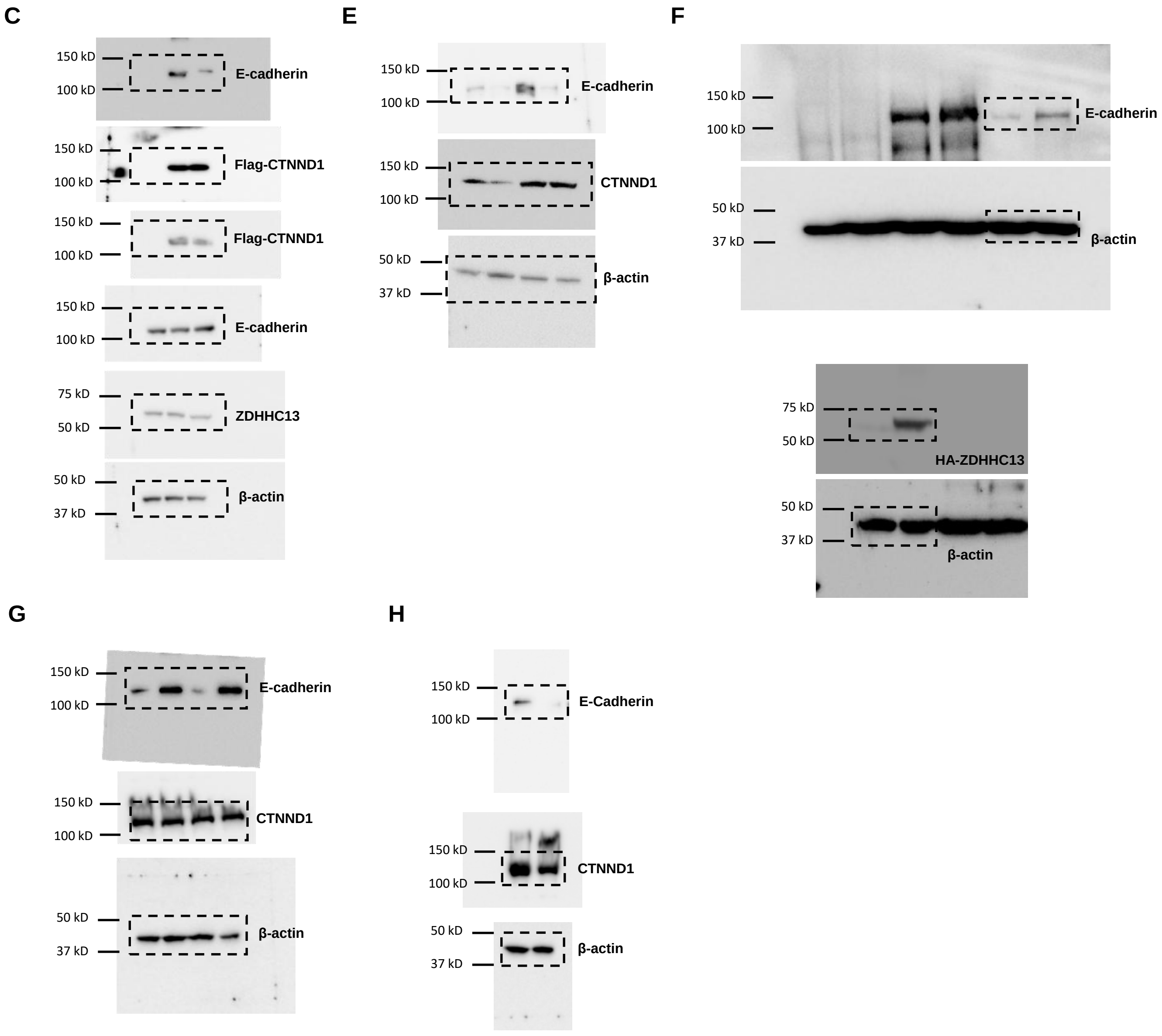

Figure 7

D

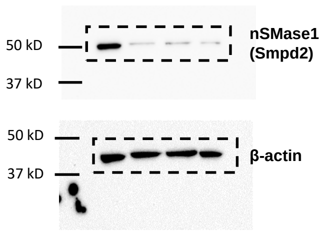

Figure 8

E

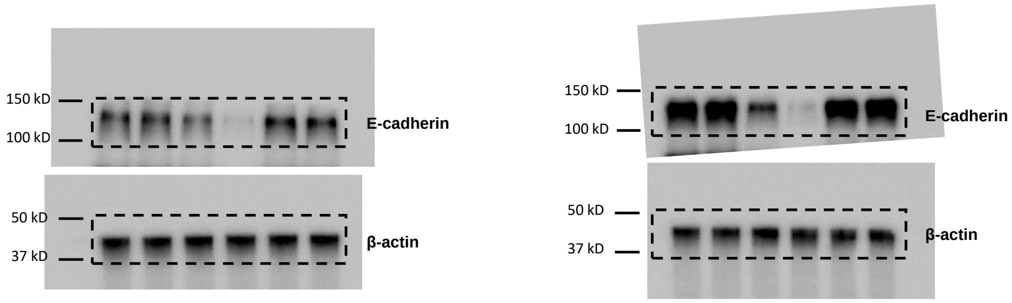

Figure S1

A

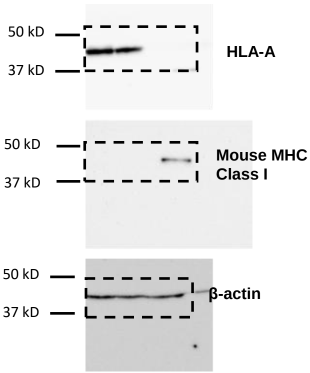

D

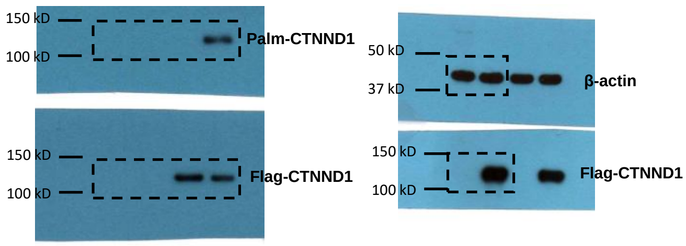

F

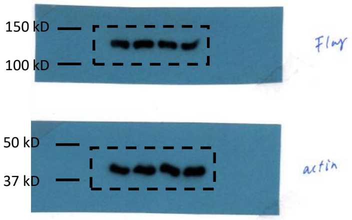

G

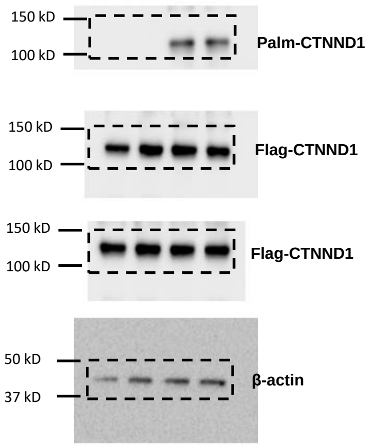

H

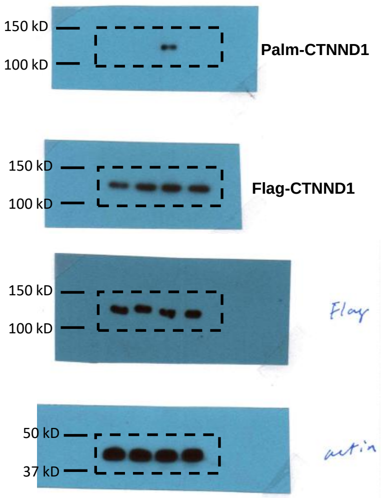

Figure S2

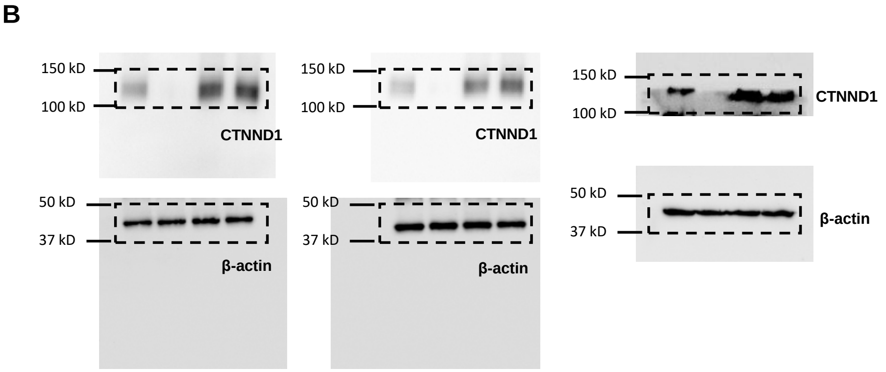

Figure S3

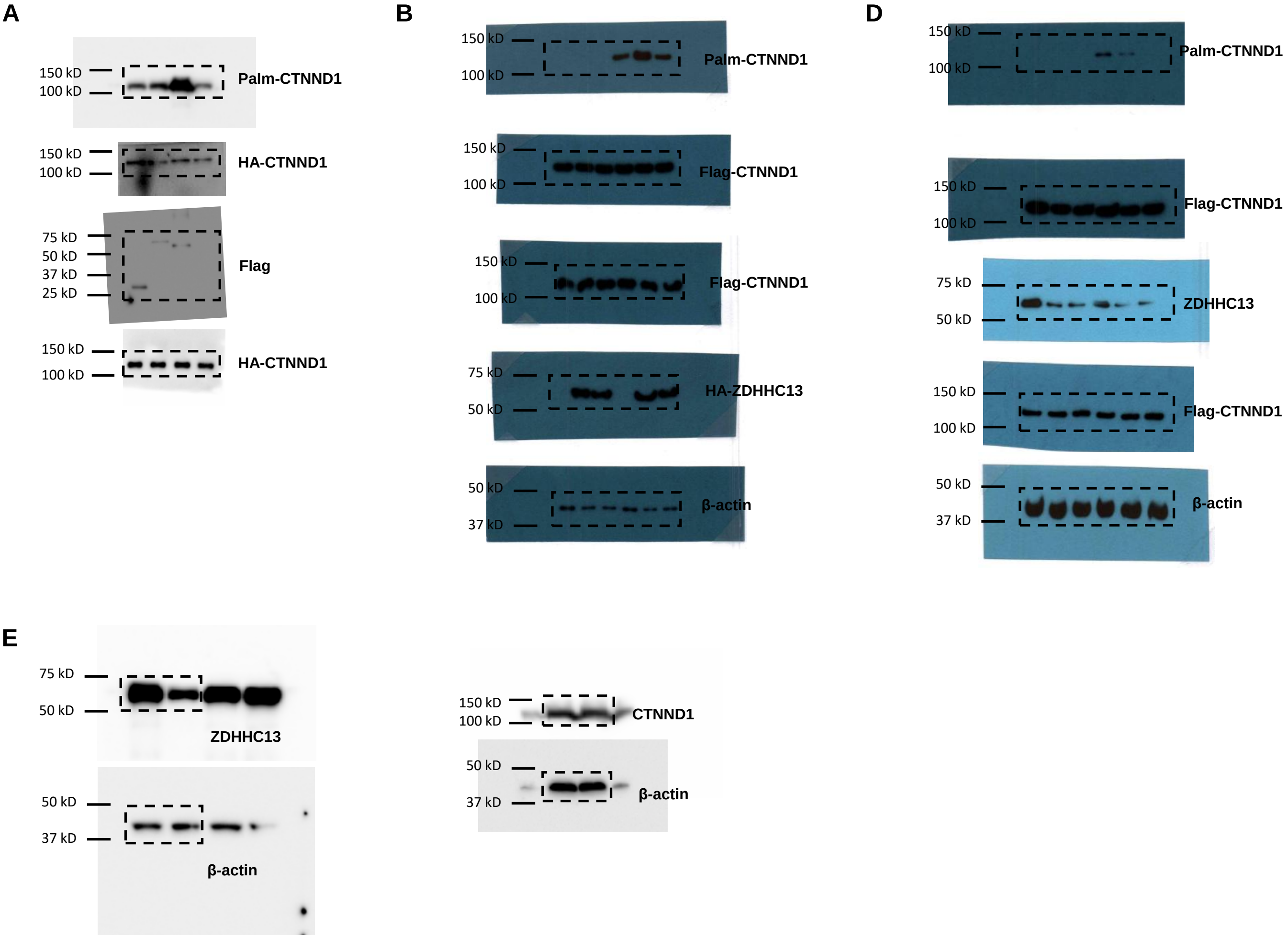

Figure S4

D

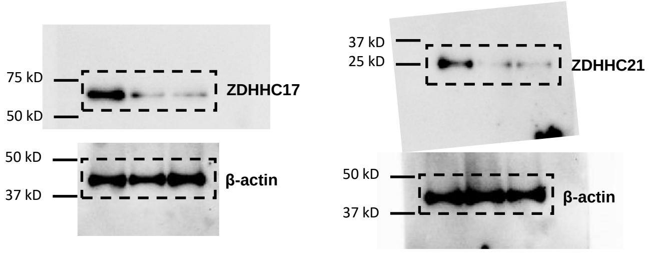

E

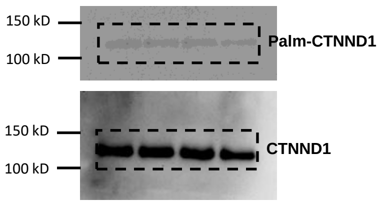

Figure S5

B

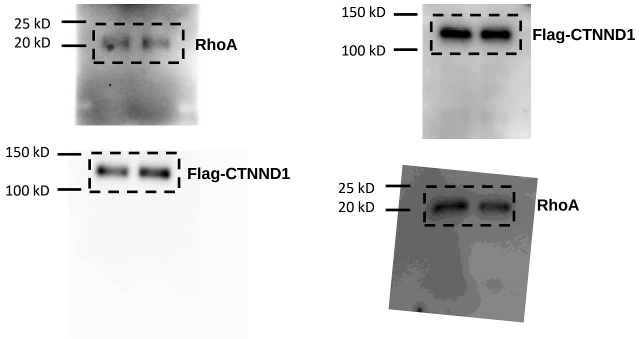

Figure S6

A

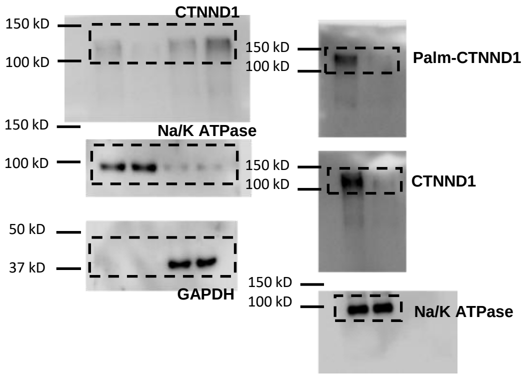

B

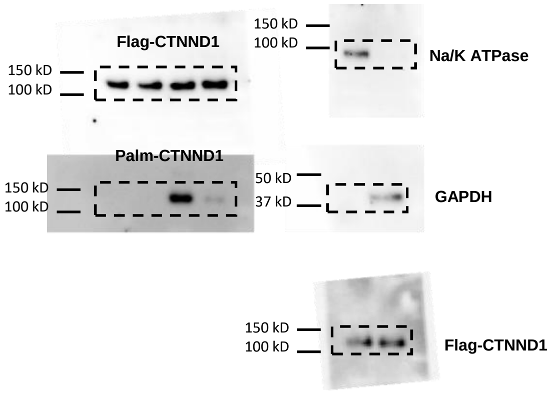

C

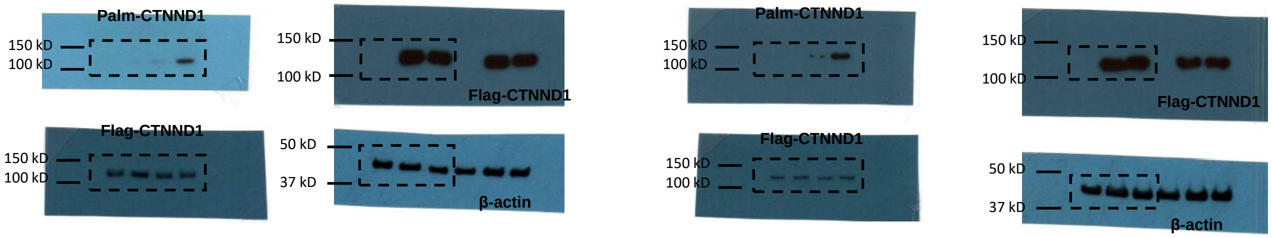

Figure S7

C

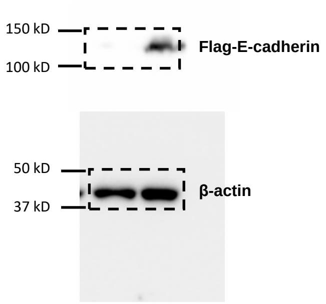

Figure S8

C

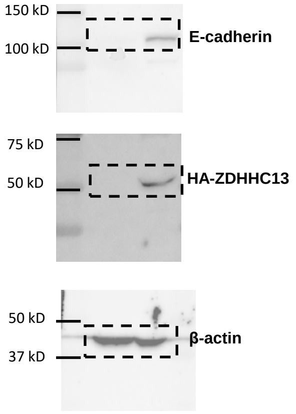

Figure S8

I

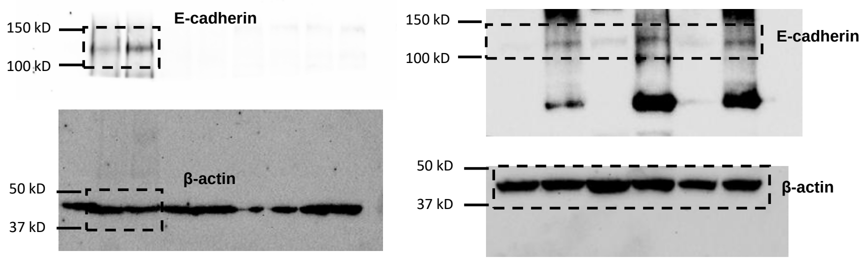

L

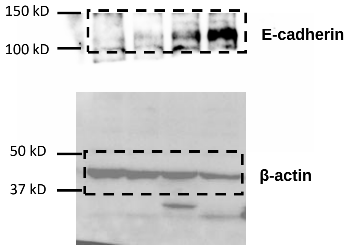

Figure S17

D

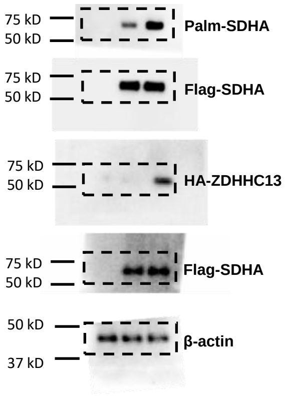

E

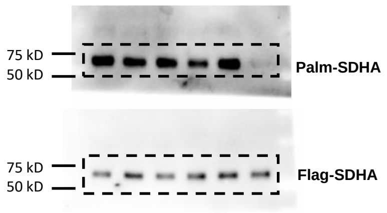

F

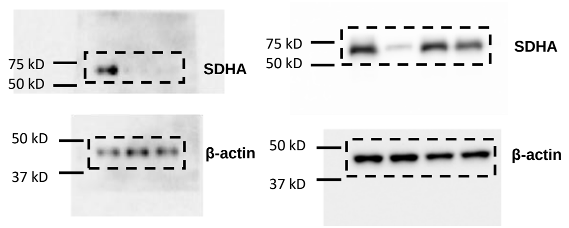

Supplement: Unedited blot and gel images [file jci-135-188249-s141.pdf]
